# Structures of active melanocortin-4 receptor–Gs-protein complexes with NDP-α-MSH and setmelanotide

**DOI:** 10.1038/s41422-021-00569-8

**Published:** 2021-09-24

**Authors:** Nicolas A. Heyder, Gunnar Kleinau, David Speck, Andrea Schmidt, Sarah Zschunke, Michal Szczepek, Brian Bauer, Anja Koch, Monique Gallandi, Dennis Kwiatkowski, Jörg Bürger, Thorsten Mielke, Annette G. Beck-Sickinger, Peter W. Hildebrand, Christian M. T. Spahn, Daniel Hilger, Magdalena Schacherl, Heike Biebermann, Tarek Hilal, Peter Kühnen, Brian K. Kobilka, Patrick Scheerer

**Affiliations:** 1 Charité – Universitätsmedizin Berlin, corporate member of Freie Universität Berlin and Humboldt-Universität zu Berlin, Institute of Medical Physics and Biophysics, Group Protein X-ray Crystallography and Signal Transduction, Charitéplatz 1, Berlin, Germany; 2 Charité – Universitätsmedizin Berlin, corporate member of Freie Universität Berlin and Humboldt-Universität zu Berlin, Institute for Experimental Pediatric Endocrinology, Berlin, Germany; 3 Charité – Universitätsmedizin Berlin, Institute of Medical Physics and Biophysics, Berlin, Germany; 4 Microscopy and Cryo-Electron Microscopy Service Group, Max-Planck-Institut für Molekulare Genetik, Berlin, Germany; 5 Faculty of Life Sciences, Institute of Biochemistry, Leipzig University, Leipzig, Germany; 6 Institute for Medical Physics and Biophysics, Medical Faculty, Leipzig University, Leipzig, Germany; 7 Berlin Institute of Health at Charité – Universitätsmedizin Berlin, Core Facility Genomics, Charitéplatz 1, Berlin, Germany; 8 Department of Pharmaceutical Chemistry, Philipps-University Marburg, Marburg, Germany; 9 Research Center of Electron Microscopy and Core Facility BioSupraMol, Institute of Chemistry and Biochemistry, Freie Universität Berlin, Berlin, Germany; 10 Department of Molecular and Cellular Physiology, Stanford University School of Medicine, Stanford, CA USA; 11 DZHK (German Centre for Cardiovascular Research), partner site Berlin, Berlin, Germany

**Keywords:** Cryoelectron microscopy, Cell signalling

## Abstract

The melanocortin-4 receptor (MC4R), a hypothalamic master regulator of energy homeostasis and appetite, is a class A G-protein-coupled receptor and a prime target for the pharmacological treatment of obesity. Here, we present cryo-electron microscopy structures of MC4R–Gs-protein complexes with two drugs recently approved by the FDA, the peptide agonists NDP-α-MSH and setmelanotide, with 2.9 Å and 2.6 Å resolution. Together with signaling data from structure-derived MC4R mutants, the complex structures reveal the agonist-induced origin of transmembrane helix (TM) 6-regulated receptor activation. The ligand-binding modes of NDP-α-MSH, a high-affinity linear variant of the endogenous agonist α-MSH, and setmelanotide, a cyclic anti-obesity drug with biased signaling toward Gq/11, underline the key role of TM3 in ligand-specific interactions and of calcium ion as a ligand-adaptable cofactor. The agonist-specific TM3 interplay subsequently impacts receptor–Gs-protein interfaces at intracellular loop 2, which also regulates the G-protein coupling profile of this promiscuous receptor. Finally, our structures reveal mechanistic details of MC4R activation/inhibition, and provide important insights into the regulation of the receptor signaling profile which will facilitate the development of tailored anti-obesity drugs.

## Introduction

The melanocortin-4 receptor (MC4R) is one of five human melanocortin receptor subtypes (MC1−5R) that share a set of similar peptidic ligands and constitute an evolutionarily related group of class A G-protein-coupled receptors (GPCRs). MCRs regulate energy homeostasis, pigmentation, cardiovascular function, and sexual functions.^1^ In particular, the MC4R plays a central role in energy balance and appetite regulation.^2^ Naturally-occurring human MC4R mutants are the most frequent monogenic cause of obesity, with ~160 identified variants so far.^3^

Activation of MC4R by its natural agonists α-melanocyte-stimulating hormone (α-MSH) or β-MSH leads to appetite-reducing effects. In contrast, binding of the endogenous inverse agonist agouti-related peptide (AgRP) causes orexigenic effects^4^ by reducing high levels of basal signaling activity.^5^

Besides stimulation of heterotrimeric Gsαβγ protein (Gs), MC4R can elicit other signaling pathways associated with the recruitment of arrestin, Gi, or Gq/11.^6^

To date, pharmacological approaches targeting MC4R have failed mostly due to the severe adverse effects of drug candidates caused, for instance, by a lack of MCR subtype selectivity or G-protein pathway specificity.

Setmelanotide (also termed RM-493, BIM-22493 or IRC-022493)^7–9^ is the first FDA-approved (2020) medication (brand name *Imcivree*) for the treatment of rare genetic conditions resulting in obesity, including pro-opiomelanocortin deficiency (POMC), proprotein subtilisin/kexin type 1 deficiency (PCSK1), and leptin receptor deficiency (LEPR).^10^ Setmelanotide is a cyclic high-affinity peptide with a G-protein signaling profile biased towards Gq/11 (phospholipase C (PLC) activation)^11^ and a 20-fold receptor subtype selectivity towards MC4R compared to natural α-MSH.^12^ In contrast to other MC4R agonists, setmelanotide does not cause common Gs signaling-related adverse effects, such as tachycardia or hypertension. Although rare, moderate adverse effects, such as skin hyperpigmentation, have been reported.

Another FDA-approved (2019), synthetic but non-selective MCR agonist, NDP-α-MSH (also termed afamelanotide; brand name *Scenesse*), is a linear high-affinity analog of α-MSH^13^ for the treatment of MC1R-driven melanogenesis, thereby preventing skin damage from sun exposure (phototoxicity) in individuals with erythropoietic protoporphyria. Moderate adverse effects of NDP-α-MSH exposure include headache, nasopharyngitis, or back pain.^14^

The numerous adverse effects of known MCR ligands call for the discovery of more selective ligands for specific MCR subtypes, particularly the MC4R, as the globally increasing prevalence of human obesity is a growing medical and socioeconomic problem.^15^

Accordingly, a comprehensive understanding of MC4R-mediated signaling regulation is of fundamental importance. To explore the structural basis of agonist action and receptor-mediated signaling, we determined two cryo-electron microscopy (cryo-EM) structures of human wild-type MC4R–Gs-protein complexes bound to agonists setmelanotide and NDP-α-MSH, with resolutions of 2.6 Å and 2.9 Å, respectively. Comparison of our active structures with the recently solved MC4R crystal structure bound to an antagonist^16^ demonstrates the essential role of a calcium ion in forming a link between ligands and TM2 and TM3. In addition, the allosteric connection between the ligand-binding pocket (LBP) and the G-protein-binding cavity (GBC) is mediated by TM3 and facilitated by TM6. Our structural insights combined with signaling data from site-directed mutagenesis reveal mechanistic details of receptor activation and inhibition.

## Results

### MC4R–Gs complex formation with agonists NDP-α-MSH and setmelanotide

**Fig. 1:**
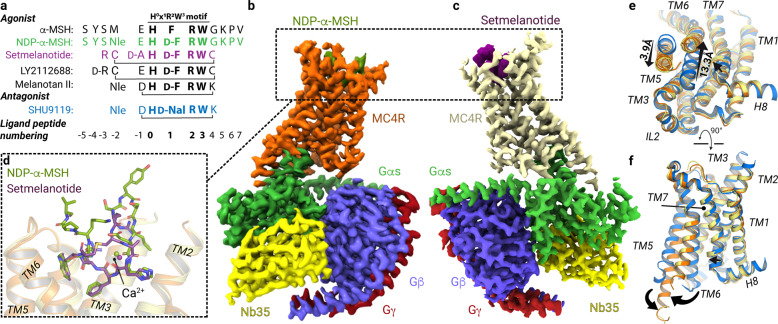
Cryo-EM complex structures of MC4R–Gs bound with NDP-α-MSH and setmelanotide. **a** Sequence alignment of MC4R agonists and antagonist SHU9119. For comparison and simplification, a ligand unifying numbering system is introduced based on the α-MSH core HxRW motif and indicated in superscript for each peptide residue. **b**, **c** Cryo-EM densities of MC4R–Gs complexes stabilized by Nb35 and bound agonists, displayed from mirrored perspectives. Gβ, dark blue; Gγ, red; Nb35, yellow. **d** Superposition of both agonists. **e**, **f** Comparison between SHU9119-antagonized (blue) MC4R^16^ and the active-state structures, either from the cytoplasmic view (**e**) or from inside the membrane plane (**f**). Arrows indicate significant relative spatial differences of transmembrane helices (TM5, TM6, and TM7).

To determine the cryo-EM structure of MC4R-signaling complexes with different agonists, we used NDP-α-MSH and setmelanotide due to their clinical significance and high in vitro binding potency (*K*_i_ = 0.7 nM^16^ and 2.1 nM,^12^ respectively) compared with α-MSH (*K*_i_ = 51 nM^16^) which was essential for generating stable complex samples. Both ligands differ in their general peptide structure. NDP-α-MSH is a linear 13-amino acid peptide and is only modified at two positions (methionine (M4) to norleucine (Nle) and l-phenylalanine (l-F) to d-phenylalanine (d-F)) compared to α-MSH^13^ (Fig. 1a). In contrast, setmelanotide is a cyclic 8-amino acid peptide, which resembles α-MSH at only three positions, in the central H^0^x^1^R^2^W^3^ core motif (Fig. 1a) (a unifying MCR ligand peptide numbering scheme based on the conserved H^0^x^1^R^2^W^3^ motif is introduced in Fig. 1a and is used throughout the text).

We confirmed the binding potency of both ligands using a nano-luciferase-based BRET assay (Supplementary information, Fig. S1).^17^ With our established workflow using *Sf*9 insect cells, we produced the human wild-type MC4R without modifications in sufficient protein amounts for assembling a complex with the Gs-protein (Supplementary information, Fig. S2). However, there was a strong tendency for the MC4R to oligomerize after detergent-based extraction from membranes. This oligomer population was reduced by directly forming the complex with detergent-purified Gs-protein^18^ and MC4R that remained membrane embedded.^19^ After stabilizing the complex through the addition of nanobody-35 (Nb35)^18^ and nucleotide hydrolase apyrase, the resulting nucleotide-free agonist-bound MC4R–Gs–Nb35 complexes were extracted from membranes by solubilization and purified (Supplementary information, Fig. S2). The stable complexes with either agonist NDP-α-MSH or setmelanotide were used for cryo-EM grid preparation and single-particle analysis, yielding cryo-EM maps with a global resolution of 2.9 Å and 2.6 Å, respectively (Fig. 1b, c; Supplementary information, Figs. S3–S6 and Table S1; Materials and Methods).

### Overall structure of MC4R–Gs complexes bound with agonists NDP-α-MSH and setmelanotide

The cryo-EM maps revealed well-defined densities that facilitate unambiguous modeling of the secondary structure and side chain orientations of the MC4R–Gs complex as well as the cofactor ion calcium (Ca^2+^), several water molecules, and the agonist peptides NDP-α-MSH and setmelanotide bound to their respective LBPs (Fig. 1b–d; Supplementary information, Figs. S7, S8).

Only a few components known for their high flexibility, such as the receptor N- (Ntt) and C-termini (Ctt), the intracellular loop (IL) 1, IL3, and the extracellular loop (EL) 1, as well as the alpha-helical domain of Gαs, were not fully built into the final map (modeled residues are listed in Materials and Methods).

**Fig. 2:**
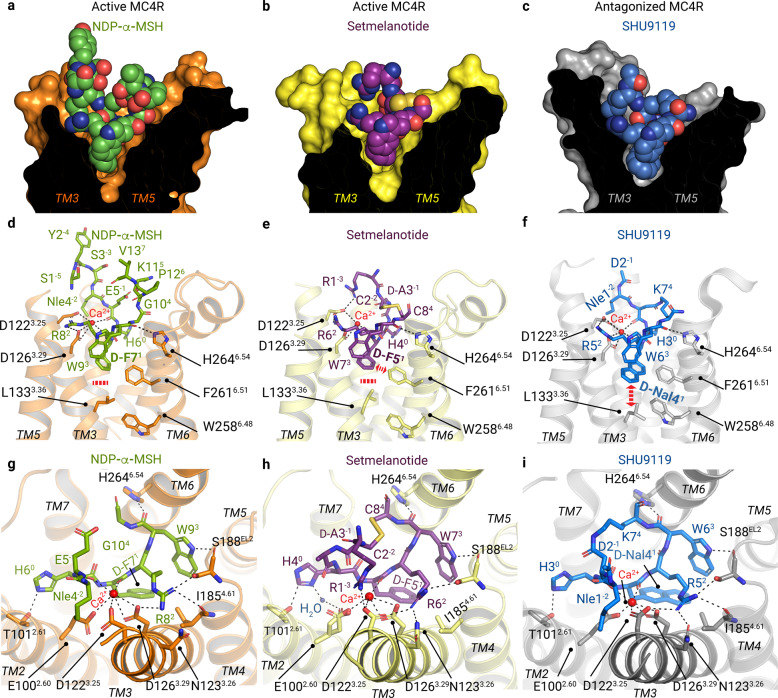
Binding modes of peptidic ligands and calcium at MC4R. **a**–**c** Sphere representations of NDP-α-MSH (green carbon atoms), setmelanotide (purple carbon atoms), and SHU9119 (blue carbon atoms) bound into their respective binding sites in MC4R (clipped surface, orange, light-yellow, gray, respectively). **d**–**f** TM3 and TM6 receptor residues involved in peptide and/or Ca^2+^ interactions are highlighted. The bold red dashed line in **d**, **e** indicates no interactions of L133^3.36^ with agonist residue d-F^1^; the red dashed bidirectional arrow in **e** indicates a hydrophobic interaction between F261^6.51^ and setmelanotide d-F5^1^ (Supplementary information, Table S6), and in **f** indicates an interaction between d-Nal4^1^ of the antagonist SHU9119 and L133^3.36^. **g**–**i** Top view of **d**–**f**, highlighting all intermolecular hydrophilic interactions, assessed by a minimum distance of 3.5 Å (black dashed lines) (Supplementary information, Figs. S9–S11 and Tables S2–S5). NDP-α-MSH residues 4–11 are shown (**g**).

Both agonists are bound foremost with the H^0^x^1^R^2^W^3^ motif between the ELs and within the transmembrane bundle of the receptor (Fig. 1d). The ligands engage MC4R through extensive van der Waals, hydrophobic, and polar interactions, with residues in the transmembrane helices (TMs) as well as EL2 (Supplementary information, Figs. S9–S11 and Tables S2–S6). Specific structural characteristics of MC4R result in a wide-opened extracellular vestibule of the LBP for large peptidic ligands (Fig. 2a–c).

First, the EL2 is only four amino acids long and does not include a cysteine, significantly different from most other class A GPCRs^20^ (sequence alignment in Supplementary information, Fig. S12). Consequently, the highly conserved disulfide bridge between EL2 and TM3^20^ is absent. Instead of C^3.25^ (superscripted numbers according to the unifying Ballesteros & Weinstein numbering for class A GPCRs^21^) in TM3, MC4R has an aspartate (D122^3.25^), which forms part of a ligand and Ca^2+^-binding network (Fig. 2d–i).

Second, a disulfide bridge between C271^EL3^ and C277^EL3^ in EL3 causes a specific helical conformation of the EL3–TM7 transition, which has been only structurally described for two lipidic evolutionary related GPCRs (Supplementary information, Fig. S13a, b). A second observed MC4R disulfide bridge between C279^7.30^ (in EL3) and C40^Ntt^ in the N-terminus (Supplementary information, Fig. S13c) agrees with previous experimental studies on MC4R and MC1R^22^ and stabilizes the EL3-Ntt interplay, thereby contributing to the formation of the extracellular LBP.

Third, in contrast to most other class A GPCRs, the MC4R has no proline in TM2 (Supplementary information, Figs. S12b, S13d), nor the highly conserved proline P^5.50^ in TM5 (Supplementary information, Figs. S12c, S14), which usually causes a kink and bulge, but also a slight rotation of the TMs. This is exemplified by a ~6 Å shift of the extracellular TM2 towards the membrane compared to other known structures and a straight MC4R TM5 shows significant differences to GPCRs with a proline (Supplementary information, Fig. S14), finally modifying the TM5–TM3 interface and together forming the MC4R ligand-binding vestibule.

**Fig. 3:**
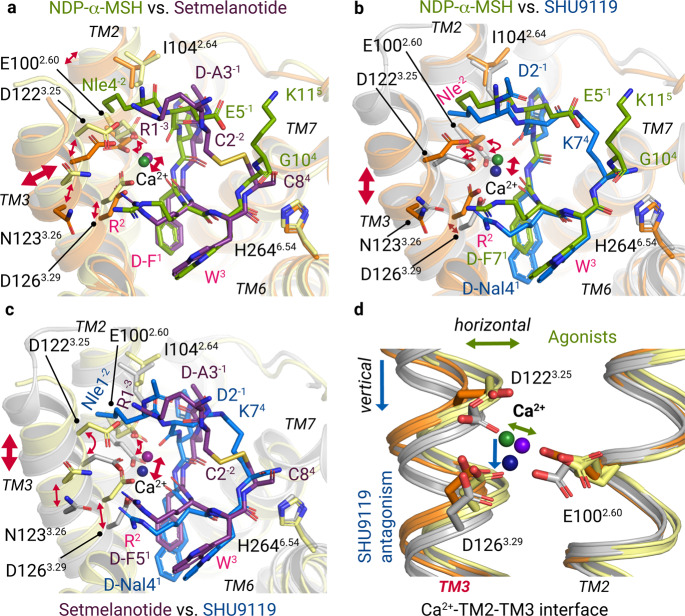
Differential Ca^2+^ and ligand binding at TM3. Superpositions of NDP-α-MSH (residues 4–11), setmelanotide, and SHU9119 bound at MC4R (orange, light-yellow, and gray backbone cartoons). **a** Superposition of both agonists. Residues that are shared by the agonists are labeled in pink. **b**, **c** The ligand-binding mode of SHU9119 is compared with NDP-α-MSH (**b**) and setmelanotide (**c**). Bi-directional red arrows indicate differences in the relative spatial positioning of residues, the TM3, or the Ca^2+^ ion. Detailed intermolecular interactions are summarized in Supplementary information, Tables S5 and S6. **d** The relative alterations of TM3 orientation are also found in Ca^2+^ and its interacting residues of the EDD motif.

In addition, the MC4R–ligand complexes unravel the previously known contribution of Ca^2+^ as a cofactor^23,24^ and offer novel insights into its impact on the respective ligand-binding modes (Figs. 2d–i, 3).

Both the wide extracellular vestibule and the cofactor calcium define a unique LBP, which is adapted to integrate signals from ligands with differences in size and to induce specific cellular responses via different G-protein signaling pathways.

Comparison of the active state structures with the antagonized MC4R structure reveals differences of side chain orientations in the highly conserved amino acid motifs CWxP^6.50^ (C257^6.47^−W258^6.48^−P260^6.50^ in TM6), N(D)P^7.50^xxY (D298^7.49^−P299^7.50^−Y302^7.53^ in TM7) and DR^3.50^Y (D146^3.49^−R147^3.50^−Y148^3.51^ in TM3). These active state-associated changes in the conserved motifs are related to the binding of agonists and G-protein and are linked to global helical movements in the receptor structure upon activation. These mainly include an inward twisted TM5 and a ~13 Å outward dislocated TM6 compared to the inactivated structure, a hallmark of receptor activation that opens the binding cavity for G-proteins (Fig. 1e, f).

### Similarities in agonist binding at MC4R

Comparison of the two MC4R structures bound to the agonists NDP-α-MSH and setmelanotide and the previously published MC4R structure in complex with the antagonist SHU9119^16^ shows that all ligands are buried deeply in the extracellular receptor region between the TMs, but with differently shaped LBPs (Fig. 2a–c). These unique shapes are caused by varying ligand residues (Fig. 1a) and the fact that SHU9119 and setmelanotide are cyclic and more compact in contrast to the linear peptide NDP-α-MSH (Figs. 1d, 2).

All three ligands share a common central amino acid motif H^0^x^1^R^2^W^3^. This four-finger-like motif is essential for the formation of the most relevant interactions within the LBP. The x^1^ position is always located at the bottom part of the LBP (Fig. 2d–f). NDP-α-MSH and setmelanotide have the stereoisomer d-phenylalanine (d-F^1^) instead of an l-F at position x^1^. This substitution is important for the increased potency of NDP-α-MSH compared with α-MSH.^13^

The H^0^x^1^R^2^W^3^ motif forms similar interactions for both agonists, including hydrogen bonds between H^0^ and T101^2.61^ in TM2 and W^3^ with S188^EL2^ in EL2 and H264^6.54^ in TM6 (Fig. 2g, h). The ligands also share similar hydrophobic contacts, such as H^0^ to F284^7.35^−L288^7.39^−F51^1.39^, or d-F^1^ to I129^3.32^ and W^3^ to Y268^6.58^−I194^5.40^−L197^5.43^ (Supplementary information, Figs. S9, S10; complete list in Supplementary information, Table S6).

**Fig. 4:**
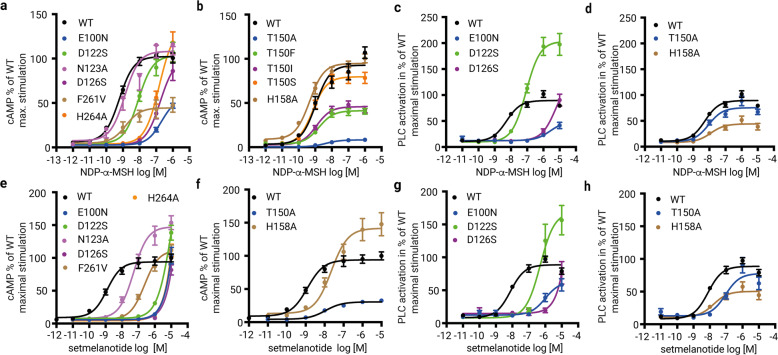
Gs and Gq/11 signaling of MC4R mutants with amino acid substitutions in the ligand- and G-protein-binding regions. **a**–**h** Signaling data for MC4R variants harboring mutations in the ligand-binding region (**a**, **c**, **e**, **g**) and in the G-protein interface (**b**, **d**, **f**, **h**) are plotted. Concentration-response curves for cAMP accumulation (Gs-activation) upon NDP-α-MSH (**a**, **b**) and setmelanotide (**e**, **f**) stimulation. Concentration-response curves of Gq/11 signaling determined by the activation of PLC of wild-type MC4R (WT) or MC4R mutants upon NDP-α-MSH (**c**, **d**) and setmelanotide (**g**, **h**) stimulation. Additional signaling data are shown in Supplementary information, Fig. S15, including α-MSH-induced MC4R signaling. E_MAX_ and EC_50_ values are summarized in Supplementary information, Tables S7−S10.

In both agonist-bound structures, the d-F^1^ at position x^1^ stabilizes Ca^2+^ through a main chain interaction (Figs. 1a, 2g, h), while the side chain points into the core of the receptor and is surrounded by hydrophobic amino acids, namely C130^3.32^, L133^3.35^, I185^4.61^, L197^5.34^, F261^6.51^, and L288^7.39^. Functional characterization of substitutions at these positions and others in the extended LBP is provided in Fig. 4, Supplementary information, Fig. S15 and Tables S7–S9 (binding affinities and EC_50_ values for several MC4R ligands are referenced in Supplementary information, Table S10). The hydrophobic residues I291^7.42^ and L133^3.35^ are close to W258^6.48^ at the highly conserved CWxP^6.50^ motif in TM6 that is often in contact with ligands in several class A GPCRs (e.g., muscarinic acetylcholine receptor–G11 complex,^25^ 5-HT2A serotonin receptor–Gq complex.^26^) In contrast, for MC4R no direct interactions between the ligands and W258^6.48^ exist (Fig. 2d, e). Overall, both agonists have a set of similar receptor contacts specifically to TM3 and TM6, although the interaction pattern of the receptor and agonists with the cofactor calcium is in fact different.

### Calcium is an essential link between the ligands and MC4R

In both agonist-bound MC4R structures presented here, Ca^2+^ is attached centrally between and TM2, TM3, and the peptide agonists. This observation is in line with recent reports on MC4R structures and calcium-binding studies.^16,27^ In case of NDP-α-MSH, Ca^2+^ is five-fold coordinated by main chain interactions with E5^−1^ and d-F7^1^ and receptor side chain interactions with E100^2.60^, D122^3.25^, and D126^3.29^ (Fig. 2g). The latter three residues constitute the EDD motif in TM2 and TM3.

To verify the relevance of the ligand-interface between TM2 and TM3, we generated serine or asparagine mutations (retained hydrophilic side chains) of E100^2.60^, D122^3.25^, and D126^3.29^, and tested their ability to accumulate cAMP (Gs-induced signaling) upon NDP-α-MSH, setmelanotide or α-MSH stimulation (Fig. 4; Supplementary information, Tables S7, S8). In addition, we also examined the capacities of all three mutants for PLC activation (Gαq/Gα11/Gα14/Gα15 family-induced signaling) by NDP-α-MSH and setmelanotide using an NFAT reporter gene assay (Fig. 4; Supplementary information, Table S9).

Setmelanotide and α-MSH are unable to stimulate Gs signaling at these three receptor variants (Supplementary information, Table S9), which confirms the observed essential role of the EDD motif in ligand binding. Previous mutagenesis studies have already shown that substitution of D126A almost completely abolished and in the case of D122A and E100A, significantly reduced NDP-α-MSH-mediated cAMP signaling.^28,29^ In our Gs signaling data, NDP-α-MSH displays a potency reduction of two orders of magnitude for E100N and D126S, but in contrast to the other two ligands, it only shows a slight EC_50_ reduction at variant D122S (9 nM instead of 1.2 nM for wild-type MC4R) (Fig. 4a, e), which suggests differences in the detailed binding modes. This observation is further supported by the previous finding that α-MSH, unlike NDP-α-MSH, does not induce signaling at the D122A MC4R variant.^30^

The PLC signaling data of the EDD motif mutants (Fig. 4c, g) revealed similar tendencies toward inactivation as observed for the Gs signaling, thus suggesting a significant role of these receptor residues also in mediating PLC stimulation.

We here also tested how NDP-α-MSH and setmelanotide stimulations at the wild-type MC4R differ between the two signaling pathways. Both ligands show similar potencies for the induction of cAMP accumulation and PLC activation but deviate in their efficacies (Supplementary information, Fig. S15k, l). NDP-α-MSH is more efficacious in Gs-activation, whereas setmelanotide is more efficacious in PLC activation.

### Specificities of setmelanotide interactions with calcium and MC4R

Compared to NDP-α-MSH, setmelanotide forms unique interactions with Ca^2+^ and TM3 residues in MC4R. The setmelanotide specific R1^−3^ plays a key role beside R6^2^ and binds to D122^3.25^ (Fig. 2h). This structural observation is confirmed by Gs signaling data of the D122S variant that displays a total loss of Gs signaling upon stimulation with setmelanotide, but only a slight reduction for NDP-α-MSH (Fig. 4a, e). Together, both residues R1^−3^ and R6^2^ constitute a tight arginine clamp in hydrogen bond distance to D122^3.25^ of the EDD motif, enabled by cyclization of the peptide. This arginine clamp has three consequences. Firstly, in contrast to the NDP-α-MSH complex, D122^3.25^ is fully oriented towards Ca^2+^, verified by the cryo-EM density map. Secondly, the side chain of R6^2^ shows a slightly different orientation as observed in the NDP-α-MSH–MC4R structure, i.e., more toward N123^3.26^ in TM3 and away from TM4 (Fig. 2g, h). The importance of this setmelanotide-specific interaction to N123^3.26^ is confirmed by a reduced potency for Gs-coupling of MC4R mutant N123A, which is not observed for NDP-α-MSH (Fig. 4a, e). This structural difference is accompanied by a slight horizontal TM3 shift in the setmelanotide–MC4R complex, leading to a ligand-dependent Ca^2+^ positioning (Fig. 3a, d). Thirdly, the setmelanotide interaction between R1^−3^ and D122^3.25^ reduces the number of interactions with the cofactor Ca^2+^ involved in stabilizing the peptide–TM2–TM3 interface with a four-fold coordination of the ion in contrast to the five-fold coordinated Ca^2+^ in the NDP-α-MSH-bound receptor (see above). The reduced number of setmelanotide interactions is also related to a double conformation of E100^2.60^, in which one conformation participates in a hydrogen bond network with a water molecule and H4^0^ (Fig. 2h).

Comparison between the peptide sequences of the two synthetic MC4R agonists LY2112688 (Fig. 1a) and setmelanotide suggests that only two residues that differ between the peptides are responsible for setmelanotide‘s capacity to induce more pronounced Gq/11 signaling compared with LY2112688.^11^ LY2112688 deviates by an E3^−1^ instead of d-A3^−1^ and by d-R1^−3^ instead of the l-R1^−3^ that interacts specifically with D122^3.25^ in the setmelanotide–MC4R–Gs complex (Figs. 1a, 2e). These two differences culminate in a ~50-fold more potent induction of Gq/11-mediated signaling by setmelanotide compared to LY2112688.^11^

### Essential features at TM6 requisite for the active MC4R state

In class A GPCRs, a strong outward movement of TM6 (~13 Å in MC4R, measured at M241-Cα) toward the membrane is a hallmark of active-state conformations because it opens the intracellular cavity for G-protein binding (Fig. 1e). This displacement is enabled and structurally apparent by increasing the kink formation at the highly conserved CWxP^6.50^ motif (_257_CWAP_260_ in MC4R), which is essential for the previously proposed “toggle switch” activation mechanism in different GPCRs.^31^ Comparison of the MC4R with known (in-)active class A GPCR structures revealed similar changes around the W258^6.48^ of the CWxP^6.50^ motif between the inactive or antagonized versus the active structures as observed, e.g., in rhodopsin^32^ and the 5-HT2A serotonin receptor^26^ (Supplementary information, Fig. S16). Hence, we propose a similar toggle-like TM6 movement during MC4R activation at the CWxP^6.50^ motif.

Two residues in MC4R TM6 are involved in ligand binding upstream of the CWxP^6.50^ motif, namely F261^6.51^ and H264^6.54^. H264^6.54^ is in hydrogen bond distance to the ligand’s backbone oxygen of W^3^ near the extracellular vestibule entrance (Fig. 2g–i). Accordingly, the H264A mutant exhibits a significant decrease in potency (EC_50_) (Fig. 4a, e) for NDP-α-MSH- and setmelanotide-induced cAMP accumulation and completely diminished signaling for the endogenous agonist α-MSH (Supplementary information, Fig. S15e).

F261^6.51^does not directly interact with NDP-α-MSH but forms a hydrophobic interaction with the d-F^1^ residue in the setmelanotide–MC4R structure (Fig. 2d, e; Supplementary information, Table S6). This observed structural deviation is confirmed by Gs signaling data for the F261V mutation. Here, a potency reduction in the cAMP accumulation was observed for setmelanotide (29 nM) compared to wild-type (1.2 nM) in contrast to a nearly unchanged potency for NDP-α-MSH. However, the efficacy of setmelanotide was comparable to wild-type MC4R but significantly reduced for NDP-α-MSH (Fig. 4a, e; Supplementary information, Tables S7, S8).

**Fig. 5:**
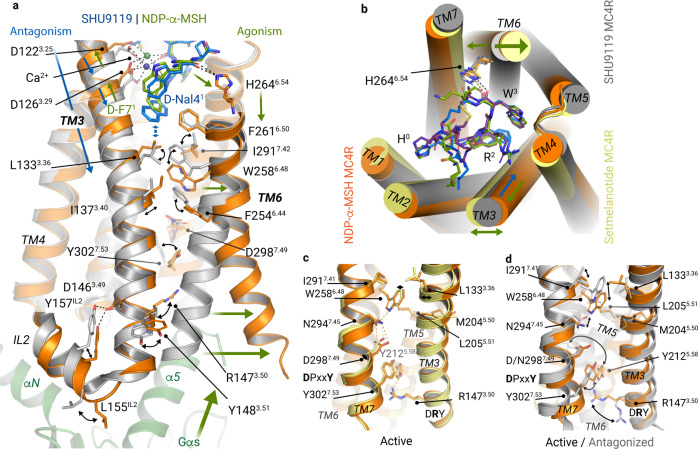
MC4R receptor activation is mediated by TM3 and facilitated by TM6. **a** Superposition of the agonist NDP-α-MSH (green/orange) and the antagonist SHU9119 (blue/gray) bound to MC4R. The supposed activation-pathway from the LBP to the Gs-protein interface is highlighted by green arrows and is triggered by ligand interactions at TM6, inducing the TM6 opening around W258^6.48^. Antagonistic action is facilitated by the interplay of d-Nal4^1^ with L133^3.36^ (indicated by blue arrows) and a subsequently blocked “toggle switch” at W258^6.48^. **b** Top view on ligand pockets highlights (arrows) the relative positioning of transmembrane helices TM1–6 (antagonized versus active structures). **c**, **d** Comparison of active and antagonized MC4R structures. **d** Superposition of active NDP-α-MSH–MC4R and SHU9119–MC4R structures. Relative movements, at the CWxP^6.50^, P^5.50^(M)IF, N(D)P^7.50^xxY, and DR^3.50^Y motifs, that accompany the receptor activation are highlighted by black arrows. Key residues involved in receptor activation are shown as sticks.

This implies that the H264^6.54^–F261^6.51^ region acts as an initial agonist-dependent trigger, with H264^6.54^ directly contacting both agonists, while F261^6.51^ specifically interacts with setmelanotide. Generally, this trigger seems to play an essential role in relaying the signal towards the adjacent helix-tilting TM6–CWxP^6.50^ region (Fig. 5a, c, d).

The W258A mutation in the CWxP^6.50^ motif of MC4R leads to a significant reduction but not a total loss in Gs signaling (Supplementary information, Fig. S15o, p, and^28^). This is accompanied by a reduced cell surface expression (Supplementary information, Fig. S15b), suggesting a loss-of-structural integrity of this mutant. The W258F variant, on the other hand, is expressed at the cell surface like the wild type but shows a higher basal receptor activity and a slightly reduced ligand-stimulated Gs signaling compared to the wild-type MC4R (Supplementary information, Fig. S15f). The importance of an aromatic residue at this position is further underlined by the fact that ~70% of non-olfactory class A GPCRs contain a W^6.48^ and ~16% a F^6.48^.

Altogether, the W258^6.48^ is shifted between the antagonized and the active state structures (Supplementary information, Fig. S16) and is significant for signaling regulation. However, this tryptophan is not the only key switch for MC4R signal transduction in the transmembrane core, where additional amino acids contribute to receptor activation.

### Key sites for signal propagation at the transmembrane core

The MC4R-CWxP^6.50^ motif is spatially surrounded by an extended hydrophobic environment constituted by I137^3.40^, F201^5.47^, F254^6.44^, F261^6.51^, and I291^7.42^, of which only F254^6.44^ and I291^7.42^ display significant displacements comparing the antagonized with the agonized structures (Fig. 5a). It is important to note that in most other class A GPCRs, only small amino acids such as alanine or glycine are present at position 7.42 (Supplementary information, Fig. S12e), and only a few examples such as the ghrelin or thyrotropin receptors (that both also possess high basal activity in Gs signaling) have larger aromatic amino acids there. In MC4R, the mutant I291A cannot induce cAMP signaling, while substitution of I291 by the larger hydrophobic phenylalanine side chain partially retained signaling (Supplementary information, Fig. S15f). This observation, together with the specific interaction of I291^7.42^ to W258^6.48^ observed in the agonist-bound structures, implies a hydrophobic interplay between positions 7.42 and 6.48 in the MC4R that is mandatory for regulation of receptor activation.

In addition, F254^6.44^ (TM6) together with I137^3.40^ (TM3) and M204^5.50^ (TM5) constitute an M^5.50^IF motif in MC4R, reminiscent of the class A GPCR-typical P^5.50^IF motif (P^5.50^−I^3.40^−F^6.44^) involved in the maintenance of an inactive receptor state.^33^ Interestingly, all substitutions of residues in the MC4R-M^5.50^IF motif (I137A, I137F, F254A, F254M, and M204A) do not affect agonist-induced signaling (Supplementary information, Fig. S15g, o–r and Tables S7, S8). However, M204^5.50^ causes a straight, helical conformation of TM5, whereas most other class A GPCRs contain a P^5.50^ at this position (~80% conserved) that induces a kink/bulge in the transmembrane helix^34^ (Supplementary information, Fig. S14). In addition, the MC4R variants harboring M204A substitution or L205F mutation of the neighboring amino acid show significantly increased basal signaling compared to wild-type MC4R (Supplementary information, Fig. S15g), which indicates that this TM5 segment is crucial for regulating basal signaling activity.

Despite the hydrophobic interaction with I291^7.42^ in TM7, W258^6.48^ is also connected in the active state via a hydrogen bond to N294^7.45^, which in turn is coupled to D298^7.49^ of the N(D)P^7.50^xxY motif in TM7 (Fig. 5c). This interaction shifts the N(D)P^7.50^xxY motif slightly toward the GBC (Fig. 5c), accompanied by a rotation of Y302^7.53^ in the direction of Y212^5.58^ in TM5. This orientates Y212^5.58^ into a position that stabilizes the active conformation of R147^3.50^ in the DR^3.50^Y motif, which is part of the G-protein interface.

### Antagonizing the MC4R by impeding the active state formation

The antagonistic peptide SHU9119, recently co-crystallized with the antagonized MC4R,^16^ is a shortened, modified, and circularized derivative of NDP-α-MSH^35^ (Fig. 1a). The binding mode of SHU9119 is generally like that of NDP-α-MSH. Nearly identical hydrophobic contacts exist in the central H^0^x^1^R^2^W^3^ motif as in NDP-α-MSH (Supplementary information, Fig. S11 and Table S4). W6^3^ is also clamped by H264^6.54^ (TM6) and S188^EL2^, and R5^2^ connects TM3−TM4 and EL2 through hydrogen bonds with S188^EL2^ and I185^4.61^ (Fig. 2i; Supplementary information, Fig. S11 and Table S4). All three ligands, therefore, show a similar interaction pattern in the upper part of the LBP, including the participation of Ca^2+^ in the interaction with the EDD motif. This raises the question of how SHU9119 antagonizes the MC4R.

This can only be explained by the action of the unnatural amino acid d-naphthylalanine (d-Nal4^1^) in SHU9119 at position x^1^ in the H^0^x^1^R^2^W^3^ motif that corresponds to d-F^1^ in NDP-α-MSH and setmelanotide (Figs. 1a, 2d–f). In contrast to the active state structures presented here, the bulky aromatic side chain of d-Nal4^1^ of SHU9119 pushes the side chain L133^3.36^ down, accompanied by a vertical downward shift in the upper half of TM3 of ~1 Å (Fig. 5a). Thereby, the side chain of L133^3.36^ is moved in front of W258^6.48^, which eliminates the capacity for the TM6 outward movement (Fig. 5a). This structural observation is functionally supported by the fact that a single mutation of L133^3.36^ to a more flexible and not bulky methionine side chain reverses SHU9119 to an agonist.^36^ Of note, SHU9119 is a partial agonist^35,37^ for the MC1R (with M128^3.36^) and the MC5R (with V126^3.36^) (Supplementary information, Figs. S12b, S17), respectively. Complementarily, melanotan II^38^ is almost identical to SHU9119 except it contains a d-F^1^ instead of the d-Nal4^1^ and agonizes MC4R (Fig. 1a). Our functional characterization of MC4R-L133^3.36^ substitutions to either alanine or phenylalanine reveals no impact on NDP-α-MSH signaling, supporting the observation that the smaller d-F^1^ does not affect the position of L133^3.36^ (Supplementary information, Fig. S15o, p and Tables S6, S7).

In summary, SHU9119 shows a similar binding pattern in the upper part of the MC4R LBP compared to the agonists but acts as an antagonist due to the d-Nal4^1^–L133^3.36^ interaction. This comparison supports the key role of the above discussed hydrophobic interplay of W258^6.48^ and I291^7.42^, which are captured in an inactive or antagonized state by the shifted L133^3.36^ (Fig. 5a).

### The intracellular TM3 as a transducer of agonist-induced G-protein binding

**Fig. 6:**
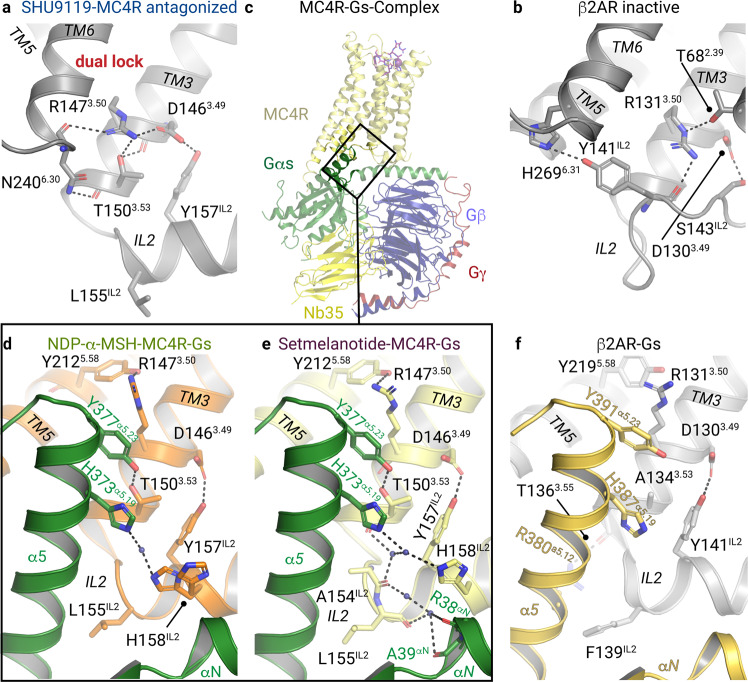
Intracellular interactions in the binding crevice between MC4R and Gs-protein. **a**, **b** Intramolecular interactions at the IL2–TM3–TM6 site stabilizing the antagonized MC4R (PDB ID: 6w25^16^) (**a**), and the inactive β2AR (PDB ID: 2rh1^66^) (**b**). **c**–**e** Overall view on MC4R–Gs with enlarged areas shown in **d** and **e**. Active receptor–Gs complex structures of MC4R bound to NDP-α-MSH (**d**), setmelanotide (**e**). **f** The corresponding boxed region (**b**) in the β2AR–Gs complex (PDB ID: 3sn6^18^) Black dashed lines indicate hydrophilic interactions.

In the antagonized MC4R structure, R147^3.50^ of the DR^3.50^Y motif in TM3 forms potential hydrogen bonds with the T150^3.53^ and D146^3.49^ side chains and with the backbone of N240^6.30^ in the IL3–TM6 transition (Fig. 6a), which contrasts with other inactive class A GPCR structures (e.g. β2AR, Fig. 6c). In addition, the side chain of N240^6.30^ interacts through a hydrogen bond with the backbone oxygen at position T150^3.53^, which suggests an intracellular dual lock formed by hydrogen bonds between TM3 and TM6 that stabilizes the inactive MC4R state (Fig. 6a). Furthermore, IL2 forms a short 3_10_-helix in the inactivated MC4R, which allows for the interaction of D146^3.49^ with Y157^IL2-3.60^ in IL2.

Comparing the active with the antagonized MC4R structure, receptor activation is strongly accompanied by DR^3.50^Y motif side chain rearrangements, considered typical for class A GPCR activation (Fig. 6).^39^ MC4R-R147^3.50^ is stabilized by a hydrogen bond to Y212^5.58^ in TM5, constituting the G-protein cavity base. Extensive interactions in the interface between the activated MC4R and Gs-protein occur via the α5-helix of the Gαs domain and its C-terminal loop, termed C-cap, which has been already described for other GPCR–G-protein complexes.^18,40^

Following disruption of the MC4R TM3–TM6 dual lock by ligand interaction and the TM6 outward tilt (Fig. 6a), both residues, R147^3.50^ and T150^3.53^, acquire a key role in constituting an active state conformation. R147^3.50^ forms a cation−π stacking interaction with the side chain of Y377^α5.23^ (CGN in superscript^41^) in the α5 C-cap.

The side chain of Y377^α5.23^ is further stabilized by a hydrogen bond to T150^3.53^ (Fig. 6d, e), which was involved in stabilization of the inactive state. Such stabilization by an additional hydrogen bond interaction has not been identified in other GPCR–Gs complex structures known so far and highlights T150^3.53^ as a key site in the switching process between inactive and active MC4R conformations. A similar interaction has been observed only between S126^3.53^ of the muscarinic receptor M1 and Y356 ^α5.23^ from the G11-protein.^25^ Of note, most class A GPCRs contain an alanine at the corresponding position of MC4R-T150^3.53^ (Supplementary information, Fig. S18).

Our Gs signaling data for several mutants of T150^3.53^, including the pathogenic substitution T150I,^42,43^ confirm the important role of this intermolecular polar interaction (Fig. 4b, f; Supplementary information, Tables S7, S8). T150^3.53^ substitutions by hydrophobic and acidic residues drastically reduced agonist-induced cAMP accumulation for setmelanotide and NDP-α-MSH (Fig. 4f, g). In contrast, the T150S mutant displays a comparable signaling level for stimulation by NDP-α-MSH (Fig. 4b, f; Supplementary information, Tables S7, S8). Remarkably, in contrast to impaired cAMP accumulation, the maximal Gq/11-coupling (efficacy) for the T150A variant is unaffected upon stimulation with NDP-α-MSH or setmelanotide (Fig. 4d, h), which suggests that the α5 helix-G-protein/TM3-MC4R interface varies between Gq/11 and Gs at T150^3.53^. In contrast, the potency of mutant T150A-stimulated Gq/11 signaling by setmelanotide and NDP-α-MSH differs by 92 nM and 12 nM, respectively (Fig. 4d, h; Supplementary information, Table S9). This setmelanotide-specific reduction in PLC activation indicates a ligand-dependent effect on T150^3.53^ that impacts the G-protein coupling preferences at the TM3–IL2 transition.

### Common and unique G-protein interactions with the IL2

**Fig. 7:**
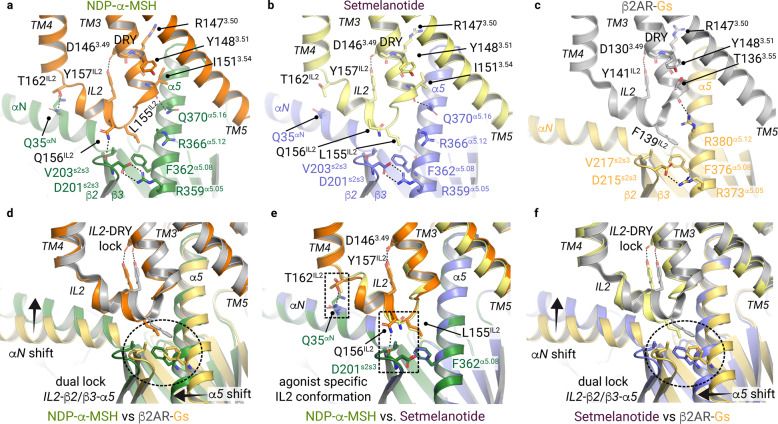
Gs-protein adjustment at IL2. **a**–**c** Display of the binding interface between TM3–TM5 and Gs-protein in the NDP-α-MSH (**a**), setmelanotide (**b**) bound MC4R–Gs complexes, and the β2AR–Gs complex (PDB ID: 3sn6) (**c**). Interactions of IL2 and TM3 are displayed as black dashed lines. **d**–**f** The superposition of NDP-α-MSH–MC4R–Gs and setmelanotide–MC4R–Gs complexes with the β2AR–Gs complex display the IL2–β2/β3–α5 lock of L155 ^IL2^ (MC4R) with V203 and F362 (Gαs), as well as F139 ^IL2^ (β2AR) with V217 and F376 (Gαs) adjusting the position of the α5 helix. A relative α5 shift can be noticed, which results in a slight rotation of the entire Gs coupled to MC4R compared to that coupled to β2AR, most prominently visible by an αN helix shift. **e** Superposition of both agonist-bound MC4R–Gs structures highlight changes of the hydrogen bonds between Gαs and IL2 residues (dashed boxes).

One of the most important interactions between the IL2 of MC4R and Gs is formed by residues L155^IL2-3.58^ and F362^α5.08^ (Fig. 7a, b). In the MC4R–Gs complex, the side chain of L155^IL2-3.58^ is located in the interface between the Gαs αN–β1 junction, the β2–β3 turn, and the α5-helix (Fig. 7a, b). This IL2–β2/β3–α5 lock of L155^IL2-3.58^ is reminiscent of the known F139^IL2-3.58^−F376^α5.08^ interplay in the β2AR–Gs complex (Fig. 7c) or the Gq-coupling interactions at M1 and M3 receptors.^44^

Of note, many class A GPCRs display a leucine or isoleucine at the IL2-3.58 position (Supplementary information, Fig. S18). In agreement with the essential role of IL2 and position 3.58 in Gs-coupling, previously investigated mutants L155A, R147A, T150A, and Y157A have been shown to eliminate any basal signaling activity of MC4R,^45^ presumably by disturbing the interface contacts between the receptor and its effector protein.

Finally, the β2AR–Gs complex shows a tighter α5-helix engagement toward TM5 (Fig. 7d, e), likely caused by differences in the specific IL2-3.58 to Gs-α5.08 interactions. Consequently, several further interactions in the TM3–IL2–α5-helix network differ from the β2AR–Gs complex (Fig. 7a–c), leading to a displaced αN-helix in the active MC4R–Gs complexes (Fig. 7d, f).

Interestingly, subtle differences at the IL2–G-protein interface can be observed between the two agonist–MC4R–Gs complex structures. First, in the NDP-α-MSH–MC4R complex, T162^IL2-3.65^ presumably forms a hydrogen bond to Q35^αN^ in the αN-helix, and Q156^IL2-3.59^ to the backbone of D201^s2s3^ in the β2–β3 turn of Gαs (Fig. 7a, b, e; Supplementary information, Figs. S19, S20). None of these contacts are present in the setmelanotide complex, most likely due to a rotation of the side chains of T162^IL2-3.65^ and Q156^IL2-3.59^ (Fig. 7b; Supplementary information, Figs. S19, S20).

A remarkable difference between the two agonist–MC4R complexes is found in the interactions formed by residue H158^IL2-3.61^ in IL2. In both complexes, H158^IL2-3.61^ is part of a water-mediated interaction network with H373^α5.19^ in the Gs-α5 helix (Fig. 6d, e). However, in the NDP-α-MSH–MC4R–Gs complex, the H158^IL2-3.61^ side chain is more flexible and samples two different rotamer conformations, which is not seen in the setmelanotide–MC4R–Gs complex (Fig. 6d, e; Supplementary information, Figs. S21, S22). In the latter case, there is an additional water-mediated stabilization of H158^IL2-3.61^ involving A154^IL2-3.57^ in IL2 (Fig. 6e).

Interestingly, the H158A mutation leads to constitutive receptor Gs activation (Supplementary information, Tables S7, S8 and^45^) in agreement with the pathogenic gain-of-function mutant H158R^46,47^ and other constitutively activating mutants D146A, F149A, and F152A^45^ in IL2, but does not impair Gs signaling induced by NDP-α-MSH or setmelanotide (Fig. 4b, f). In contrast to the Gs signaling, the maximum Gq/11 signaling of the H158A mutant decreases to 50% compared to wild-type for NDP-α-MSH and setmelanotide stimulation (Fig. 4d, h). Thus, this residue, along with T150^3.53^, is a second identified receptor site where the G-protein signaling profile is shifted by mutation, suggesting distinct roles of the wild-type amino acids upon binding to different G proteins. Finally, our data strongly support an essential role of this IL2 receptor residue for ligand-mediated Gq/11-activation, but barely for Gs-activation.

## Discussion

Here we report cryo-EM structures of active-state MC4R–Gs complexes bound to the FDA-approved peptide agonists NDP-α-MSH and setmelanotide (Fig. 1). Both structures provide details of agonist binding and receptor activation, with further details gained compared to the recently determined antagonized SHU9119–MC4R structure^16^ (Figs. 2, 3). Summarizing the structural findings in combination with signaling data, extracellular binding of the peptide agonists occurs in complex with the cofactor calcium and at key receptor residues in TM2, TM3, and TM6. Here we assign the agonist-dependent activation trigger at TM6 to the ligand-binding region of H264^6.54^–F261^6.51^ directly adjacent to the helix-kink at the CWxP^6.50^ motif, which is involved in the known “toggle-switch” activation mechanism with the resulting TM6 movement typical for class A GPCRs (Fig. 5; Supplementary information, Fig. S23).

Notably, extracellular interactions formed by SHU9119 are not significantly different from those formed by both agonists, but SHU9119 blocks the TM6 movement essential for MC4R signaling in the transmembrane region. SHU9119s antagonism depends on the interaction with L133^3.36^ and is therefore MC4R-selective, since it acts as a partial agonist at MC1R (L^3.36^M)^36^ and MC5R (L^3.36^V) with neither receptor having a leucine at the reciprocal position (Supplementary information, Fig. S12b). This conclusion is further supported by agonistic effects of the ligand melatonan II on MC4R, which differs from the antagonistic SHU9119 in ligand position 1 where it exhibits a smaller d-F versus the larger d-Nal residue in SHU9119 (Fig. 1a). Finally, the recently described cryo-EM structure of the active MC1R–Gs complex bound to SHU9119^48^ (here acting as an agonist) ultimately evidences the significance of position 3.36 for receptor activity regulation and antagonism of SHU9119 at MC4R.^16^

Moreover, the binding modes of agonistic ligands NDP-α-MSH and setmelanotide compared to each other and the antagonist SHU9119 show different orientations of the Ca^2+^-binding site, which is formed by ligand and receptor residues. These differences are potentially accompanied by local shifts in the TM3 adjustment relative to the other helices (Fig. 3).

Compared to the antagonized SHU9119–MC4R crystal structure, we identified several active state-specific conformational changes that play a concerted role in signal transduction, which are in line with a recently reported setmelanotide–MC4R–Gs complex structure^27^ (Supplementary information, Fig. S24), as well as observations in active agonist–MC1R–Gs complexes.^48^ A large movement of TM6 starting at the CWxP^6.50^ motif with altered side chain interactions of residues I291^7.42^ and W258^6.48^, small conformational alterations in TM7 related to local shifts at the N(D)P^7.50^xxY motif, as well as reorientations of Y^5.58^ in TM5, and the conserved DR^3.50^Y motif in TM3 are significant for the active structures. Subsequently, the conserved DR^3.50^Y motif in TM3 and the adjacent IL2 constitute important parts of the interface to the Gs-protein.

T150^3.53^ in the intracellular TM3/IL2 transition of MC4R is essential for Gs-binding as indicated by a direct hydrogen bond to Y377^α5.23^ in the α5 helix of Gs and supported by our mutagenesis data on substitutions against several hydrophobic residues that showed impaired signaling (Fig. 4). In conclusion, T150^3.53^ switches from being a key player in the inactive state, where it stabilizes the TM3–TM6 dual lock (Fig. 6), to a binding partner of the α5 helix in Gs. This finding is remarkable considering the non-conserved nature of this class A GPCR position (Supplementary information, Fig. S12b). The functional significance of T150^3.53^ for Gs-activation cannot be observed for Gq/11-mediated PLC activation in the presence of NDP-α-MSH, and only slightly for setmelanotide, indicating remarkable differences in the binding mode of the various G-protein subtypes (Fig. 4b, d, f, h). The aberrant potencies for Gq/11 signaling of setmelanotide (potency slightly decreased) and NDP-α-MSH (potency like wild-type) at the MC4R-T150A variant are indicators for a fine-tuned allosteric connection between the ligands and the receptor–G-protein interface, probably via TM3 and IL2 with a high, but flexible number of structurally observed interactions (Fig. 7a, b, e). This also suggests that the orientation of the α5 helix from Gq/11 into the GBC of MC4R is probably different from the α5 helix of the Gs-protein, albeit the T150^3.53^ hydrogen bond acceptor Gs-Y377^α5.23^ is also present in Gq (Gq-Y371^α5.23^).

Moreover, the side chain orientation of H158^IL2^ in both agonist-MC4R structures is significantly different depending on the respective ligand (Fig. 6d, e). However, the definitive functional role of H158^IL2^ for Gs-activation cannot be deciphered based on our complex structures, consistent with the rather neutral effect of H158A substitution on ligand-mediated Gs signaling. In contrast, the H158A mutant reduces PLC activation to 50% for both ligands (Fig. 4d, h; Supplementary information, Table S9), indicating that this residue is essential for Gq/11-activation but not for Gs signaling (Fig. 4b, f; Supplementary information, Table S8).

In summary, our data highlight both T150^3.53^ and H158^IL2^ as determinants for regulating the MC4R signaling profile and probably of the associated G-protein coupling specificities.

The interesting observation of a slightly different binding mode between MC4R-IL2 and Gs in both complex structures might be due to modulation of the allosteric link between the LBP and the GBC caused by the specific binding modes of both agonists to MC4R (to TM3, TM4, and the transition to EL2) and calcium. Here, the setmelanotide residue R1^−3^ mediates specific contacts to TM3, which could also be related to the increased efficacy in Gq/11 signaling and thus for the biased pharmaceutical profile of this agonist compared with the MC4R agonist NDP-α-MSH lacking this arginine (Figs. 2, 3; Supplementary information, Table S7).

To fully characterize G-protein subtype selectivity at the MC4R and to further differentiate Gq/11- and Gs-coupling, an agonist–MC4R–Gq/11 complex is required. Other open questions pertaining to the unique MC4R system await structural elucidation, such as determining the apo-state conformation, dimer-arrangements relevant to MC4R as an endocrine GPCR,^49^ and MC4R bound with endogenous ligands such as α-, β-MSH, AgRP or the cofactor protein MRAP.^50^ Altogether, the structural findings presented here will facilitate the development of new MCR subtype- and G-protein-selective anti-obesity drugs.

## Materials and methods

### Construct preparations for cryo-EM

#### Protein expression of human MC4R

Wild-type human MC4R (UniprotKB-P32245) was modified to include an N-terminal hemagglutinin signal sequence, followed by a FLAG-tag epitope (DYKDDDK). The C-terminal eGFP, followed by polyhistidine (His-) and rho-1D4 tags, is removable by HRV-3C protease cleavage (construct name: MC4R–eGFP) and was inserted into a pOET3 vector.

For the production of MC4R–eGFP, recombinant baculovirus was generated by co-transfecting *Sf*9 cells (from *Spodoptera frugiperda*) with pOET3_MC4R–eGFP and linearized BAC10:1629_KO_^51,52^ using Trans-IT Insect (Mirus Bio). *Sf*9 cells were cultured in SF900 II serum-free medium (Invitrogen) at 28 °C for virus generation. A 1 L preparation of *Sf*9 cells at 2 × 10^6^ cells/mL were infected with 10 mL of P2 virus MC4R−eGFP virus. Cultures were grown at 27 °C, harvested by centrifugation 48 h post infection, and stored at −20 °C.

#### Protein expression and purification of Gαsβ1γ2 and Nb35

Bovine Gαs-short subunit (UniprotKB-P04896-2) in pFastbac vector and rat Gβ_1_ (UniprotKB-P54311) and bovine Gγ_2_ (UniprotKB-P63212) subunits in pFastbacDual vector were previously used and described.^18^ Heterotrimeric Gαsβ_1_γ_2_ protein (or named Gs) was expressed in *Trichoplusia ni* (*Tni*) insect cells, maintained in ESF 921^TM^ serum-free insect cell culture media (Expression Systems) at 28 °C. The virus was prepared using Bac-to-Bac^TM^ baculovirus expression system (Thermo Fisher Scientific). The cells were infected with both Gαs and Gβ_1_γ_2_ virus, based on small scale titrations and harvested 48 h post infection, and stored at −20 °C. Gs was purified as described previously.^18^ The single-domain antibody Nanobody-35 (Nb35) was previously described.^18^ Nb35 was expressed in *E. coli* strain WK6, extracted and purified by immobilized metal (Ni-NTA) affinity chromatography according to previously described methods.^18^

#### Complex formation and purification

MC4R–Gs–Nb35 complexes with both agonists NDP-α-MSH and setmelanotide (from now on abbreviated by agonist) were formed in *Sf9* membranes. *Sf9* cell pellets containing MC4R–eGFP were resuspended in 20 mM HEPES, pH 7.5, 50 mM NaCl, 2 mM MgCl_2_, 1 mM CaCl_2_, 25 µM Tris (2-carboxyethyl) phosphine (TCEP), 25 U/mL apyrase (New England Biolabs) 2.5 mg/mL leupeptin (Enzo Life Sciences, Inc.), 0.16 mg/mL benzamidine (Sigma-Aldrich) and 1 µM of the respective agonist (in-house peptide synthesis). For 1 L of cell pellets, Gs pre-incubated with Nb35 was added and incubated overnight at 4 °C. The membrane sample containing agonist–MC4R–Gs–Nb35 complex was collected by centrifugation at 46,000× *g* and carefully resuspended in 20 mM HEPES pH 7.5, 150 mM NaCl, 2 mM MgCl_2_, 1 mM CaCl_2_, 25 µM TCEP, 2.5 mg/mL leupeptin, 0.16 mg/mL bezamidine, 1 µM agonist and 1% n-dodecyl β-d-maltoside (DDM), 0.1% cholesteryl hemisuccinate (CHS) (Anatrace, Inc.). After 2 h, the solubilized protein was separated from insoluble remains by centrifugation at 46,000× *g*. The supernatant was diluted twofold with 20 mM HEPES, pH 7.5, 150 mM NaCl, 2 mM MgCl_2_, 5 mM CaCl_2_, 25 µM TCEP, 2.5 mg/mL leupeptin, 0.16 mg/mL benzamidine and 1 µM agonist. Anti-FLAG M1 resin (Sigma-Aldrich) was added and incubated for 2 h at 4 °C rotating. M1 resin was collected by centrifugation (500× *g*, 5 min) and loaded into a wide-glass column and washed for 5 column volumes with wash buffer (20 mM HEPES, pH 7.5, 150 mM NaCl, 1 mM CaCl_2_, 25 µM TCEP, 1 µM agonist) with 0.1% DDM and 0.01% CHS, followed by a 1 h incubation in wash buffer with 0.8% lauryl maltose neopentyl glycol (LMNG), 0.08% CHS (Anatrace, Inc.), and 0.02% DDM. Subsequently, LMNG/CHS concentration was lowered stepwise to 0.01% LMNG, 0.001% CHS for 1 h. Elution of the complex was initiated by addition of 20 mM HEPES, pH 7.5, 150 mM NaCl, 25 µM TCEP, 1 µM agonist, 0.01% LMNG, 0.001% CHS, 5 mM EDTA and 0.2 mM DYKDDDDK peptide (GenScript Biotech). C-terminal eGFP was removed by adding HRV-3C protease (in-house purified), incubated at 4 °C overnight. After concentration, the agonist–MC4R–Gs–Nb35complex was loaded onto a Superdex 200 Increase 5/150 GL (Sigma-Aldrich). Receptor-containing fractions were concentrated to 5 mg/mL and directly vitrified. One liter of MC4R–eGFP expressing cells yielded 0.25 mg complex.

### Cryo-electron microscopy

#### Cryo-EM sample preparation and image acquisition

Vitrification of NDP-α-MSH– and setmelanotide–MC4R–Gs–Nb35 complexes was conducted immediately after sample preparation at a concentration of 1.2 mg/mL and 5 mg/mL, respectively. 3.8 µL of the sample was applied to glow-discharged holey gold grids (UltrAuFoil R1.2/1.3 300 mesh, Quantifoil Micro Tools GmbH), blotted for 4 s and plunge-frozen in liquid ethane using an FEI Vitrobot Mark IV (Thermo Fisher Scientific) set to 10 °C and 100% humidity.

Images were acquired using a FEI Titan Krios G3i microscope (Thermo Fisher Scientific) operated at 300 kV equipped with a FEI Falcon 3EC detector (Thermo Fisher Scientific) running in counting mode at a nominal magnification of 96,000×, giving a calibrated pixel size of 0.832 Å/px. Movies were recorded for 40.78 s accumulating a total electron dose of 40 e^−^/Å^2^ fractionated into 33 frames. EPU 2.8 was utilized for automated data acquisition with AFIS enabled using a nominal defocus between −0.8 and −2 µm.

A total of 5618 micrographs were collected for NDP-α-MSH–MC4R–Gs–Nb35 and 7583 micrographs for setmelanotide–MC4R–Gs–Nb35. These were used for further image processing. Further details are given in Supplementary information, Table S1.

#### Cryo-EM image processing

The entire data analysis was conducted within the cryoSPARC v2.15 framework (Supplementary information, Figs. S3–S6). Image analysis of the NDP-α-MSH–MC4R–Gs–Nb35 dataset (Supplementary information, Figs. S3, S4) started with movie alignment and dose-weighting using “Patch motion correction” followed by “Patch CTF estimation”. Initial particle picking was done with “Blob picker” using a particle diameter of 180 Å. Particle images were extracted with a box size of 280 px, Fourier-cropped to 70 px (3.328 Å/px). After reference-free 2D classification, selected class averages were used for template-based particle picking with a 170 Å particle mask. A total of 2,746,119 particle images were subjected to two cycles of 2D classification to clean the dataset. Ab initio reconstruction of particle images belonging to shiny classes was applied to generate a reference model for 3D classification, after which 260,451 particle images were selected for further processing. Homogeneous refinement after re-extraction of the particles, Fourier-cropped to 140 px (1.664 Å/px) generated a 3D reconstruction of 3.45 Å global resolution. Another round of heterogeneous refinement was applied to finally select 221,682 particle images for unbinned extraction (280 px, 0.832 Å/px). Iterations of homogeneous refinement and Global CTF refinement were applied to correct for higher-order aberrations yielding a final reconstruction of 2.86 Å resolution after non-uniform (NU) refinement.^53^ Using NU-refinement, masking of the all-helical domain was not necessary to yield a high-resolution map (Fig. 1; Supplementary information, Figs. S3, S4). Processing of the setmelanotide–MC4R–Gs–Nb35 data (Supplementary information, Figs. S5, S6) was done as described for the NDP-α-MSH–MC4R–Gs–Nb35 dataset using the previously generated templates for picking of 4,330,500 particle images. After a single round of 2D classification, 4,267,612 particle images were subjected to two iterative rounds of 3D classification with the NDP-α-MSH–MC4R–Gsαβγ–Nb35 reconstruction as reference filtered to 30 Å. Micrographs with local motions above 10 px or estimated resolutions worse than 4 Å were discarded, leaving a total of 797,185 particle images for another round of 3D classification. Homogeneous refinement of 431,973 particle images after re-extraction with a box size of 280 px (0.832 Å/px) yielded a resolution of 2.82 Å that could be improved to 2.77 Å by CTF refinement. After a final 3D classification, 370,621 particles were selected for NU refinement resulting in a 2.58 Å reconstruction. (Fig. 1; Supplementary information, Fig. S6).

#### Model building and refinement

The of the NDP-α-MSH–MC4R–Gs–Nb35 complex as well as setmelanotide–MC4R–Gs–Nb35 complex were derived from the inactive MC4R structure (PDB ID: 6w25^16^) together with the Gsαβγ–Nb35 complex of the β2-adrenergic receptor–Gs complex (PDB ID: 3sn6^18^) as initial models. Both MC4R complexes were built and adjusted manually using the program COOT.^54^ Model building for the MC4R ligands NDP-α-MSH and setmelanotide was started de novo using COOT.^54^ Local-refined, as well as overall cryo-EM maps were used to add water molecules. After every round of manual refinement and for the final round, Real-space refinement^55^ was performed with the program PHENIX^56^ using geometric restraints, a global minimalization protocol and B-factor refinement. Both models were additionally refined with isotropic B-factors in reciprocal space using REFMAC5^57^ of the CCP4 (Collaborative Computational Project, number 4) software suite.^58^ The refinement was carried out in the resolution range of 233–2.88 Å and 233–2.6 Å for the NDP-α-MSH–MC4R and setmelanotide–MC4R complexes, respectively (Supplementary information, Table S1).

The final model of the NDP-α-MSH–MC4R complex includes the following amino acids (based on the final overall cryo-EM map). MC4R: 40–108, 118–230, 239–316; NDP-α-MSH: 1–13; Gsα-protein: 13–47, 194–236, 249–280, 293–306, 322–380; Gβ_1_: 3–340; Gγ_2_: 9–63; Nb35: 1–128.

The final model of the setmelanotide–MC4R complex includes the following amino acids (based on the final overall cryo-EM map). MC4R: 40–107, 117–230, 240–316; setmelanotide: 1–8; Gsα-protein: 14–47, 193–236, 248–280, 293–310, 318–351, 355–380; Gβ_1_: 4–340; Gγ_2_: 9–63; NB35: 1–128. Structure validation was performed with PHENIX,^56^ MolProbity,^59^ SFCHECK,^60^ and OneDep of the Protein Data Bank.^58^ Potential hydrogen bonds and van der Waals contacts were analyzed using HBPLUS^61^ and LIGPLOT 1.45+.^62^ All structure superpositions of backbone α-carbon traces were performed using the CCP4 program LSQKAB.^63^ All molecular graphics representations in this work were created using the PyMol Molecular Graphics System Version 1.3 (Schrödinger, LLC, New York, NY) and UCSF Chimera.^64^

### Functional MC4R ligand-binding assays

#### Saturation and competition binding assay using NanoLuc^TM^ Luciferase assay (nanoBRET)

Wild-type MC4R was modified to include an N-terminal hemagglutinin signal sequence, followed by Luciferase (NanoLuc^TM^ Luciferase; Promega)^17^ and cloned into pMT4 vector. Human embryonic kidney 293 (HEK293T) cells grown in DMEM/F-12 (Thermo Fisher Scientific) medium (supplemented with l-Glutamin, HEPES, phenol red, sodium pyruvate pH 6.9–7.3) (Thermo Fisher Scientific, Gibco) were transiently transfected using FuGENE^®^ HD transfection reagent (Promega). 20,000 cells per well were seeded in white corning assay 96-well plates. After 24 h, the medium was removed and replaced with 75 µL ligand serial dilutions in Opti-MEM reduced serum media (Thermo Fisher Scientific) without phenol red and incubated for 2 h. For saturation experiments, TAMRA-NDP-α-MSH (TAMRA-NDP) labeled with the fluorophore 5-carboxytetramethylrhodamine (TAMRA) was titrated from 1 µM to 1 pM. For competition experiments, each well contains 10 nM TAMRA-NDP and the competing ligand was titrated from 1 µM to 10 pM for setmelanotide. Non-specific binding was measured by adding 20 µM NDP-α-MSH, to saturate the ligand-binding pocket with the non-fluorescent ligand. After 2 h, 25 µL Furimazine (Promega) was added and incubated for 15 min. Luminescence and resulting bioluminescence resonance energy transfer (BRET) was measured in Spectramax ID3 (Molecular Devices) and CLARIOstar Plus (BMG LABTECH) plate reader with 460 nm (short-pass filter) and 610 nm (long-pass filter). The BRET ratio was calculated by the quotient of long-pass divided by short-pass.^17^ GraphPad PRISM 8 (GraphPad Software Inc.) was used for analysis by sigmoidal dose-response (variable slope) for dose-response measurements and one site - Fit K_i_ for competition experiments.

### Functional characterization by NanoGlo® HiBiT, PLC activation and AlphaScreen™ assays

#### Cell lines, cloning and reagents

HEK293 cell line was purchased from ATCC. Cells were authenticated by single nucleotide polymorphism (SNP) analysis and regularly tested for mycoplasma contamination. Cultivation took place in l-glutamine-containing minimal essential medium MEM (Merck Biochrom) supplemented with 5% fetal bovine serum FBS (Thermo Fisher Scientific, Gibco) and 1% non-essential amino acids NEA (Merck Biochrom) at 37 °C and humidified air containing 5% CO_2_. For cAMP accumulation and PLC activation assays, 1.5 × 10^4^ cells per well were seeded in poly-l-lysine coated (Merck Biochrom) translucent 96-well plates (Falcon) and incubated for 24 h. In an identical fashion, determination of total and cell surface expression (NanoGlo^®^ HiBiT assay, Promega) were performed in white opaque, poly-l-lysine-coated 96-well plates (Corning #3917).

MC4R cDNA was amplified from genomic DNA and cloned into eukaryotic expression vector pcDps. The receptor was N-terminally tagged with the hemagglutinin (5′-YPYDVPDYA-3′) epitope (HA) for cAMP measurements and luciferase-based assays. For NanoGlo^®^ HiBiT assays, MC4R was cloned into pBiT3.1-N (Promega) using *Eco*RI/*Bam*HI restriction sites, resulting in HiBiT protein tag N-terminally spaced by eleven amino acids. All single point mutations were incorporated into the expression vectors using site-directed mutagenesis. Cloned constructs were sequenced and verified with BigDye-terminator sequencing (PerkinElmer Inc.) using an automatic sequencer (ABI 3710 XL; Applied Biosystems). α-MSH, NDP-α-MSH and 3-Isobutyl-1-methylxanthine (IBMX) were purchased from Sigma-Aldrich.

#### Transfection

For determination of cAMP accumulation and PLC activation, HEK293 cells were transfected 24 h after seeding. Cells were transfected with 45 ng plasmid DNA and 0.45 µL Metafectene (Biontex) per well in MEM without supplements. For the NanoGlo® HiBiT assay, transfection was performed as described previously elsewhere.^47^ In short, low amounts of HiBiT-tagged receptor mutants were transfected (0.45 ng/well) and carrier DNA (pGEM-3Zf(+), Promega) was added to 45 ng DNA/well in total in advanced MEM (Thermo Fisher Scientific, Gibco) to ensure comparable transfection conditions to the other performed assays. Transfection for both HiBiT-assay (cell surface and total expression) was carried out simultaneously to ensure comparability.

#### Determination of total and cell surface expression using NanoGlo® HiBiT assay

The amount of receptors expressed on the cell membrane as well as total cell expression was determined using the NanoGlo® HiBiT detection system (Promega). Assay was performed according to manufacturer’s protocol (rapid measurements) and has been described elsewhere.^47^ In short, 48 h after transfection, media were changed into Opti-MEM reduced serum media (Thermo Fisher Scientific, Gibco) without phenol red (50 µL/well) to remove background noise. Determination of cell surface expression was performed by injection of 50 µL of HiBiT extracellular substrate in the appropriate buffer supplemented with LgBiT. Total expression determination was carried out similarly, with 50 µL/well of HiBiT Lytic substrate combined with LgBiT in the appropriate buffer containing detergents to lyse the cells (Promega). After orbital shaking for 3 min at 300 cycles per minute, plates were incubated for 10 min at room temperature. Luminescence was measured using a plate reader (Mithras LB 940, Berthold Technologies). As background control, cells transfected with empty vector pcDNA3 were used and values were subtracted from the sample emissions.

#### Determination of Gs-activation by measurement of cAMP accumulation using AlphaScreen™ assay

Ligand-induced activation of MC4R was determined by using the AlphaScreen™ assay (Perkin Elmer Life Science) according to the manufacturer’s protocol and has been described elsewhere.^65^ In brief, 48 h post transfection, cells were challenged with either α-MSH or NDP-α-MSH (1 µM to 0.1 nM) in stimulation buffer (138 nM NaCl, 6 mM KCl, 1 mM MgCl_2_ ∙ 6H_2_O, 5.5 mM glucose, 20 mM HEPES, 1 mM CaCl_2_ ∙ 2H_2_O, 0.1% BSA, pH 7.4) containing 1 mM IBMX for 40 min for at 37 °C and 5% CO_2_. Incubation was stopped by freezing cells at −80 °C for 10 min prior to cAMP measurements. The determination of cAMP accumulation was performed following to the manufactures’ instructions (Perkin Elmer Life Science) and measured with a plate reader (Mithras LB 940, Berthold Technologies).

#### Determination of PLC activation via NFAT reporter gene assay

As a read-out system for G_q/11_ signaling, activation of PLC-β activation was investigated using NFAT responsive element (NFAT-luc, pGL4.33, Promega) located in the promotor region of the gene encoding a firefly luciferase and luciferase is expressed upon second messenger activation. After incubation for 48 h after transfection, cells were stimulated with NDP-α-MSH or setmelanotide (1 mM to 0.1 nM) in MEM without supplements for 6 h at 37 °C and 5% CO_2_. The stimulation was stopped by exchange of media with 1× passive lysis buffer (PLB, Promega) and freezing the cells at −80 °C for 10 min. Luciferase activity was then measured and provided information about the activation of the respective second messenger by transferring 10 µL lysate into a white opaque 96-well plate. Injection of 40 µL firefly luciferase substrate (Promega) and measurement of luminescence were performed with a plate reader (Mithras LB 940).

### Statistical analysis

Statistical analysis was performed using GraphPad Prism 6. Appropriate tests were carried out and indicated for each individual data set. Statistical significance was set at ^∗^*P* ≤ 0.05, ^∗∗^*P* ≤ 0.01, ^∗∗∗^*P* ≤ 0.001 and ^∗∗∗∗^*P* ≤ 0.0001. Concentration-response curves of each experiment were analyzed by fitting a non-linear regression model for sigmoidal response in GraphPad PRISM 6 (GraphPad Software Inc.) to determine EC_50_ values. Statistics for all functional data are given in Supplementary information, Tables S7−S9.

## Supplementary information

Supplementary figure 1

Supplementary figure 2

Supplementary figure 3

Supplementary figure 4

Supplementary figure 5

Supplementary figure 6

Supplementary figure 7

Supplementary figure 8

Supplementary figure 9

Supplementary figure 10

Supplementary figure 11

Supplementary figure 12

Supplementary figure 13

Supplementary figure 14

Supplementary figure 15

Supplementary figure 16

Supplementary figure 17

Supplementary figure 18

Supplementary figure 19

Supplementary figure S20

Supplementary figure S21

Supplementary figure S22

Supplementary figure S23

Supplementary figure S24

Supplementary table S1

Supplementary table S2

Supplementary table S3

Supplementary table S4

Supplementary table S5

Supplementary table S6

Supplementary table S7

Supplementary table S8

Supplementary table S9

Supplementary table S10

Supplementary Data S1

## Data Availability

The corresponding coordinates and cryo-EM density maps for the NDP-α-MSH–MC4R–Gsαβγ-Nb35 complex and the Setmelanotide–MC4R–Gsαβγ–Nb35 complex have been deposited in the Protein Data Bank (http://www.rcsb.org/pdb) with the entry codes 7PIV and 7PIU, and in EMDB (http://www.ebi.ac.uk/pdbe/emdb/) with the entry codes EMD-13454 and EMD-13453, respectively. References for supplementary figures and tables are listed in Supplementary information, Data S1.
