# Supplementary material for: Structures of active melanocortin-4 receptor–Gs-protein complexes with NDP-α-MSH and setmelanotide
Source: Cell Res. 2021 Sep 24;31(11):1176–89. doi: 10.1038/s41422-021-00569-8 (PMC8563958; doi:10.1038/s41422-021-00569-8)
Supplement: Supplementary file 1 — Supplementary figure 1 [file 41422_2021_569_MOESM1_ESM.pdf]

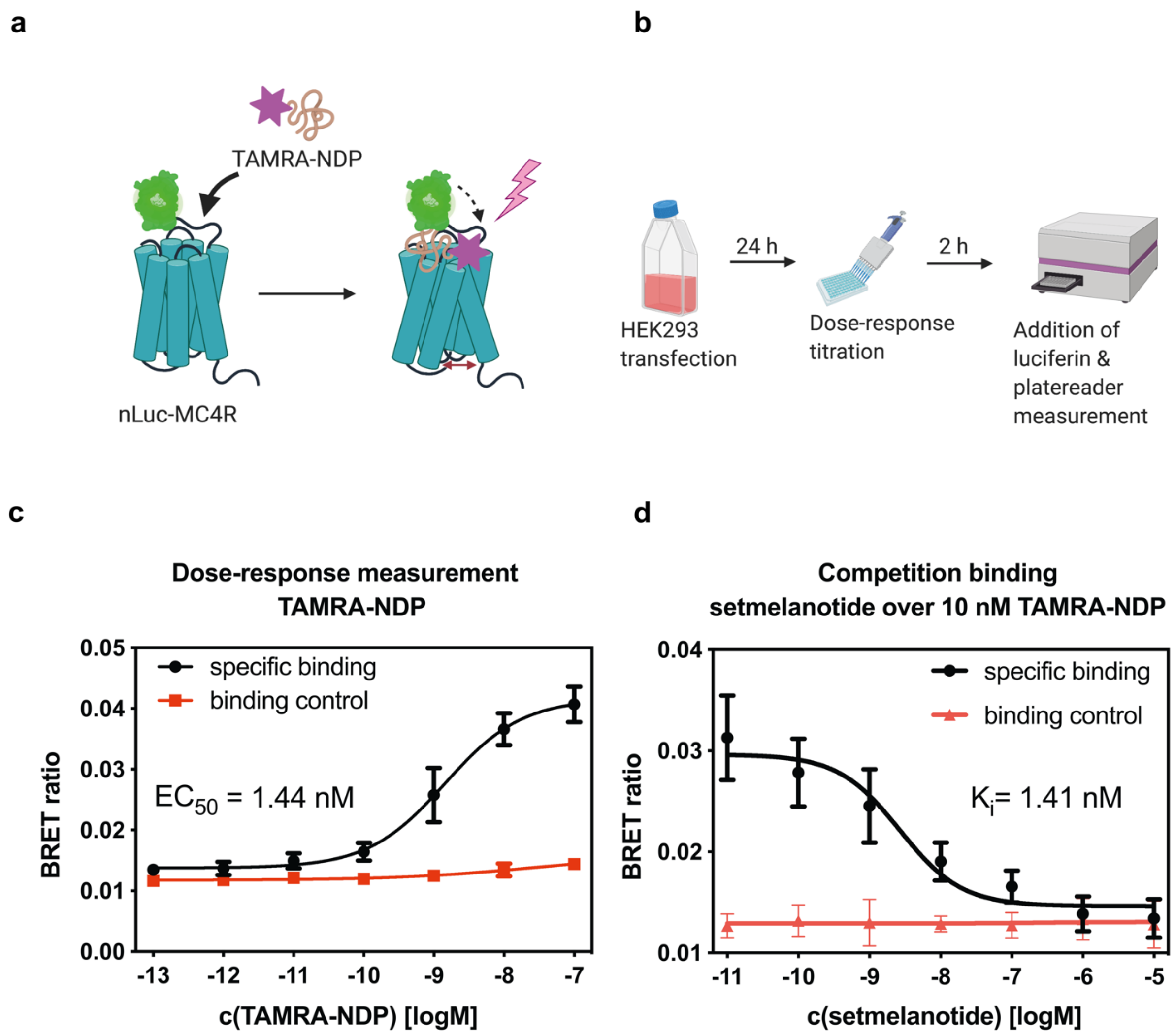

Fig. S1: ***Experimental set-up of NanoLuc^{TM} Luciferase assay and ligand binding data.***
(**a**) Schematic representation of NanoLuc^{TM} Luciferase (nLuc) based MC4R ligand binding assay. nLuc protein was fused at the receptor N-terminus. Bioluminescence resonance energy transfer (BRET) is observed in dependence of the relative distance of the fluorescent (with the fluorophore 5-carboxytetramethylrhodamine (TAMRA)) labelled NDP-α-MSH (TAMRA-NDP) and nLuc-MC4R.
(**b**) Ligand binding assay workflow, HEK293T cells were infected 24 h prior, followed by media exchange against ligand titrations. Ligand binding equilibration is ensured by 2 h incubation time with subsequent addition of the nLuc substrate luciferin and measurement of the short-pass filter (460 nm) and long-pass filter (610 nm) using a fluorescent plate reader. The BRET ratio is the quotient of long-pass by short-pass.
(**c**) Titration of TAMRA-NDP from 10 µM to 0.1 pM is plotted as dose-response measurement with an EC_{50} of 1.44 nM.
(**d**) Setmelanotide binding was determined by competing the agonist setmelanotide against 10 nM TAMRA-NDP with a resulting K_i of 1.41 nM. The addition of 20 µM non-fluorescent labeled NDP-α-MSH enhanced the BRET effect induced by non-specific binding (binding control).
(**a**, **b**) was created with *Biorender.com*.
