# Supplementary material for: Structures of active melanocortin-4 receptor–Gs-protein complexes with NDP-α-MSH and setmelanotide
Source: Cell Res. 2021 Sep 24;31(11):1176–89. doi: 10.1038/s41422-021-00569-8 (PMC8563958; doi:10.1038/s41422-021-00569-8)
Supplement: Supplementary file 2 — Supplementary figure 2 [file 41422_2021_569_MOESM2_ESM.pdf]

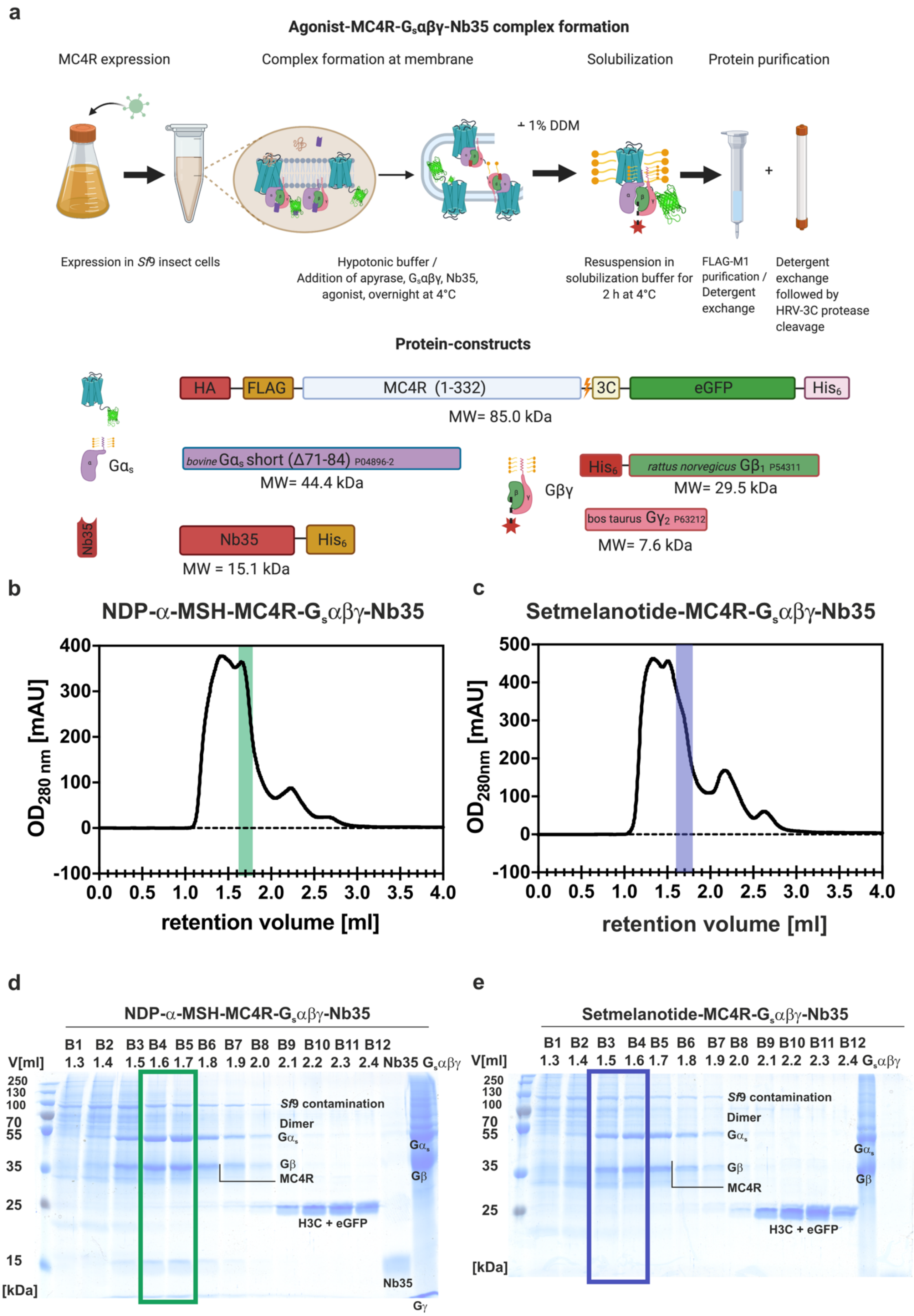
a
Agonist-MC4R-GsαβУ-Nb35 complex formation
MC4R expression
Complex formation at membrane
Solubilization
Protein purification
+ 1% DDM
Expression in Sf9 insect cells
Hypotonic buffer / Addition of apyrase, Gsαβγ, Nb35, agonist, overnight at 4°C
Resuspension in solubilization buffer for 2 h at 4°C
FLAG-M1 purification / Detergent exchange
Detergent exchange followed by HRV-3C protease cleavage
Protein-constructs
HA
FLAG
MC4R (1-332)
3C
eGFP
His6
MW= 85.0 kDa
Gαs
bovine Gαs short (Δ71-84) P04896-2
MW= 44.4 kDa
His6
rattus norvegicus Gβ1 P54311
MW= 29.5 kDa
Gβγ
bos taurus Gγ2 P63212
MW= 7.6 kDa
Nb35
His6
MW = 15.1 kDa
b
NDP-α-MSH-MC4R-Gsαβγ-Nb35
OD280 nm [mAU]
retention volume [ml]
c
Setmelanotide-MC4R-Gsαβγ-Nb35
OD280nm [mAU]
retention volume [ml]
d
NDP-α-MSH-MC4R-Gsαβγ-Nb35
B1 B2 B3 B4 B5 B6 B7 B8 B9 B10 B11 B12
V[ml] 1.3 1.4 1.5 1.6 1.7 1.8 1.9 2.0 2.1 2.2 2.3 2.4 Nb35 Gsαβγ
Sf9 contamination
Dimer
Gαs
Gβ
MC4R
H3C + eGFP
Nb35
Gγ
[kDa]
e
Setmelanotide-MC4R-Gsαβγ-Nb35
B1 B2 B3 B4 B5 B6 B7 B8 B9 B10 B11 B12
V[ml] 1.3 1.4 1.5 1.6 1.7 1.8 1.9 2.0 2.1 2.2 2.3 2.4 Gsαβγ
Sf9 contamination
Dimer
Gαs
Gβ
MC4R
H3C + eGFP
[kDa]

Fig. S2: ***Workflow and biochemistry data.***

(**a**) Workflow for the assembly of the MC4R−$G_s\alpha\beta\gamma$ complexes stabilized by NDP-α-MSH or setmelanotide. MC4R−eGFP and $G_s\alpha\beta\gamma$ (Gs) were expressed in *Sf*9 and *Tn*i cells, respectively. Gs was purified and added in excess to MC4R−eGFP expressing *Sf*9 cells resuspended in a hypotonic buffer, containing apyrase, Nanobody 35 (Nb35) and 1 µM of agonists (NDP-α-MSH or setmelanotide). After overnight incubation membranes were resuspended in a buffer containing 1 % DDM and 0.1 % CHS. Followed by a FLAG M1 antibody purification. During the washing steps, DDM was exchanged against 0.01 % LMNG and 0.001 % CHS. After elution HRV-3C protease was added and the His_6 tag at Gβ and eGFP at MC4R was cleaved overnight. The agonist−MC4R−Gs−Nb35 complexes were separated from HRV-3C protease and remaining eGFP by size-exclusion chromatography using a Superdex 200 Increase 5/150 GL column for the complexes with

(**b**) NDP-α-MSH and

(**c**) setmelanotide. Subsequent SDS gel chromatography of

(**d**) NDP-α-MSH−MC4R−Gs−Nb35 and

(**e**) setmelanotide−MC4R−Gs−Nb35 were applied to confirm the stoichiometric ratio of MC4R and Gs. Gs-protein and Nb35 were used as controls in the last two lanes. For both complexes fractions B4 and B5 were concentrated and directly vitrified. Fig. S2a was created with *Biorender.com*.
