# Supplementary material for: Structures of active melanocortin-4 receptor–Gs-protein complexes with NDP-α-MSH and setmelanotide
Source: Cell Res. 2021 Sep 24;31(11):1176–89. doi: 10.1038/s41422-021-00569-8 (PMC8563958; doi:10.1038/s41422-021-00569-8)
Supplement: Supplementary file 3 — Supplementary figure 3 [file 41422_2021_569_MOESM3_ESM.pdf]

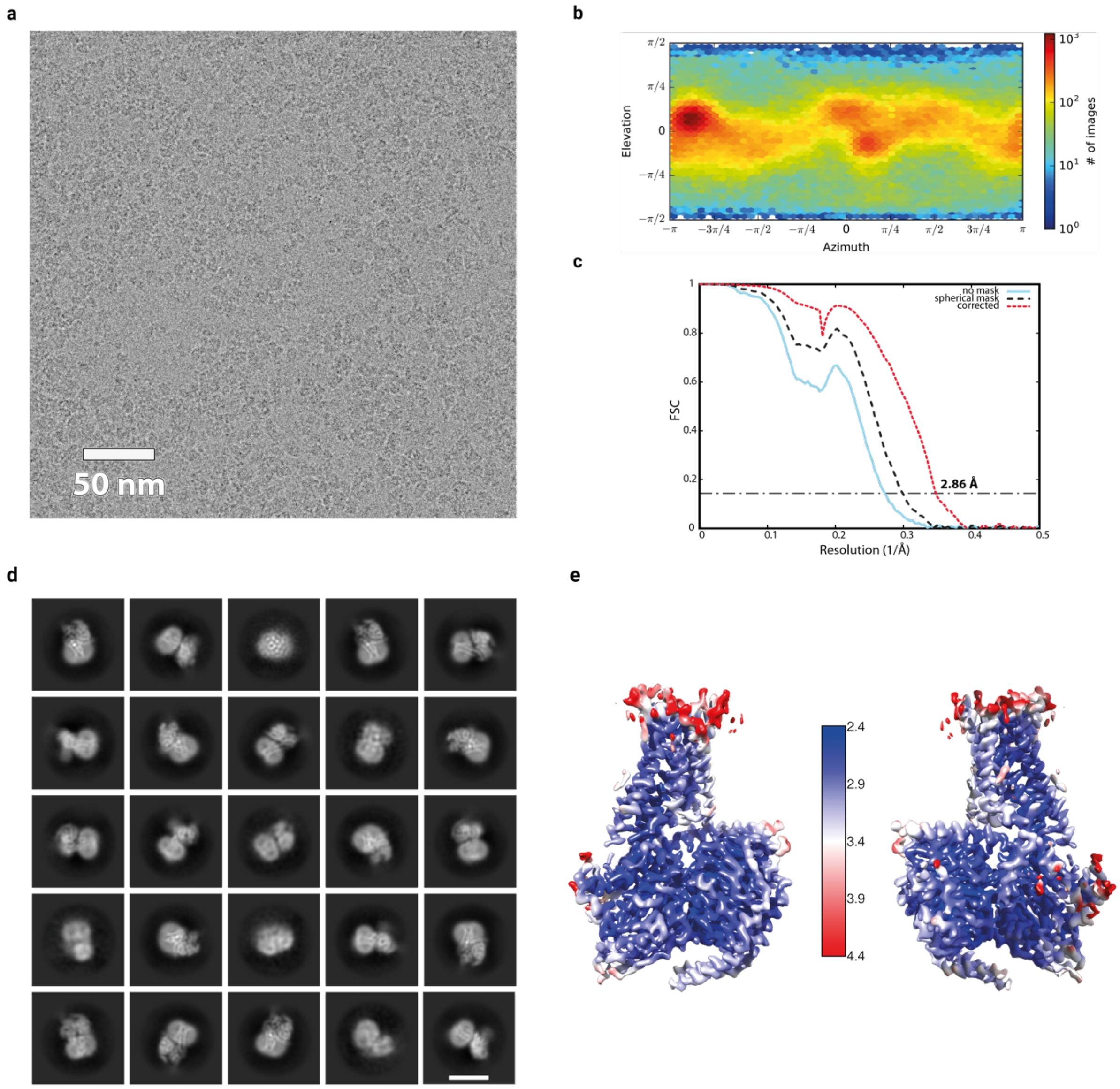

Fig. S3: ***Cryo-EM data analysis of the active NDP-α-MSH−MC4R−Gs−Nb35 complex***.

(**a**) Representative cryo-electron micrograph. The scale bar corresponds to 50 nm in the image.

(**b**) Distribution of projection directions as estimated during homogeneous refinement with program cryoSPARC.

(**c**) Global resolution estimation by Fourier shell correlation calculations (FSC = 0.143 cutoff) after "gold standard" refinement. The light blue curve was calculated without masking, the black curve by applying a spherical mask and the red one after phase randomization using a soft-mask.

(**d**) Representative 2D class averages confirm random distribution of projection directions. The scale bar corresponds to 10 nm in the image.

(**e**) Representation of local resolution estimation determined with cryoSPARC. The final cryo-EM density is colored according to the local resolution ranging from dark blue (2.4 Å) to red (4.4 Å).
