# Supplementary material for: Structures of active melanocortin-4 receptor–Gs-protein complexes with NDP-α-MSH and setmelanotide
Source: Cell Res. 2021 Sep 24;31(11):1176–89. doi: 10.1038/s41422-021-00569-8 (PMC8563958; doi:10.1038/s41422-021-00569-8)
Supplement: Supplementary file 4 — Supplementary figure 4 [file 41422_2021_569_MOESM4_ESM.pdf]

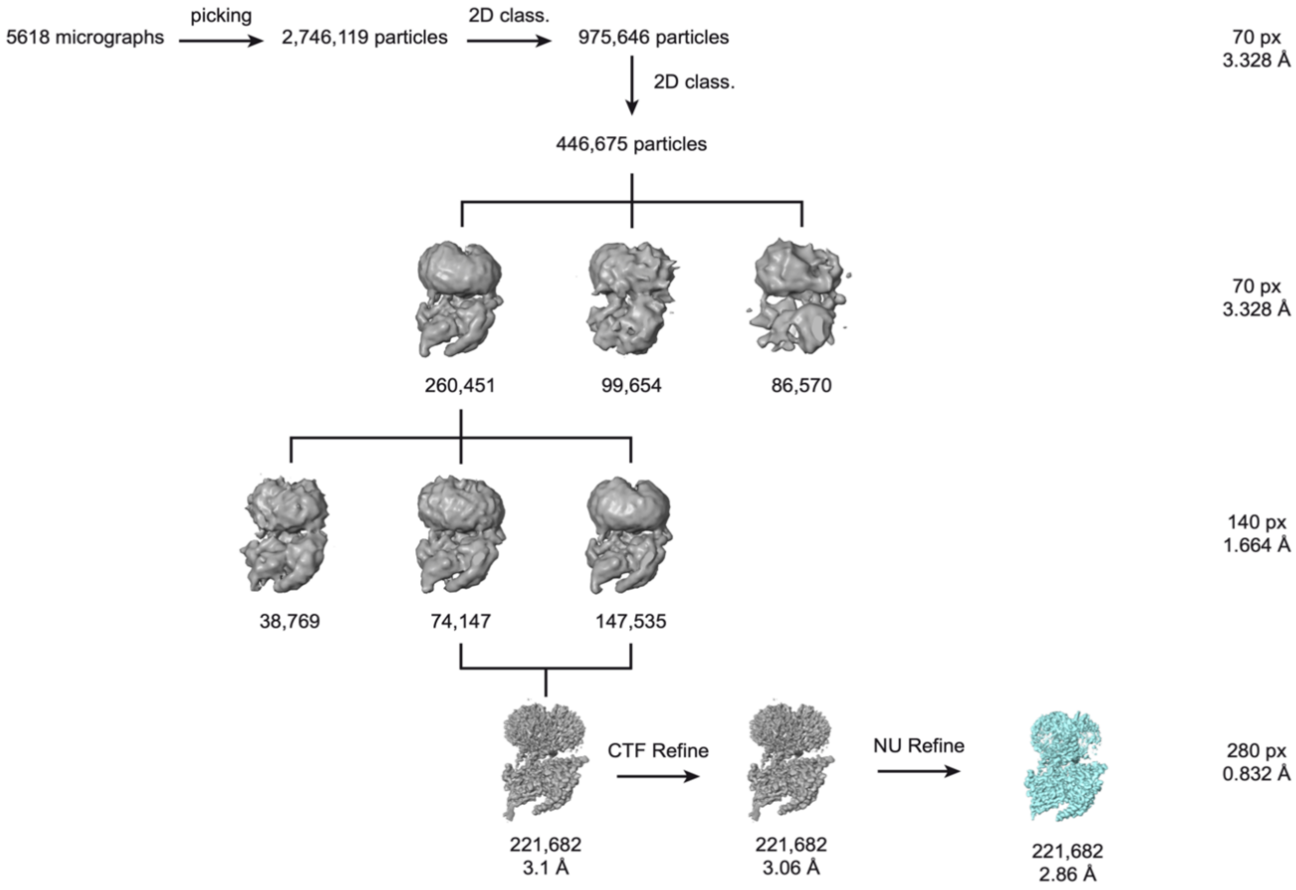

Fig. S4: ***Cryo-EM refinement sorting scheme of the active NDP-α-MSH−MC4R−Gs−Nb35 complex dataset.*** After template-based particle picking, 2,746,119 particle images were extracted and Fourier-cropped to a box size of 70 px (pixel size 3.328 Å). After two subsequent runs of reference-free 2D classifications, 446,675 particles were selected and subjected to heterogeneous 3D refinement yielding 260,451 particle images. Re-extraction of particle images Fourier-cropped to 140 px box size (1.664 Å pixel size) was followed by another round of heterogeneous refinement. The reconstruction of two classes was virtually identical by visual inspection, therefore particle images were combined and re-extracted unbinned with a box size of 280 px (0.832 Å/px). Homogeneous refinement generated a reconstruction with a resolution of 3.1 Å, which was improved to 2.86 Å by CTF refinement followed by non-uniform (NU) refinement.
