# Supplementary material for: Structures of active melanocortin-4 receptor–Gs-protein complexes with NDP-α-MSH and setmelanotide
Source: Cell Res. 2021 Sep 24;31(11):1176–89. doi: 10.1038/s41422-021-00569-8 (PMC8563958; doi:10.1038/s41422-021-00569-8)
Supplement: Supplementary file 6 — Supplementary figure 6 [file 41422_2021_569_MOESM6_ESM.pdf]

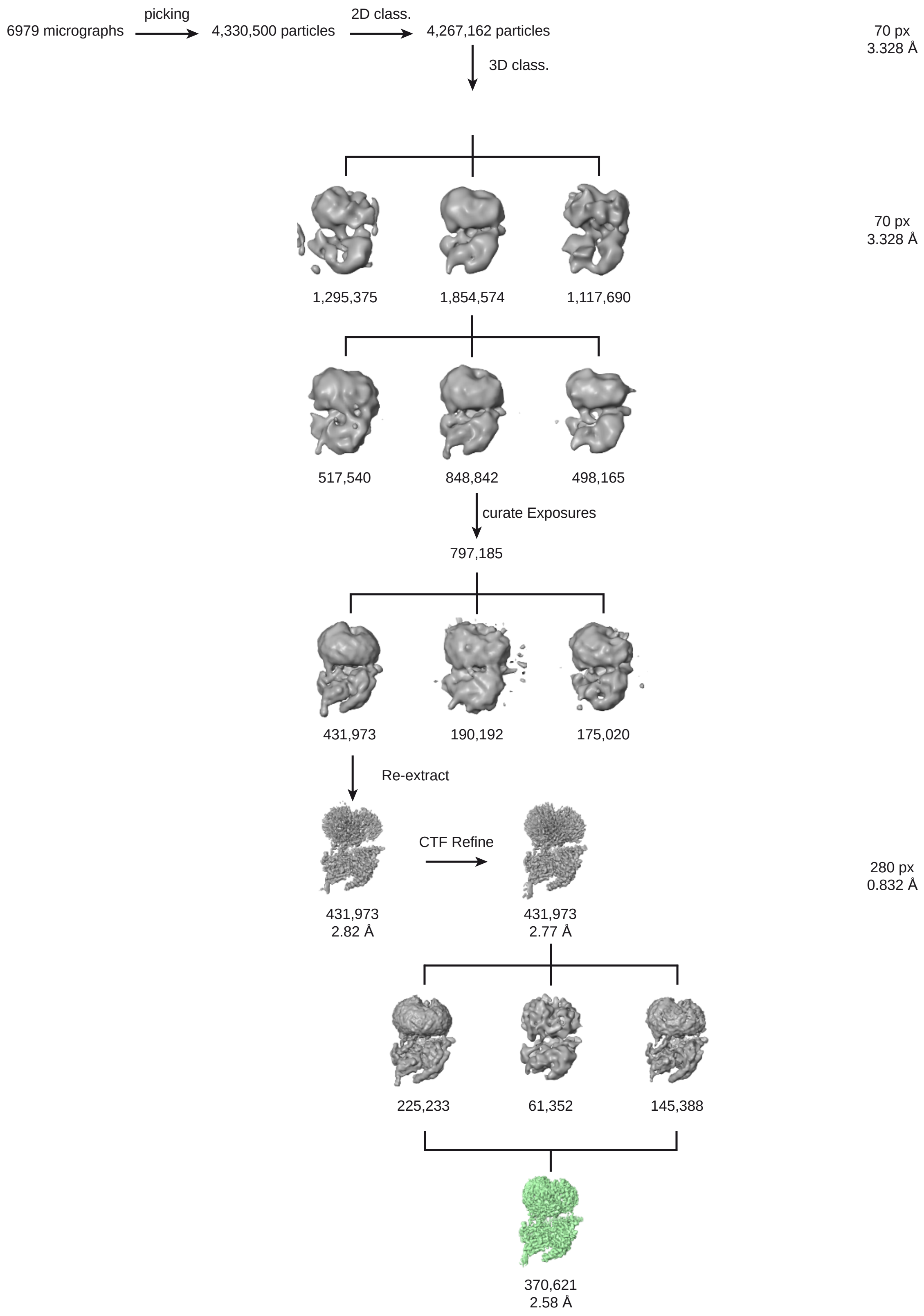

Fig. S6: ***Cryo-EM refinement sorting scheme of the active setmelanotide−MC4R−Gs−Nb35 complex dataset.*** Initially, 4,330,500 particles were picked and extracted by Fourier-cropping with a box size of 70 px (3.328 Å/px). 2D classification was followed by two iterative 3D classifications using the NDP-α-MSH−MC4R−Gs−Nb35 filtered to 30 Å as template yielding

848,842 particle images. Micrographs were curated omitting resolutions above 4 Å and high local motion, leaving 797,185 particles for another iteration of heterogeneous refinement. 431,973 particles were re-extracted at full resolution (0.832 Å/px) and subjected to homogeneous refinement. The resulting 2.82 Å reconstruction was improved by CTF refinement to 2.77 Å. Another heterogeneous refinement was conducted to select 370,621 particle images for a final NU refinement, yielding a 2.58 Å reconstruction of the setmelanotide−MC4R−Gs−Nb35 complex.
