# Supplementary material for: Structures of active melanocortin-4 receptor–Gs-protein complexes with NDP-α-MSH and setmelanotide
Source: Cell Res. 2021 Sep 24;31(11):1176–89. doi: 10.1038/s41422-021-00569-8 (PMC8563958; doi:10.1038/s41422-021-00569-8)
Supplement: Supplementary file 8 — Supplementary figure 8 [file 41422_2021_569_MOESM8_ESM.pdf]

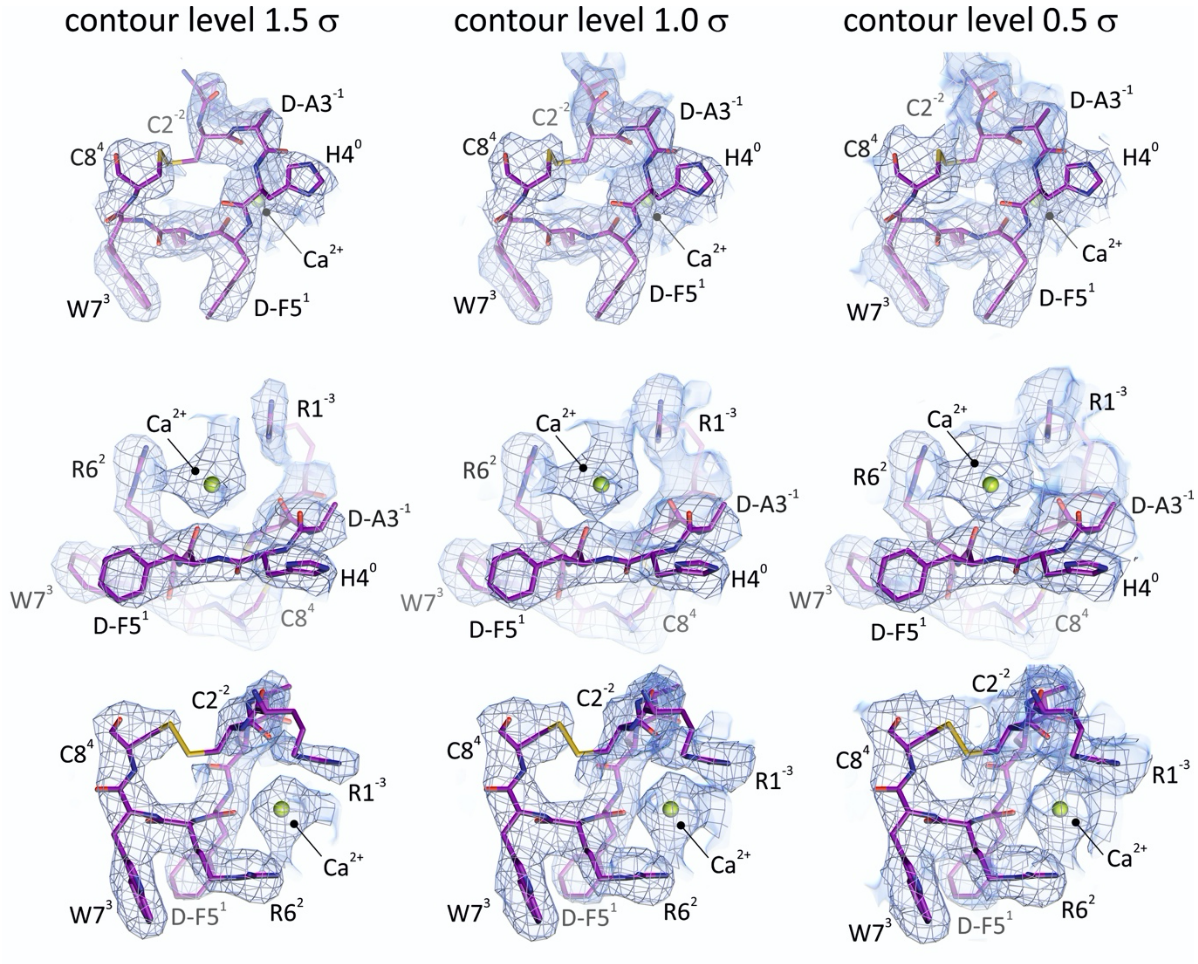

Fig. S8: ***Quality of the cryo-EM density map of setmelanotide in the setmelanotide−MC4R−Gs−Nb35 complex.*** Three different views (top to bottom) on the setmelanotide ligand (purple color) and the binding calcium ion (lemon green color). All figures show the cryo-EM densities of the ligand (light blue colored mesh/volume) contoured at three different contour levels (1.5 (left), 1.0 (middle) and 0.5 σ (right)). Setmelanotide is depicted as sticks and the calcium ion as sphere representation.
