# Supplementary material for: Structures of active melanocortin-4 receptor–Gs-protein complexes with NDP-α-MSH and setmelanotide
Source: Cell Res. 2021 Sep 24;31(11):1176–89. doi: 10.1038/s41422-021-00569-8 (PMC8563958; doi:10.1038/s41422-021-00569-8)
Supplement: Supplementary file 11 — Supplementary figure 11 [file 41422_2021_569_MOESM11_ESM.pdf]

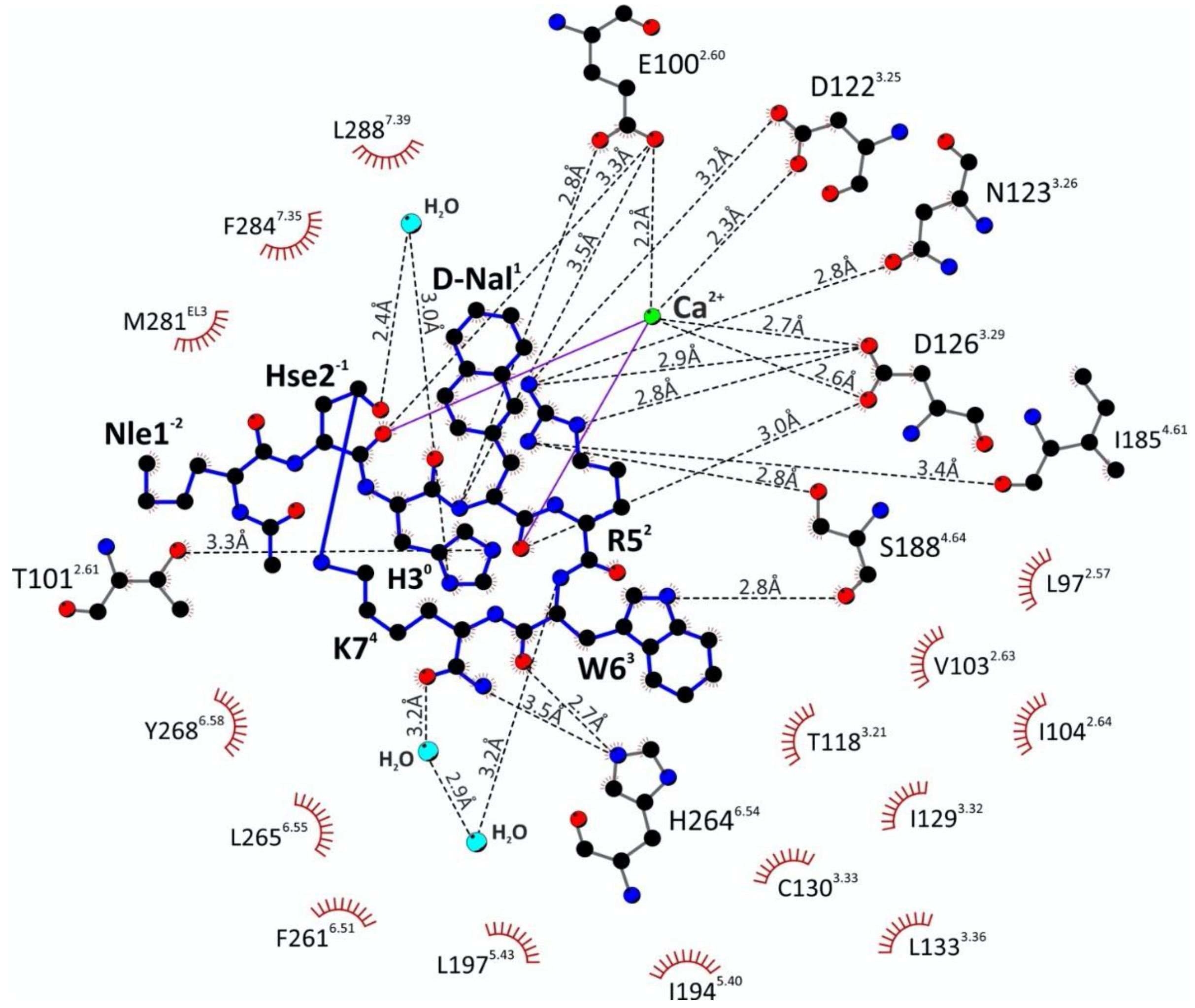

Fig. S11: ***Interactions between antagonist SHU9119 and MC4R.*** Potential hydrogen bonds and hydrophobic interactions of SHU9119 binding to MC4R (PDB ID: 6w25 [3]) were analyzed using HBPLUS [1] with a maximum donor acceptor distance of 3.5 Å as implemented in the program LigPlot+ v.2.1 [2], which was used to draw this schematic view. Potential hydrogen bonds are indicated by black dashed lines and distance labels. Residues with closest distances of less than 3.9 Å are deemed to be in van der Waals contact. These residues are shown as dashed, red segments of a circle. Water molecules (cyan color) and the calcium ion (green color) are depicted as spheres, respectively.
