# Supplementary material for: Structures of active melanocortin-4 receptor–Gs-protein complexes with NDP-α-MSH and setmelanotide
Source: Cell Res. 2021 Sep 24;31(11):1176–89. doi: 10.1038/s41422-021-00569-8 (PMC8563958; doi:10.1038/s41422-021-00569-8)
Supplement: Supplementary file 12 — Supplementary figure 12 [file 41422_2021_569_MOESM12_ESM.pdf]

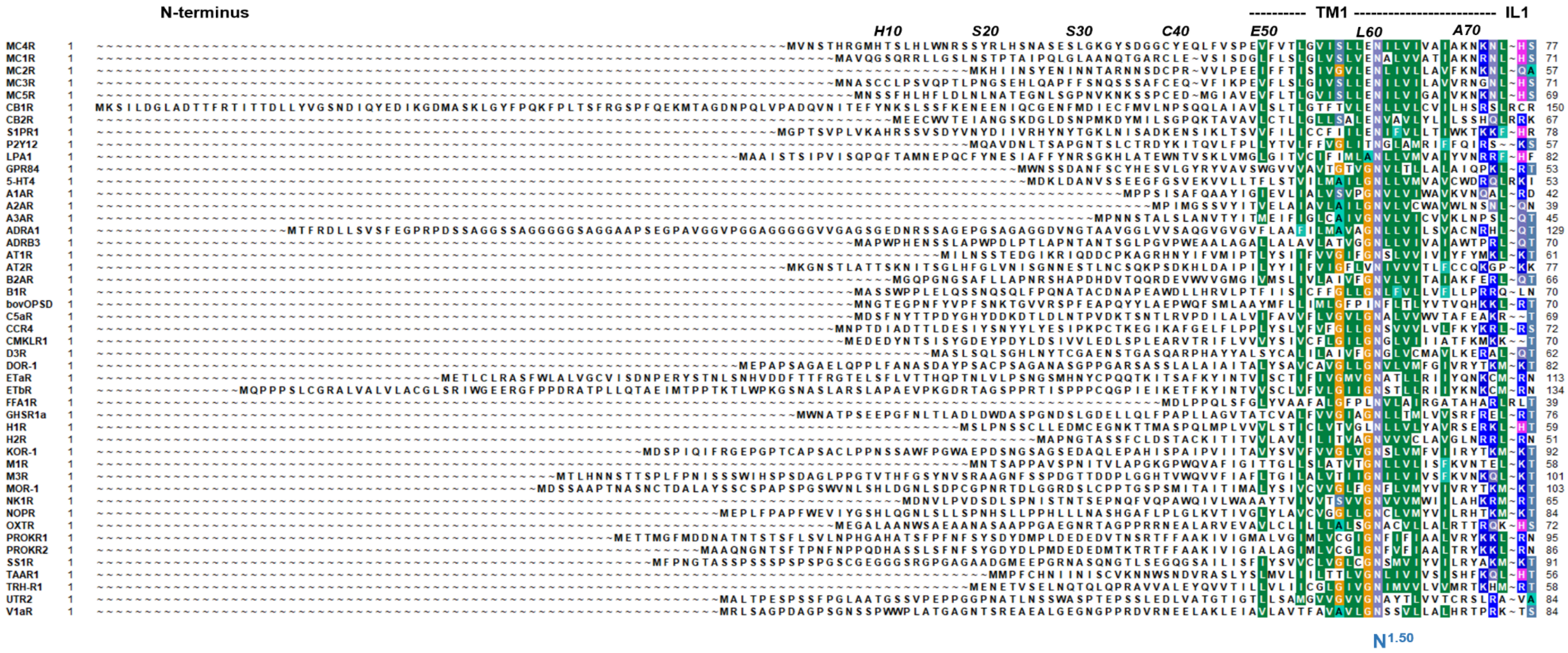

Fig. S12a-e: ***Sequence alignment of 47 human class A GPCR***. Highly conserved positions according to the unifying *Ballesteros and Weinstein* numbering scheme [4] are indicated by respective numbers below the alignment. The structural dimensions of each MC4R transmembrane helix (TM) or inter-connecting loops (IL = intracellular loop; EL = extracellular loop) are indicated above the sequences, as well as numbers for the MC4R sequence. The alignment was visualized using the software BioEdit [5]. Specific background colors indicating conservation (Blossum62 matrix) and reflecting chemical properties of the amino acid side chains: black, proline; blue, positively charged; cyan/green, aromatic and hydrophobic; green, hydrophobic; red, negatively charged; gray, hydrophilic; dark red, cysteines; and magenta, histidine.

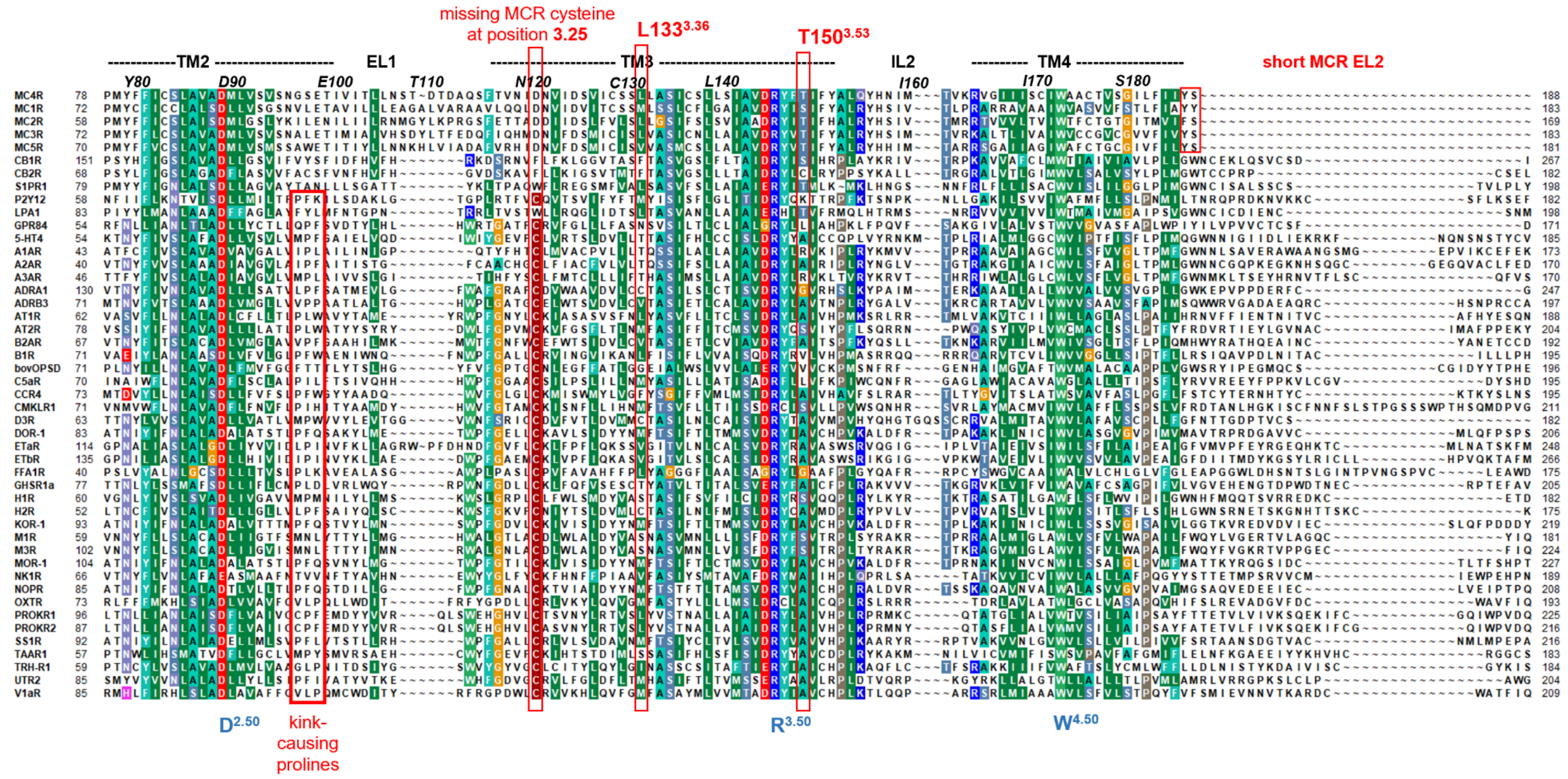
missing MCR cysteine at position 3.25
L133 3.36
T150 3.53
short MCR EL2
TM2
EL1
TM3
IL2
TM4
D2.50
kink-causing prolines
R3.50
W4.50

Fig. S12b

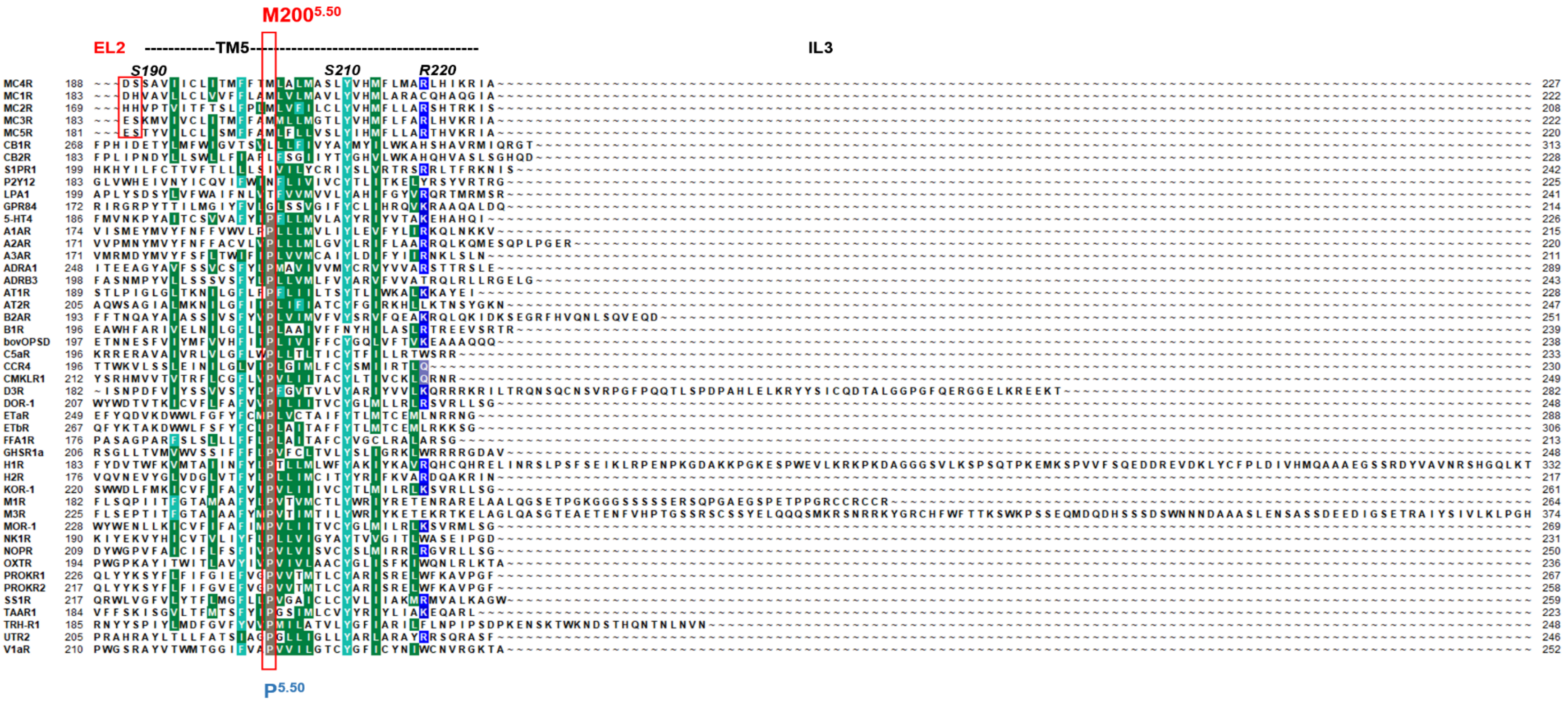

Fig. S12c

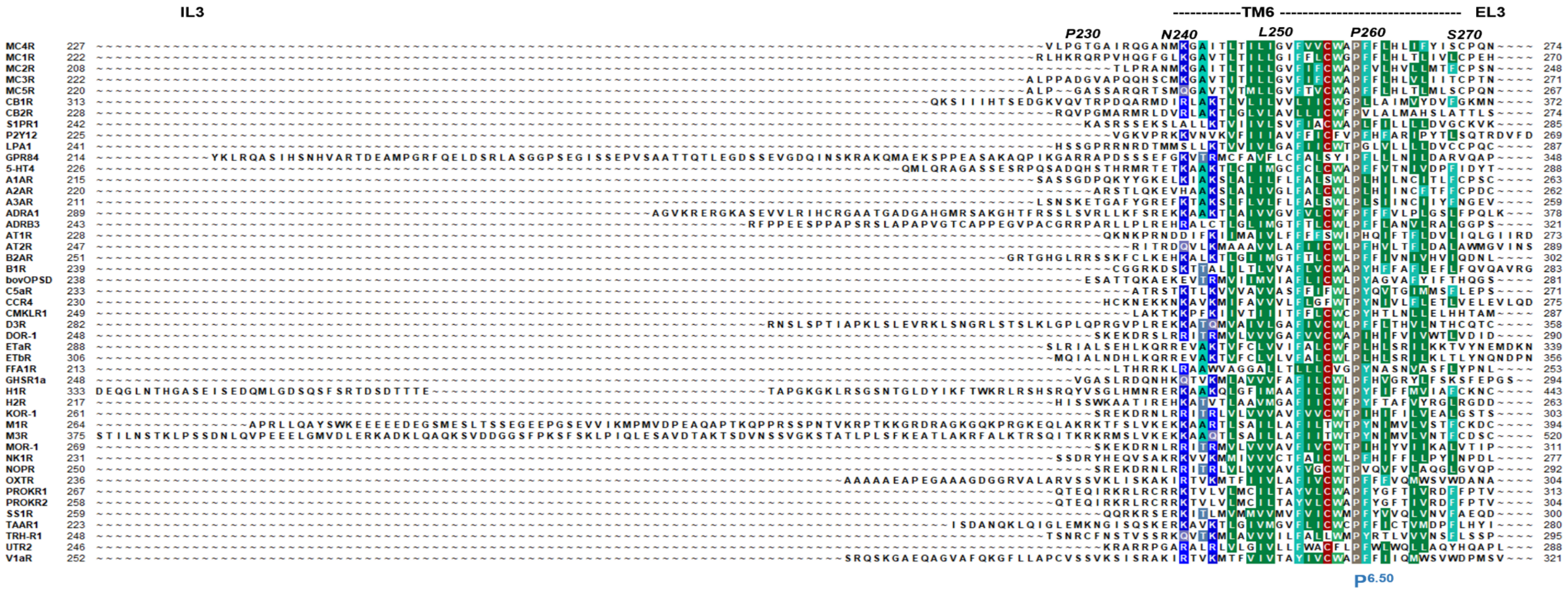

Fig. S12d

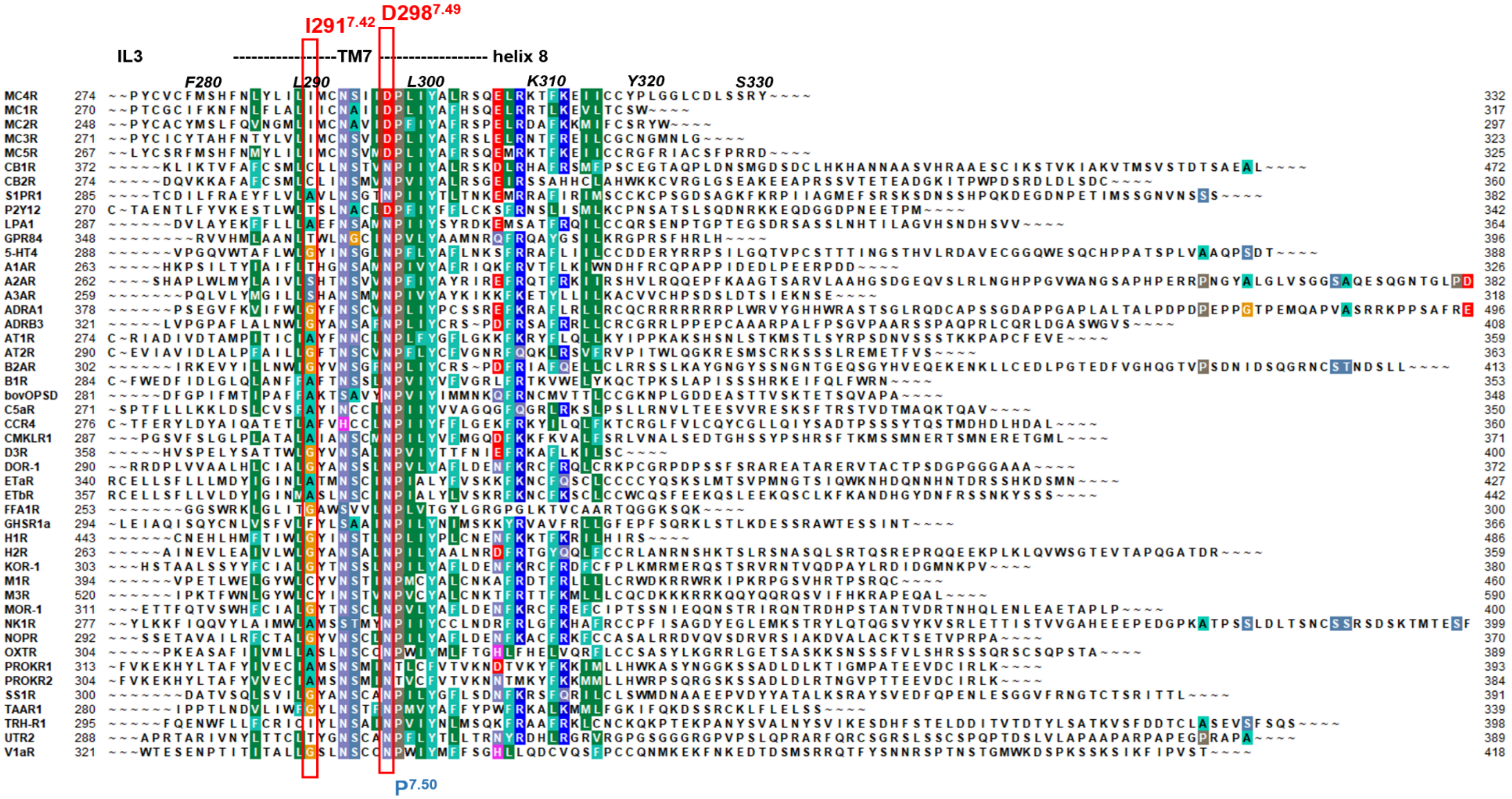
I291 7.42
D298 7.49
IL3
TM7
helix 8
F280
L290
L300
K310
Y320
S330
P7.50

Fig. S12e
