# Supplementary material for: Structures of active melanocortin-4 receptor–Gs-protein complexes with NDP-α-MSH and setmelanotide
Source: Cell Res. 2021 Sep 24;31(11):1176–89. doi: 10.1038/s41422-021-00569-8 (PMC8563958; doi:10.1038/s41422-021-00569-8)
Supplement: Supplementary file 13 — Supplementary figure 13 [file 41422_2021_569_MOESM13_ESM.pdf]

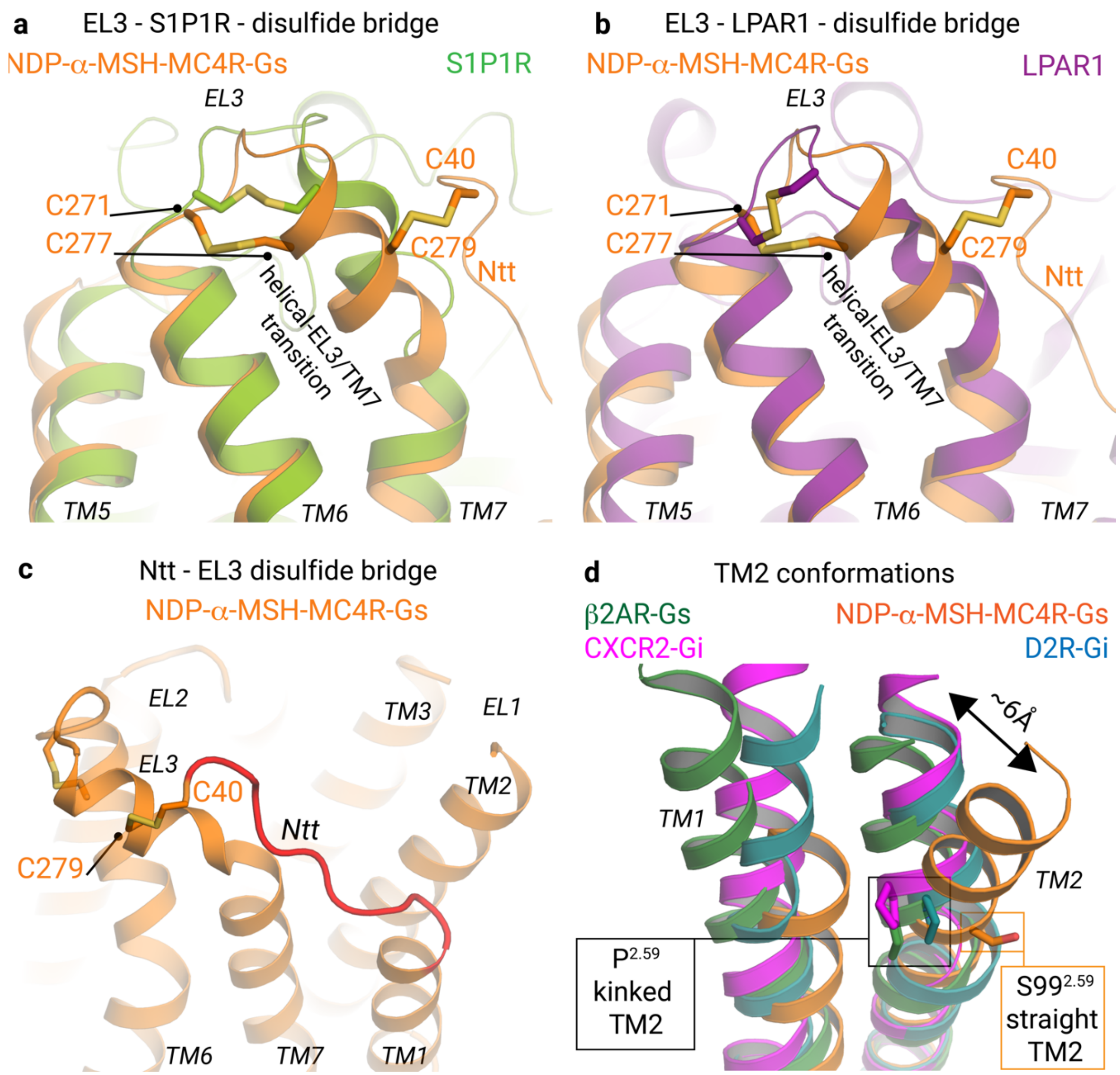

Fig. S13: ***Specificities of the MC4R in the extracellular region.***

The active state MC4R structure (orange color) shares with

(**a**) the sphingosine 1-phosphate receptor 1 (S1P1R, light green color, PDB ID: 3v2y [6]) and

(**b**) the lysophosphatidic acid receptor 1 (LPAR1, deep purple color, PDB ID: 4z34 [7] a disulfide bridge in the EL3, which is involved in forming a specific conformation including a helical transition to TM7.

(**c**) The MC4R has a second disulfide bridge between EL3 and the receptor N-terminus (Ntt) that constraints the extracellular helix close to TM7 and by this participates in forming the large peptide ligand-binding region.

(**d**) Contrary to most other class A GPCRs, MCRs have no proline in TM2 (Fig. S12b) which usually causes a kink bulge and slight rotation of TM2 toward the extracellular region. The superposition of the MC4R structure in the active state with various already determined GPCR structures (β2AR, forest green color, PDB ID: 3sn6 [8]; D2R, blue color, PDB ID: 6vms [9]; CXCR2, magenta color, PDB ID: 6lfo [10]) illustrates the difference in spatial TM2 allocation (5.9 Å distance measured between position 2.65 in MC4R and β2AR).
