# Supplementary material for: Structures of active melanocortin-4 receptor–Gs-protein complexes with NDP-α-MSH and setmelanotide
Source: Cell Res. 2021 Sep 24;31(11):1176–89. doi: 10.1038/s41422-021-00569-8 (PMC8563958; doi:10.1038/s41422-021-00569-8)
Supplement: Supplementary file 14 — Supplementary figure 14 [file 41422_2021_569_MOESM14_ESM.pdf]

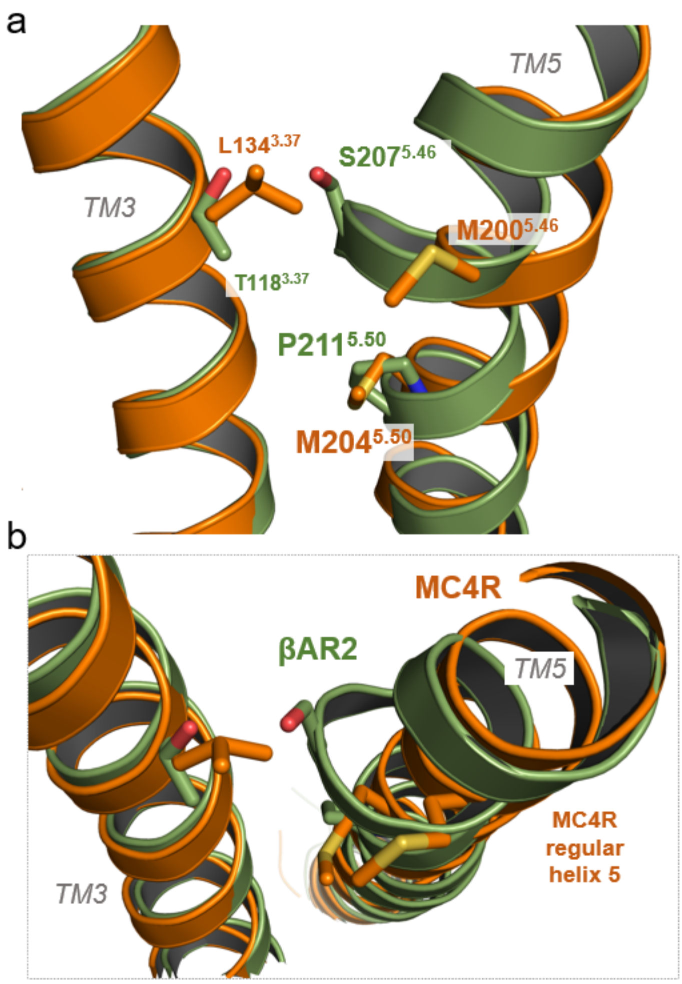

Fig. S14: ***Straight MC4R helix 5 conformation without a proline induced kink and bulge.***
(**a**) Comparison between MC4R (orange) and the β2AR (green, PDB ID: 3sn6 [8]) structures reveal differences in the TM5 helix conformation. Caused by a highly conserved proline in class A GPCRs (Fig. S12c) TM5 is usually kinked as observed also in the β2AR. In consequence, amino acids at the proline induced kink-bulge pointing to the ligand binding region and forming a specific interface with TM3. S207$^{5.46}$ in the bulge of the β2AR−TM5 is known to interact with ligands and is important for agonistic versus antagonistic effects. This structural helix-specificity is different in MC4R because of a methionine at position 5.50 instead of a proline. The helix is, therefore, without any bulge or kink. This contributes to forming a more distant interface to TM3,
(**b**) but also corresponding amino acids are located at spatially distinct positions compared to other class A GPCRs, e.g. position 5.46 (**a**).
