# Supplementary material for: Structures of active melanocortin-4 receptor–Gs-protein complexes with NDP-α-MSH and setmelanotide
Source: Cell Res. 2021 Sep 24;31(11):1176–89. doi: 10.1038/s41422-021-00569-8 (PMC8563958; doi:10.1038/s41422-021-00569-8)
Supplement: Supplementary file 15 — Supplementary figure 15 [file 41422_2021_569_MOESM15_ESM.pdf]

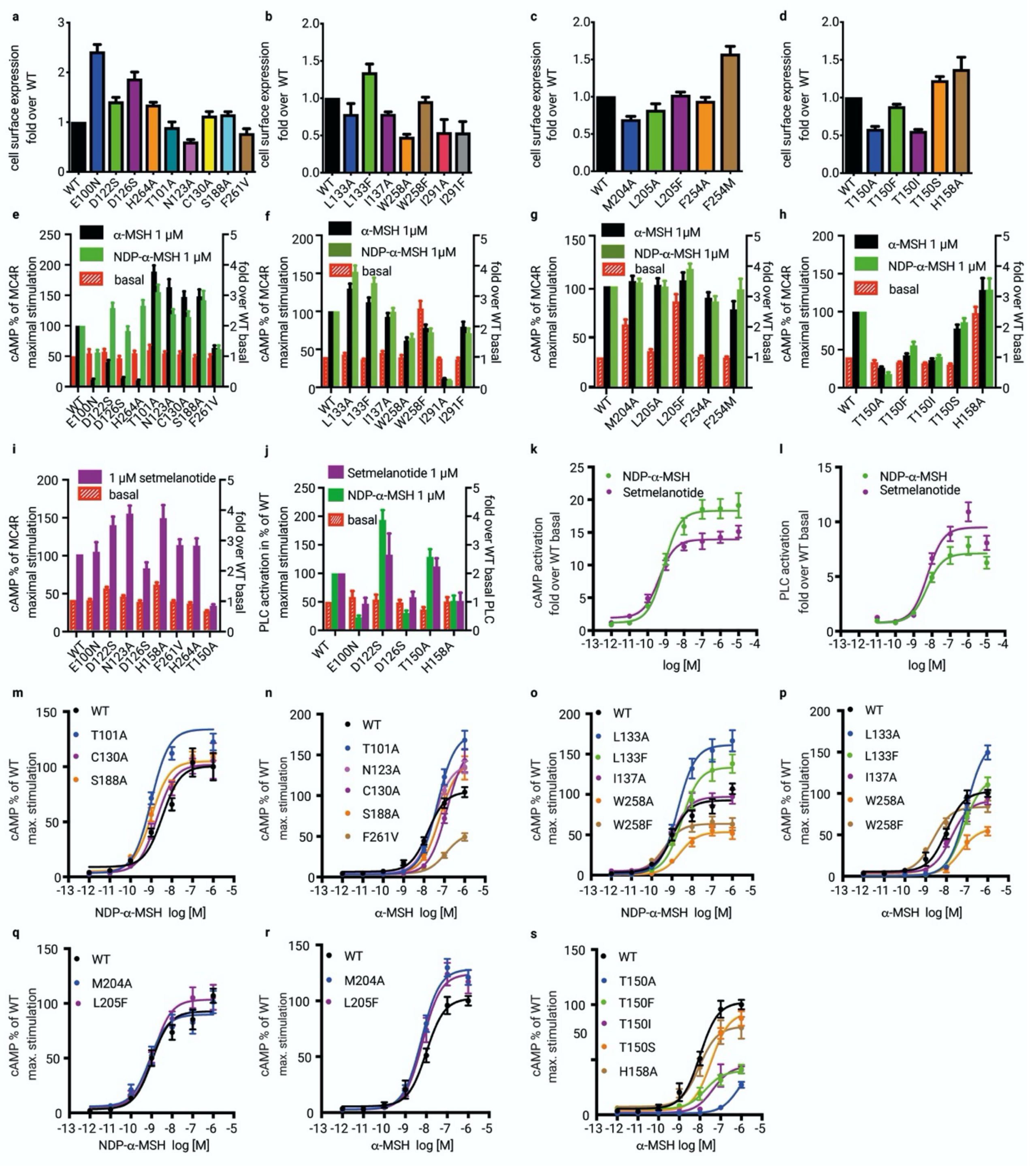

Fig. S15: ***Functional data of MC4R variants.***

(**a-d**) Cell surface expression of wild-type MC4R (WT) and mutants shown as fold over WT.

(**e-h**) Maximal Gs-protein signaling determined as cAMP accumulation of MC4R WT and mutants after addition of 1 µM α-MSH or NDP-α-MSH indicated as fold of MC4R WT max. signaling [%] (left y-axis). Basal cAMP accumulation of MC4R WT and mutants are depicted as fold over WT basal (right y-axis).

(**i**) Maximal Gs-protein signaling determined as cAMP accumulation of MC4R WT and mutants after addition of 1 µM setmelanotide indicated as fold of MC4R WT max. signaling [%] (left y-axis). Basal cAMP accumulation of MC4R WT and mutants are depicted as fold over WT basal (right y-axis).

(**j**) Maximal Gq/11-protein signaling determined by NFAT reporter gene assay (downstream transducer of PLC) of MC4R WT and mutants after addition of 1 µM setmelanotide or NDP-α-MSH indicated as fold of MC4R WT max. signaling [%] (left y-axis). Basal PLC activation of MC4R WT and mutants are depicted as fold over WT basal (right y-axis).

(**k-l**) Concentration-response curves of (**k**) Gs-protein signaling determined as cAMP accumulation and (**l**) Gq/11 signaling determined by the activation of PLC of MC4R WT upon setmelanotide and NDP-α-MSH stimulation. Data is plotted as fold over WT basal.

(**m-s**) Concentration-response curves of Gs-protein signaling determined as cAMP accumulation of WT and indicated mutants after addition of α-MSH and NDP-α-MSH shown as fold of MC4R WT max. signaling [%]. Cell surface expression data, E_{MAX} and EC_{50} values are summarized in Tables S7-S9.
