# Supplementary material for: Structures of active melanocortin-4 receptor–Gs-protein complexes with NDP-α-MSH and setmelanotide
Source: Cell Res. 2021 Sep 24;31(11):1176–89. doi: 10.1038/s41422-021-00569-8 (PMC8563958; doi:10.1038/s41422-021-00569-8)
Supplement: Supplementary file 16 — Supplementary figure 16 [file 41422_2021_569_MOESM16_ESM.pdf]

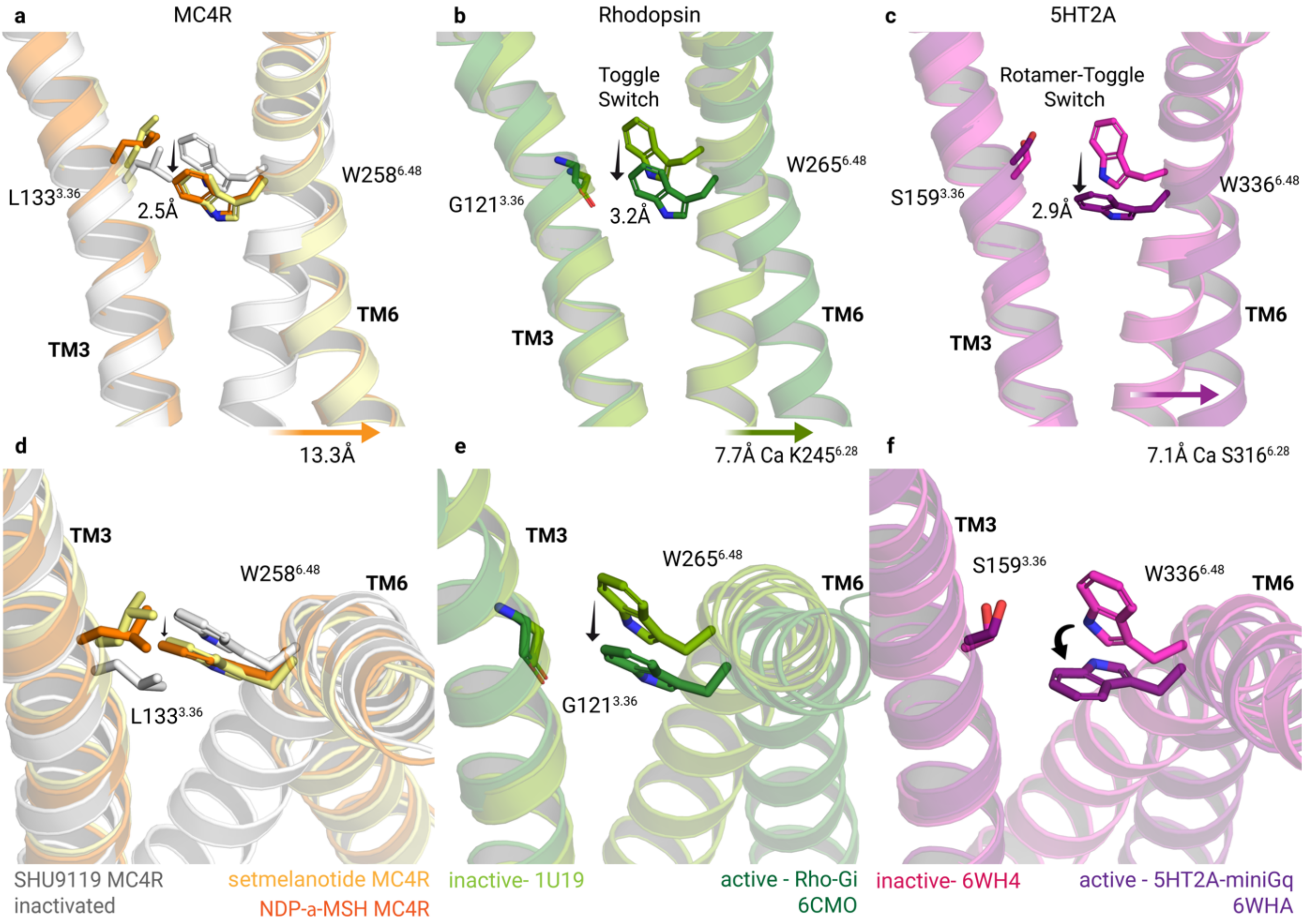

Fig. S16: ***Localization of $W^{6.48}$ in different active states of various class A GPCRs.***
Visualization of distance differences between inactive and active state conformations of $W^{6.48}$ in

(**a**) MC4R,

(**b**) bovine rhodopsin (PDB IDs: inactive- 1u19 [11], active- 6cmo [12]), and

(**c**) 5-HT_{2A}-receptor (5HT2A) (PDB IDs: inactive- 6wh4, active- 6wha [13]). The distances are associated with receptor activation (toggle switch model), thereby the tryptophan undergoes a vertical-lateral shift (measured at NE1 of Trp). In the 5HT2A structure the tryptophan side chain undergoes additional strong rotation. The associated TM6 outward movements are measured at Cα positions of intracellularly located (**a**) $M241^{6.31}$, (**b**) $K245^{6.28}$, (**c**) $S316^{6.28}$, and reflects the largest spatial difference of TM6 between respective active states.

(**d-f**) Visualization of the horizontal $W^{6.48}$ shift from the top-view. The amino acid position 3.36 at TM3 in opposite to position 6.48 is different between all three receptors. However, in MC4R the $L133^{3.36}$ with a large hydrophobic side chain has a direct impact on $W^{6.48}$. In the SHU9119 antagonized MC4R structure [3] this leucine inhibits an activation related shift of TM6 at position $W^{6.48}$.
