# Supplementary material for: Structures of active melanocortin-4 receptor–Gs-protein complexes with NDP-α-MSH and setmelanotide
Source: Cell Res. 2021 Sep 24;31(11):1176–89. doi: 10.1038/s41422-021-00569-8 (PMC8563958; doi:10.1038/s41422-021-00569-8)
Supplement: Supplementary file 17 — Supplementary figure 17 [file 41422_2021_569_MOESM17_ESM.pdf]

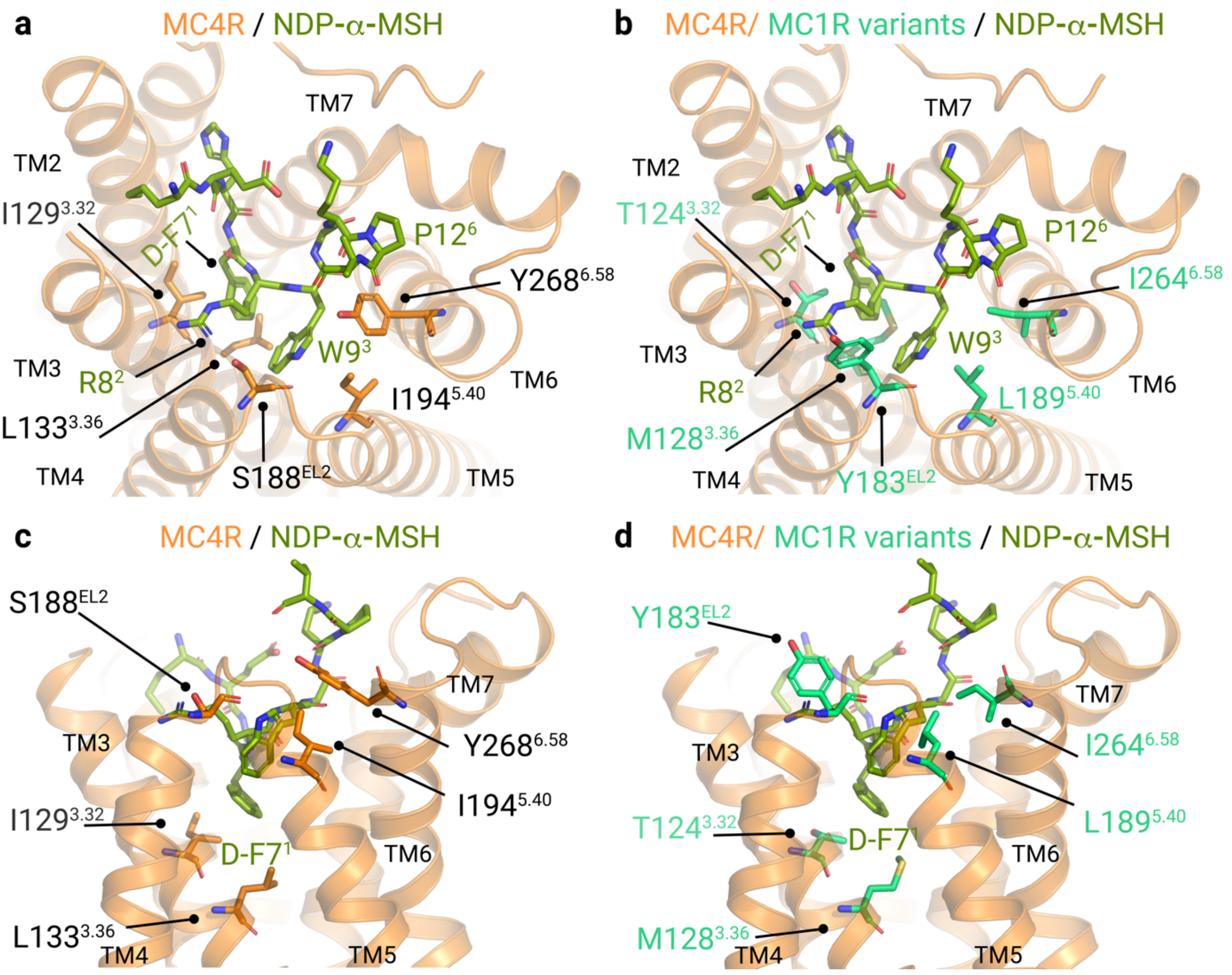

Fig. S17: ***MC1R amino acid differences in the extended ligand binding pocket based on the NDP-α-MSH−MC4R−Gs−Nb35 complex structure.*** Variations of MC1R compared to MC4R forming the NDP-α-MSH binding site were *in silico* substituted at the NDP-α-MSH−MC4R−Gs−Nb35 complex structure.

Ribbon representations, top-view (**a**-**b**) and side view (**c**-**d**) of the ligand binding pocket of NDP-α-MSH (green color) bound to MC4R (orange color) with stick representation of amino acid residues I129$^{3.32}$, L133$^{3.36}$, S188^{EL2}, I194$^{5.40}$ and Y268$^{6.58}$, which are different between MC4R and MC1R.

In (**b**,**d**) aforementioned residues were substituted with the corresponding residues at MC1R (green/cyan color), namely, T124$^{3.32}$, M128$^{3.36}$, Y183^{EL2}, L189$^{5.40}$ and I264$^{6.58}$. Of note, S188^{EL2} in MC4R EL2 corresponds to Y183^{EL2} in MC1R. The backbone carbonyl group of MC4R S188^{EL2} interacts with the W9^{3} and the side chain hydroxyl group of S188^{EL2} with R8^{2} of the agonist NDP-α-MSH.
