# Supplementary material for: Structures of active melanocortin-4 receptor–Gs-protein complexes with NDP-α-MSH and setmelanotide
Source: Cell Res. 2021 Sep 24;31(11):1176–89. doi: 10.1038/s41422-021-00569-8 (PMC8563958; doi:10.1038/s41422-021-00569-8)
Supplement: Supplementary file 18 — Supplementary figure 18 [file 41422_2021_569_MOESM18_ESM.pdf]

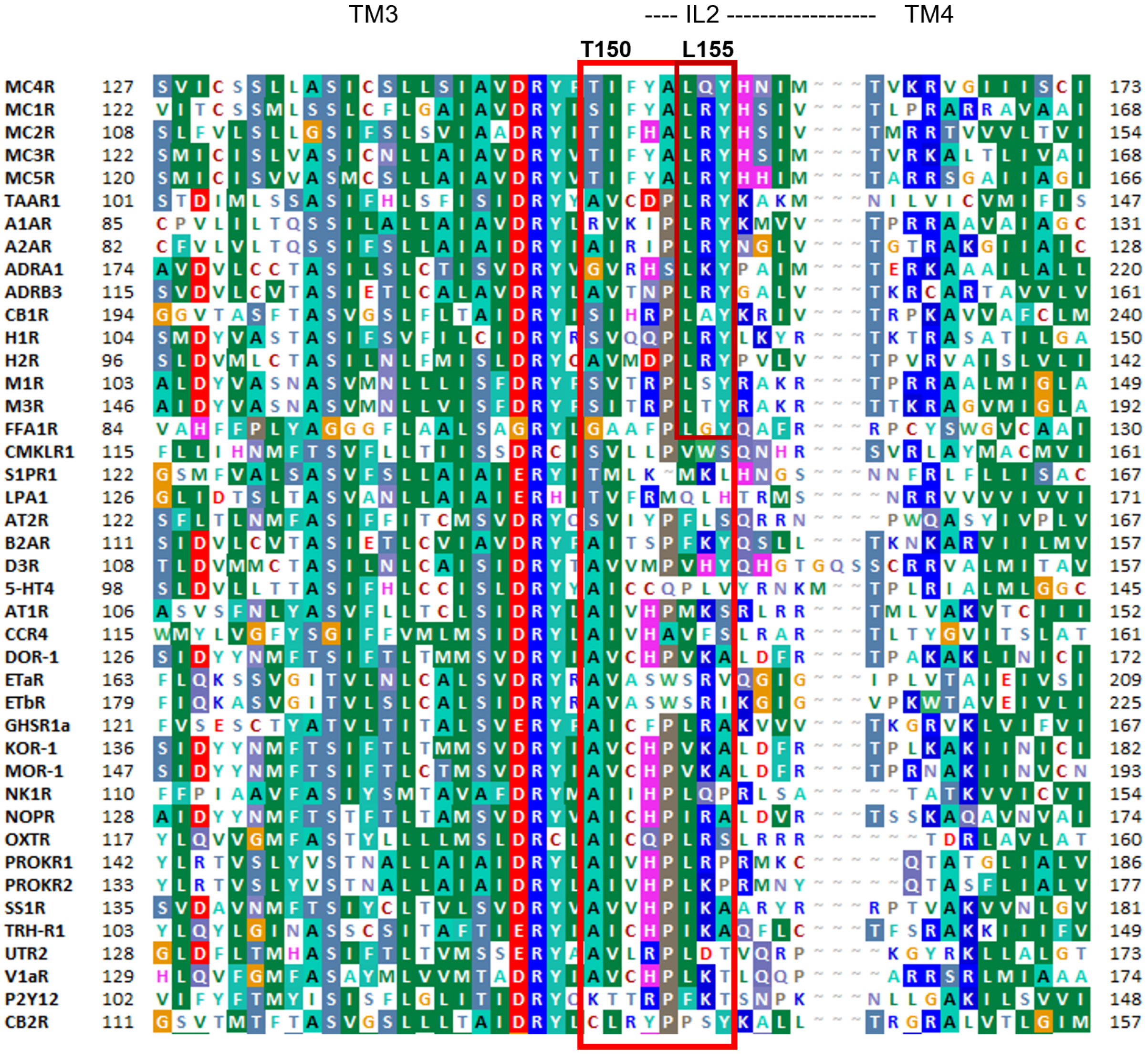

Fig. S18: ***Intracellular loop two (IL2) amino acids in various class A GPCRs.*** The structural dimension of the MC4R IL2 is indicated above respective sequence, as well as the in the main text discussed specific residues T150$^{3.53}$ (TM3) and L155^{IL2}. The alignment was visualized using the software BioEdit (4). Background colors indicating conservation (Blossum62 matrix) among different receptors and reflecting chemical properties of the amino acid side chains: black - proline; blue - positively charged; cyan/green - aromatic and hydrophobic; green - hydrophobic; red - negatively charged; gray - hydrophilic; dark red - cysteines; and magenta - histidine.
