# Supplementary material for: Structures of active melanocortin-4 receptor–Gs-protein complexes with NDP-α-MSH and setmelanotide
Source: Cell Res. 2021 Sep 24;31(11):1176–89. doi: 10.1038/s41422-021-00569-8 (PMC8563958; doi:10.1038/s41422-021-00569-8)
Supplement: Supplementary file 19 — Supplementary figure 19 [file 41422_2021_569_MOESM19_ESM.pdf]

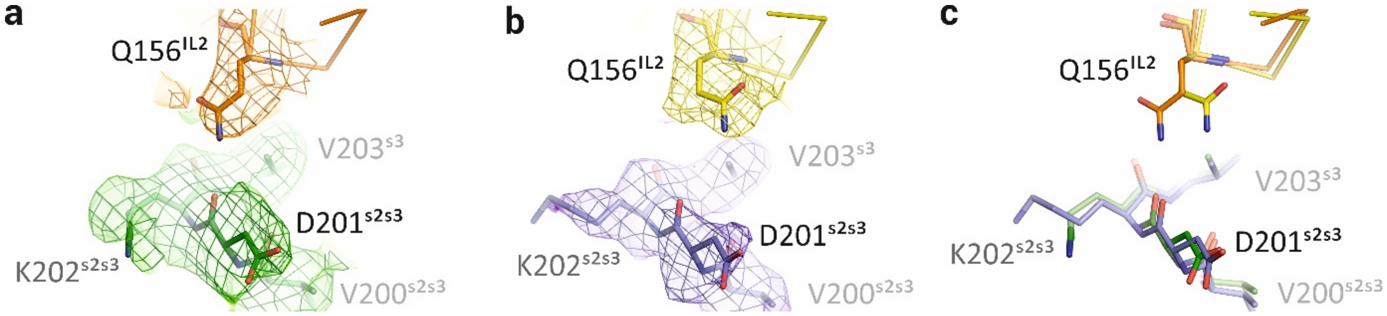

Fig. S19: ***Cryo-EM map differences of the NDP-α-MSH−MC4R−Gs−Nb35 and setmelanotide−MC4R−Gs−Nb35 complexes at Q156^{IL2} in the IL2−Gs interface.***

(**a**) Close-up view on the cryo-EM maps for Q156^{IL2} in the NDP-α-MSH−MC4R−Gs−NB35 and

(**b**) setmelanotide−MC4R−Gs−Nb35 complex and

(**c**) the superposition of both complexes.

By comparison, both agonist-bound MC4R complexes display two different rotamers for Q156^{IL2}, which results in the case of the NDP-α-MSH−MC4R−Gs−Nb35 complex in a hydrogen bond distance between Q156^{IL2} and D201^{s2s3}. This subtle difference among setmelanotide and NDP-α-MSH activated MC4R indicates that MC4R agonists modulate G-protein binding at IL2.

NDP-α-MSH−MC4R, the corresponding Gs-protein, setmelanotide−MC4R and its Gs-protein are colored orange, dark green, yellow, and slate, respectively. Amino acids are shown as sticks. The protein is depicted as ribbon. Cryo-EM maps are displayed as mesh and volume, contoured at 4 σ level and colored corresponding to the displayed proteins.
