# Supplementary material for: Structures of active melanocortin-4 receptor–Gs-protein complexes with NDP-α-MSH and setmelanotide
Source: Cell Res. 2021 Sep 24;31(11):1176–89. doi: 10.1038/s41422-021-00569-8 (PMC8563958; doi:10.1038/s41422-021-00569-8)
Supplement: Supplementary file 20 — Supplementary figure S20 [file 41422_2021_569_MOESM20_ESM.pdf]

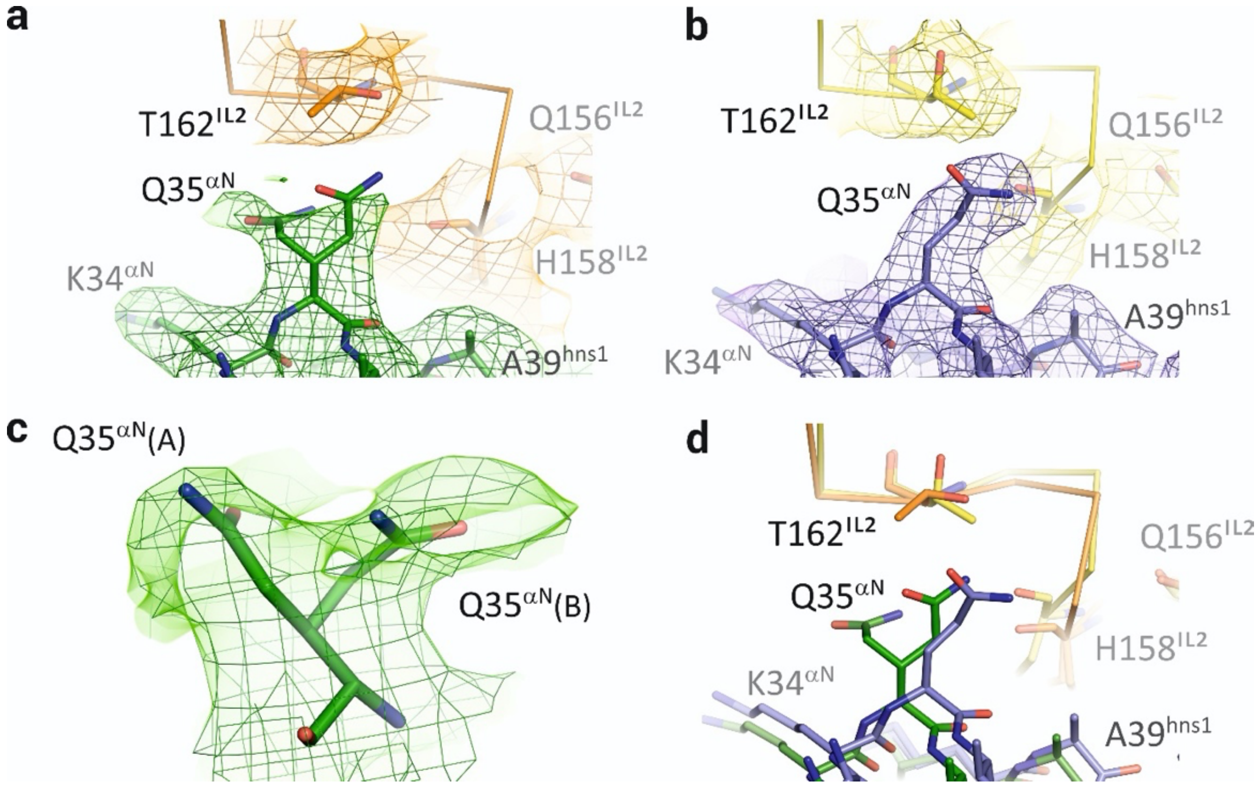

Fig. S20: ***Cryo-EM map differences of the NDP-α-MSH−MC4R−Gs−Nb35 and setmelanotide−MC4R−Gs−Nb35 complexes at T162^{IL2} in the IL2−Gs interface.***

(**a**) Close-up view on the cryo-EM maps for the amino acid T162^{IL2} in the NDP-α-MSH−MC4R−Gs−Nb35 and

(**b**) setmelanotide−MC4R−Gs−Nb35 complex and

(**d**) the superposition of both complexes.

At T162^{IL2} different rotamers were observed for both agonist-bound complexes, whereby only in the NDP-α-MSH−MC4R−Gs−Nb35 complex the hydroxyl group of T162^{IL2} is in hydrogen bond distance to Nε2-atom of Q35$^{\alpha N}$.

NDP-α-MSH−MC4R, the corresponding Gs-protein, setmelanotide−MC4R and its Gs-protein are colored orange, dark green, yellow and slate, respectively. Amino acids are depicted in stick representation. The protein is visualized as ribbon. Cryo-EM maps are displayed as mesh and volume. In the upper row, the cryo-EM maps are contoured at 4 σ level.

(**c**) To verify the double conformation of Q35$^{\alpha N}$, the contour level is set to 2 σ level. All cryo-EM maps are colored corresponding to the displayed proteins.
