# Supplementary material for: Structures of active melanocortin-4 receptor–Gs-protein complexes with NDP-α-MSH and setmelanotide
Source: Cell Res. 2021 Sep 24;31(11):1176–89. doi: 10.1038/s41422-021-00569-8 (PMC8563958; doi:10.1038/s41422-021-00569-8)
Supplement: Supplementary file 21 — Supplementary figure S21 [file 41422_2021_569_MOESM21_ESM.pdf]

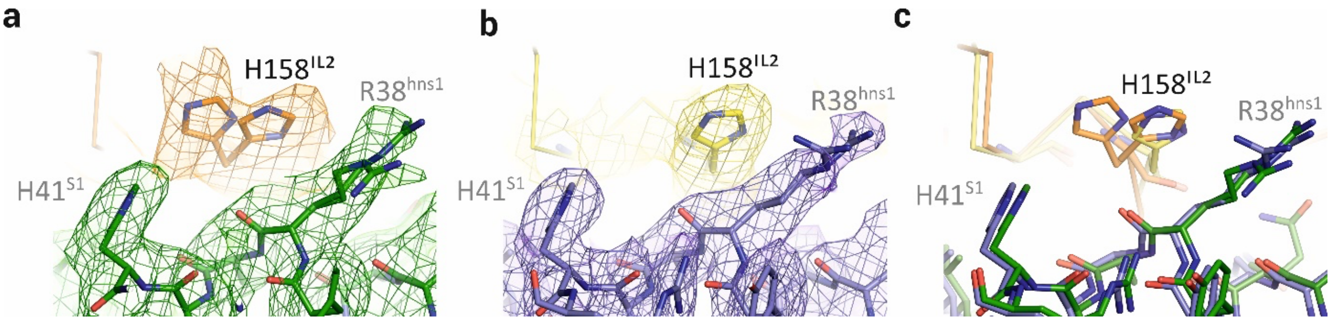

Fig. S21*: Cryo-EM map differences of the NDP-α-MSH−MC4R−Gs−Nb35 and setmelanotide−MC4R−Gs−Nb35 complexes at H158^{IL2} in the IL2−Gs interface.*

(**a**) Close-up view shows for H158^{IL2} a double conformation in the NDP-α-MSH−MC4R−Gs−Nb35 complex,

(**b**) but not for the setmelanotide−MC4R−Gs−Nb35 complex.

(**c**) Superimposition of both complexes underlines the structural differences between the two activated MC4R and point out that MC4R agonists effect the IL2 conformation. NDP-α-MSH−MC4R, the corresponding Gs-protein, setmelanotide−MC4R and its Gs-protein are colored orange, dark green, yellow and slate, respectively. Amino acids are visualized as sticks and the protein as ribbon. Cryo-EM maps are displayed as mesh and volume, contoured at 4 σ level and colored corresponding to the displayed proteins.
