# Supplementary material for: Structures of active melanocortin-4 receptor–Gs-protein complexes with NDP-α-MSH and setmelanotide
Source: Cell Res. 2021 Sep 24;31(11):1176–89. doi: 10.1038/s41422-021-00569-8 (PMC8563958; doi:10.1038/s41422-021-00569-8)
Supplement: Supplementary file 22 — Supplementary figure S22 [file 41422_2021_569_MOESM22_ESM.pdf]

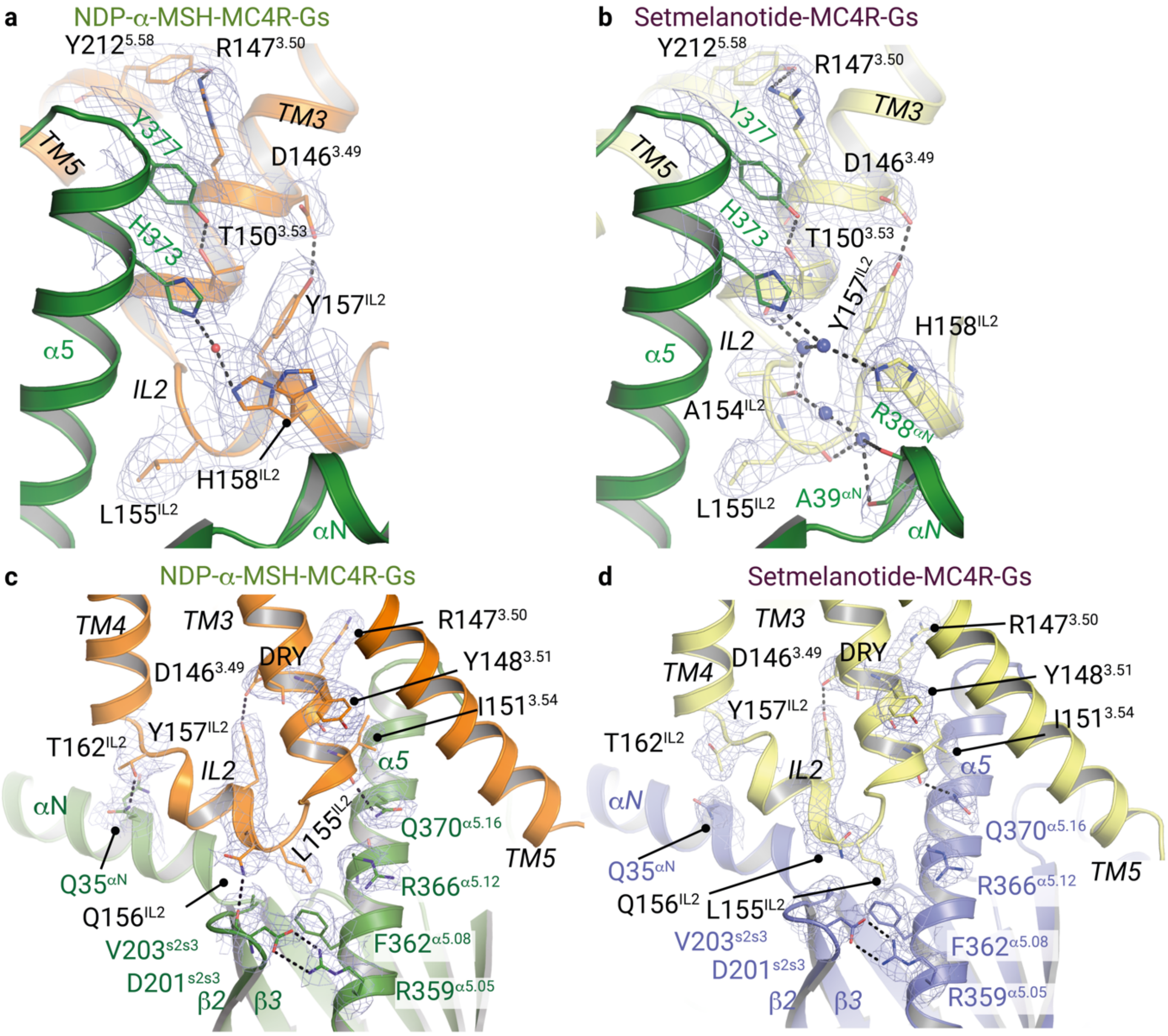

Fig. S22*: Cryo-EM map of selected residues at the MC4R−Gs protein interface.*

(**a-b**) Corresponding cryo-EM densities of residues highlighted in main Fig. 6d (**a**) and 6e (**b**) at a contour level of 1.2 σ.

(**c-d**) Corresponding cryo-EM densities of residues highlighted in main Fig. 7a (**c**) and 7b (**d**) at a contour level of 4 σ. For further details see legend Fig. 7.
