# Supplementary material for: Structures of active melanocortin-4 receptor–Gs-protein complexes with NDP-α-MSH and setmelanotide
Source: Cell Res. 2021 Sep 24;31(11):1176–89. doi: 10.1038/s41422-021-00569-8 (PMC8563958; doi:10.1038/s41422-021-00569-8)
Supplement: Supplementary file 23 — Supplementary figure S23 [file 41422_2021_569_MOESM23_ESM.pdf]

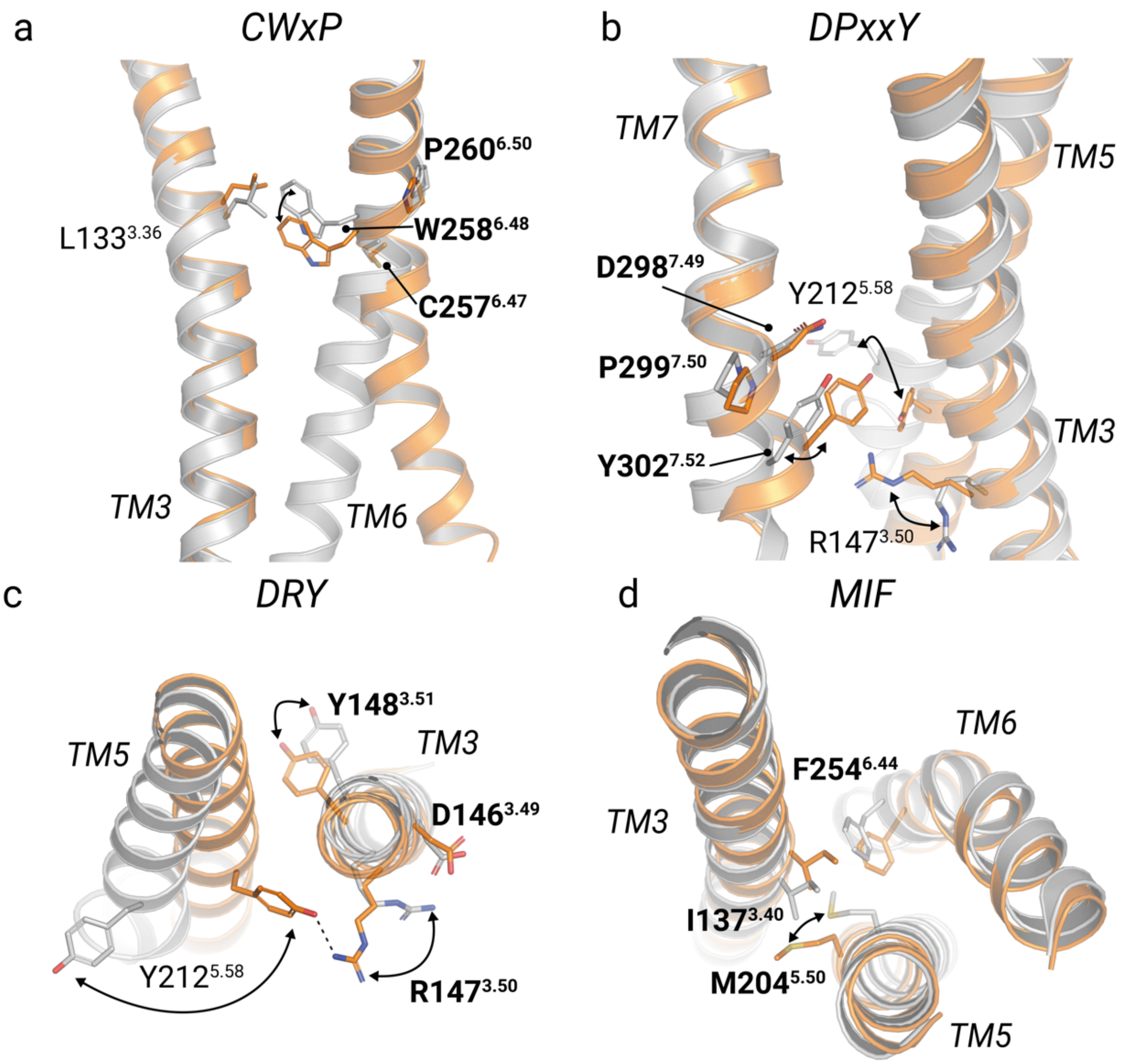

Fig. S23*: Comparison of amino acid side chains in highly conserved MC4R motifs between the inactivated and activated MC4R structures.*

(**a**) The *CWxP* motif with $W^{6.48}$ has been described in several GPCRs as central for the "toggle switch" activation mechanism. In MC4R $W258^{6.48}$ undergoes a toggle switch like motion after agonist binding and is located at the pivot point of the TM6 outward movement.

(**b**) The conserved *NPxxY* motif of class A GPCRs is in MC4R a *DPxxY* motif. Upon activation $Y302^{7.52}$ gets directed towards TM5 and TM7 and interacts with the $Y^{5.58}$ in TM5, which is crucial for the active state structure stabilization.

(**c**) The *DRY* motif is located at the top of the G-protein binding cavity and $R147^{3.50}$ is stabilized by $Y212^{5.58}$ in the active state. Additionally, in Fig. 6 the TM3-TM6 lock of the inactive state and in Fig. 7 the $D146^{3.49}$-$Y157^{IL}$ interaction that stabilizes IL2 are highlighted.

(**d**) Most class A GPCRs are characterized by a bulge and kink supporting proline in TM5. In MC4R instead $M204^{5.50}$ leads to a regular helical conformation without any king or bulge. This residue together with $I137^{3.40}$ in TM3 and $F254^{6.54}$ in TM6 forms the *MIF* motif, reminiscent to the so called "*PIF*" motif of class A GPCRs involved in regulation of the activity state.
