# Supplementary material for: Structures of active melanocortin-4 receptor–Gs-protein complexes with NDP-α-MSH and setmelanotide
Source: Cell Res. 2021 Sep 24;31(11):1176–89. doi: 10.1038/s41422-021-00569-8 (PMC8563958; doi:10.1038/s41422-021-00569-8)
Supplement: Supplementary file 24 — Supplementary figure S24 [file 41422_2021_569_MOESM24_ESM.pdf]

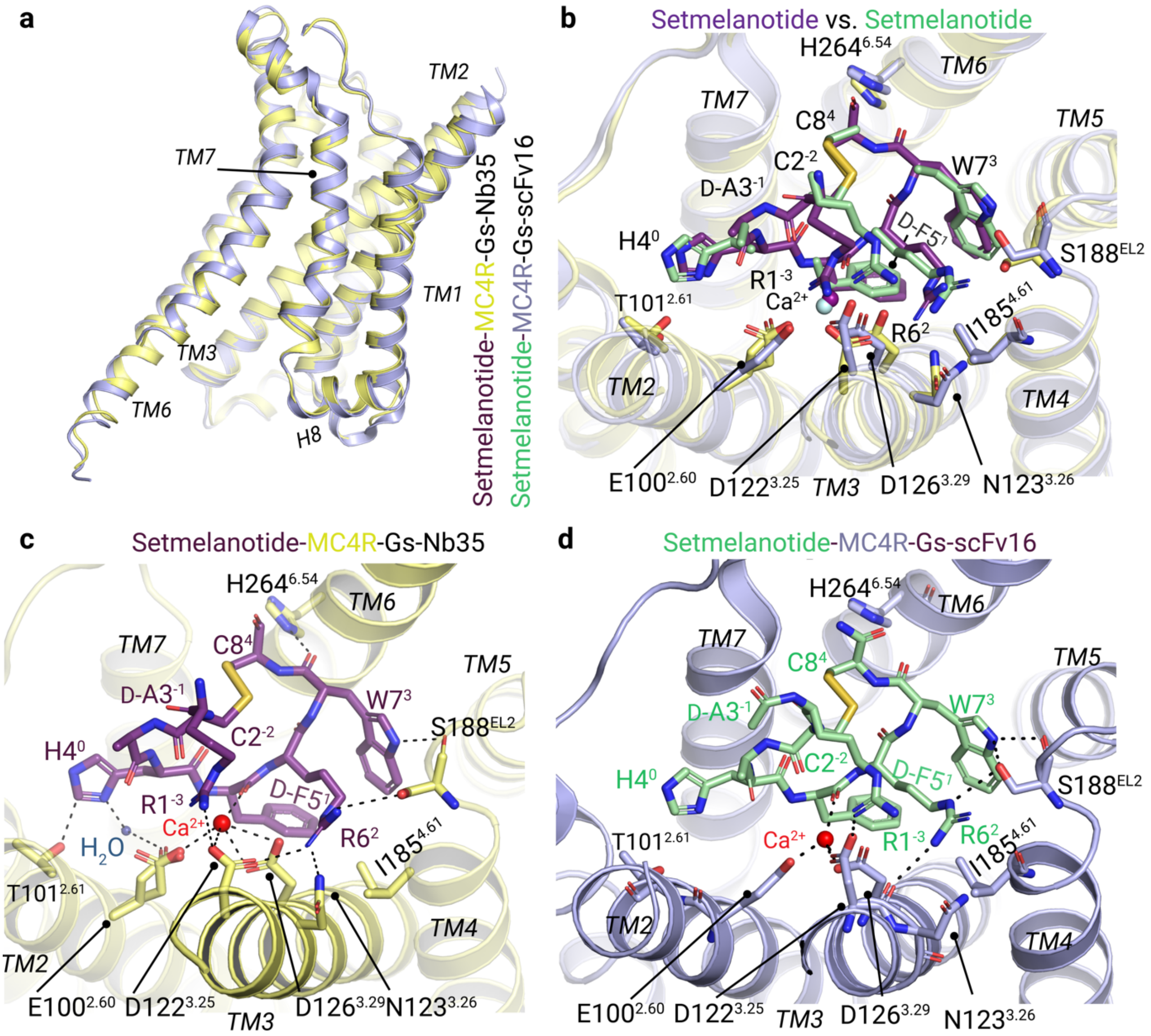

Fig. S24: ***Structural comparison of the setmelanotide−MC4R−Gs complex structure stabilized by Nb35 (this work) and the setmelanotide bound MC4R−G protein complex stabilized by scFv16 fragment[14] display a comparable overall structure and peptide binding mode.***

(**a**) Overall comparison of the receptor backbones represented as cartoons, our setmelanotide−MC4R−Gs−Nb35 complex is colored yellow and the recently published setmelanotide−MC4R−Gs−scFv16 complex[14] is colored in light blue.

(**b**) Superposition of setmelanotide binding modes to MC4R that are shown in (**c-d**). Ca^{2+} is displayed as spheres and is colored according to (structure specific) setmelanotide color. Setmelanotide from the scFv16 stabilized complex is shown as cyan sticks and setmelanotide from the Nb35 stabilized complex as purple sticks.

(**c-d**) Setmelanotide binding mode to MC4R is shown from the here presented structure (**c**) and the recently published structure stabilized by scFv16 (**d**). Hydrogen bonds are shown as black dashed lines and Ca^{2+} as red sphere.
