# Supplementary material for: Structures of active melanocortin-4 receptor–Gs-protein complexes with NDP-α-MSH and setmelanotide
Source: Cell Res. 2021 Sep 24;31(11):1176–89. doi: 10.1038/s41422-021-00569-8 (PMC8563958; doi:10.1038/s41422-021-00569-8)
Supplement: Supplementary file 25 — Supplementary table S1 [file 41422_2021_569_MOESM25_ESM.pdf]

Table S1: ***Data collection, processing, and refinement statistics***

	**NDP-α-MSH-MC4R-$G_s\alpha\beta\gamma$-Nb35 complex** PDB entry 7PIV EMDB entry EMD-13454	**Setmelanotide-MC4R-$G_s\alpha\beta\gamma$-Nb35 complex** PDB entry 7PIU EMDB entry EMD-13453
Magnification	96000	
Voltage (kV)	300	
Electron exposure (e^-/ Å²)	40	
Defocus range (μm)	-0.8 / -2.0	
Pixel size (Å)	0.832	
Symmetry imposed	*C1*	
Micrographs used (total)	5403 (5618)	6979 (7583)
Initial particle image (no.)	2746119	4330500
Final particle images (no.)	221682	370621
Map resolution (Å)	2.86	2.58
FSC threshold	0.143	0.143
**Refinement**		
Initial model used (PDB entries)	6W25 / 3SN6	NDP-MC4R-$G_s\alpha\beta\gamma$-Nb35
Model resolution (Å)	2.88	2.60
Model resolution range (Å)	233 – 2.88	233 – 2.60
Map sharpening B factor (Å²)	-99	-36
**Model composition**		
Total atoms	7814	7788
Water	115	88
Protein atoms	7696	7696
**B-factors (Å²)**		
Overall	69.37	48.11
R.m.s. deviations bond length (Å)	0.013	0.013
R.m.s. deviations bond angle (°)	1.705	1.707
**Validation**		
Molprobity score	1.54	1.42
Clash score	3.53	3.39
Poor rotamers (%)	0.86	0.97
**Ramachandran plot**		
Favoured (%)	94.09	95.77
Allowed (%)	5.70	4.13
Disallowed (%)	0.21	0.11
