# Supplementary material for: Structures of active melanocortin-4 receptor–Gs-protein complexes with NDP-α-MSH and setmelanotide
Source: Cell Res. 2021 Sep 24;31(11):1176–89. doi: 10.1038/s41422-021-00569-8 (PMC8563958; doi:10.1038/s41422-021-00569-8)
Supplement: Supplementary file 26 — Supplementary table S2 [file 41422_2021_569_MOESM26_ESM.pdf]

Table S2: ***Contact distances between MC4R and NDP-α-MSH.*** Interactions between the binding peptide and the MC4R were analyzed using CONTACT, a program of the CCP4 software suite [15]. Interactions were calculated between any atoms of the peptide and the receptor, water molecules as well as the calcium ion with a maximum distance of 3.9 Å. Potential hydrogen bonds with a maximum distance of 3.5 Å are highlighted bold. D-phenylalanine and norleucine are abbreviated as three letter codes Dpn and Nle, respectively.

Source	Number/ Chain	Atom	Target	Number/ Chain	Atom	Distance [Å]
Phe	51R	CD2	His	6P	CE1	3.83
			His	6P	NE2	3.31
Phe	51R	CE2	His	6P	CE1	3.44
			His	6P	CD2	3.69
			His	6P	NE2	3.24
Phe	51R	CZ	His	6P	CD2	3.76
			His	6P	NE2	3.81
Glu	100R	CD	Nle	4P	CE	3.76
Glu	100R	OE2	Nle	4P	CE	3.63
			Glu	5P	O	3.66
			**Ca**	**1F**	**CA**	**3.20**
Thr	101R	CA	His	6P	NE2	3.89
Thr	101R	OG1	His	6P	CD2	3.64
			**His**	**6P**	**NE2**	**3.06**
Ile	104R	CG2	Nle	4P	CE	3.09
Asp	122R	CG	Nle	4P	CB	3.60
			Nle	4P	CD	3.62
			Nle	4P	CE	3.89
Asp	122R	OD1	Nle	4P	CB	3.77
			Nle	4P	CG	3.88
			Nle	4P	CD	2.91
			Nle	4P	CE	3.14
Asp	122R	OD2	Nle	4P	CB	2.93
			Nle	4P	CG	3.87
			Nle	4P	CD	3.68
			Nle	4P	CE	3.85
			**Ca**	**1F**	**CA**	**3.09**
Asp	126R	CB	Arg	8P	NH2	3.64
Asp	126R	CG	Arg	8P	NE	3.68
			Arg	8P	NH2	3.69
			Ca	1F	CA	3.50
**Asp**	**126R**	**OD1**	**Ca**	**1F**	**CA**	**3.33**
Asp	126R	OD2	Dpn	7P	O	3.78
			Arg	8P	CD	3.72
			**Arg**	**8P**	**NE**	**2.59**
			Arg	8P	CZ	3.19
			**Arg**	**8P**	**NH2**	**2.95**
			**Ca**	**1F**	**CA**	**2.93**
Ile	129R	CG2	Dpn	7P	CG	3.64
			Dpn	7P	CD2	3.43
			Dpn	7P	CE2	3.64
Ile	129R	CD1	Dpn	7P	CB	3.68
**Ile**	**185R**	**O**	**Arg**	**8P**	**NH1**	**3.50**

Ile	185R	CG2	Arg	8P	NH2	3.39
Ser	188R	CA	Trp	9P	CZ2	3.86
			Trp	9P	NE1	3.76
Ser	188R	C	Trp	9P	NE1	3.60
Ser	188R	O	Trp	9P	CD1	3.87
			**Trp**	**9P**	**NE1**	**2.95**
Ser	188R	CB	Arg	8P	NH1	3.28
			Trp	9P	CZ2	3.81
			Trp	9P	NE1	3.75
Ser	188R	OG	Arg	8P	CZ	3.88
			**Arg**	**8P**	**NH1**	**2.64**
Val	193R	CG1	Trp	9P	CZ2	3.82
Ile	194R	CG1	Trp	9P	CD1	3.88
			Trp	9P	NE1	3.68
Ile	194R	CD1	Trp	9P	CD1	3.64
			Trp	9P	NE1	3.75
Leu	197R	CD1	Trp	9P	CZ3	3.78
			Trp	9P	CH2	3.63
His	264R	CD2	Trp	9P	O	3.71
His	264R	CE1	Gly	10P	O	3.64
His	264R	NE2	Gly	10P	O	3.67
			**Trp**	**9P**	**O**	**3.03**
Tyr	268R	CD2	Trp	9P	O	3.18
Tyr	268R	CE2	Trp	9P	CB	3.86
			Trp	9P	CA	3.73
			Trp	9P	C	3.76
			Lys	11P	O	3.27
			Trp	9P	O	3.25
			Pro	12P	CA	3.86
			Pro	12P	O	3.59
Tyr	268R	CZ	Pro	12P	O	3.56
Tyr	268R	OH	Lys	11P	O	3.90
			Pro	12P	O	3.55
Phe	284R	CG	Gly	10P	CA	3.49
Phe	284R	CD1	Gly	10P	CA	3.60
Phe	284R	CD2	Gly	10P	CA	3.43
Phe	284R	CE1	Gly	10P	CA	3.68
Phe	284R	CE2	His	6P	O	3.43
			Gly	10P	CA	3.51
Phe	284R	CZ	Gly	10P	CA	3.63
			Arg	8P	O	3.43
			Gly	10P	N	3.78
Leu	288R	CD1	His	6P	CB	3.55
Leu	288R	CD2	Dpn	7P	CD2	3.89
**Ca^{2+}**	**1F**	**CA**	**Glu**	**5P**	**O**	**2.68**
			Glu	5P	C	3.88
**Ca^{2+}**	**1F**	**CA**	**Dpn**	**7P**	**O**	**2.50**
			Dpn	7P	C	3.72
**Nle**	**4P**	**O**	**water**	**43H**	**O**	**2.78**
