# Supplementary material for: Structures of active melanocortin-4 receptor–Gs-protein complexes with NDP-α-MSH and setmelanotide
Source: Cell Res. 2021 Sep 24;31(11):1176–89. doi: 10.1038/s41422-021-00569-8 (PMC8563958; doi:10.1038/s41422-021-00569-8)
Supplement: Supplementary file 27 — Supplementary table S3 [file 41422_2021_569_MOESM27_ESM.pdf]

Table S3: ***Contact distances between MC4R and the agonist setmelanotide.*** Interactions between the binding peptide and the MC4R were analyzed using CONTACT, a program of the CCP4 software suite [15]. Interactions were calculated between any atoms of the peptide and the receptor, water molecules as well as the calcium ion with a maximum distance of 3.9 Å. Potential hydrogen bonds with a maximum distance of 3.5 Å are highlighted bold. D-phenylalanine and D-alanine are abbreviated as three letter codes Dpn and Dal, respectively. *Amino acids in double conformation (amino acid rotamers A and B).

Source	Number/ Chain	Atom	Target	Number/ Chain	Atom	Distance [Å]
Phe	51R	CD2	His	4P	CE1	3.56
Phe	51R	CE2	His	4P	CE1	3.47
			His	4P	NE2	3.46
Phe	51R	CZ	His	4P	CE1	3.74
			His	4P	NE2	3.83
			His	4P	ND1	3.88
			His	4P	CE1	3.56
Glu(A)*	100R	OE1	Dpn	5P	N	3.57
**Glu(A)***	**100R**	**OE2**	**Ca**	**1F**	**CA**	**3.02**
Glu(B)*	100R	OE1	Dal	3P	O	3.88
**Glu(B)***	**100R**	**OE2**	**Ca**	**1F**	**CA**	**2.90**
Thr	101R	CB	His	4P	CE1	3.84
**Thr**	**101R**	**OG1**	**His**	**4P**	**ND1**	**3.39**
			His	4P	CE1	2.86
Ile	104R	CG1	Dal	3P	O	3.69
Ile	104R	CD1	Dal	3P	O	3.89
Asp	122R	CG	Arg	1P	NH1	3.81
**Asp**	**122R**	**OD1**	**Arg**	**6P**	**NH1**	**3.12**
Asp	122R	OD2	Arg	1P	CZ	3.58
			**Arg**	**1P**	**NH1**	**2.80**
			**Ca**	**1F**	**CA**	**3.35**
**Asn**	**123R**	**ND2**	**Arg**	**6P**	**NH1**	**3.16**
Asp	126R	CB	Arg	6P	NH1	3.72
Asp	126R	CG	Arg	6P	NE	3.67
			Arg	6P	NH1	3.35
			Ca	1F	CA	3.34
**Asp**	**126R**	**OD1**	**Ca**	**1F**	**CA**	**2.87**
**Asp**	**126R**	**OD2**	**Dpn**	**5P**	**O**	**3.42**
			**Arg**	**6P**	**NE**	**2.65**
			**Arg**	**6P**	**NH1**	**2.54**
			**Ca**	**1F**	**CA**	**3.07**
			Arg	6P	CD	3.88
			Arg	6P	CZ	2.95
			Arg	6P	CB	3.88
Ile	129R	CG2	Dpn	5P	CG	3.61
			Dpn	5P	CD1	3.86
			Dpn	5P	CD2	3.61
			Dpn	5P	CE2	3.88
Ile	129R	CD1	Dpn	5P	CB	3.82
Leu	133R	CD1	Dpn	5P	CE2	3.46
			Dpn	5P	CZ	3.69

Ser	188R	CA	Trp	7P	NE1	3.66
Ser	188R	C	Trp	7P	NE1	3.62
Ser	188R	O	Trp	7P	CD1	3.79
			**Trp**	**7P**	**NE1**	**3.07**
Ser	188R	CB	Arg	6P	CD	3.56
			Trp	7P	NE1	3.50
**Ser**	**188R**	**OG**	**Arg**	**6P**	**NH2**	**3.42**
Ile	194R	CD1	Trp	7P	CD2	3.57
			Trp	7P	CG	3.68
			Trp	7P	CD1	3.63
			Trp	7P	NE1	3.52
			Trp	7P	CE2	3.48
Leu	197R	CD1	Trp	7P	CH2	3.81
Phe	261R	CZ	Dpn	5P	CE2	3.49
His	264R	CD2	Trp	7P	O	3.58
His	264R	CE1	Cys	8P	C	3.80
His	264R	NE2	Cys	8P	CA	3.67
			Cys	8P	C	3.65
			Trp	7P	C	3.90
			**Trp**	**7P**	**O**	**2.91**
Tyr	268R	CG	Trp	7P	O	3.71
Tyr	268R	CD1	Trp	7P	C	3.86
			Trp	7P	O	3.15
Tyr	268R	CE1	Trp	7P	CA	3.84
			Trp	7P	C	3.52
			Trp	7P	O	3.27
Tyr	268R	CE2	Trp	7P	CB	3.82
Tyr	268R	CZ	Trp	7P	CA	3.69
			Trp	7P	CB	3.89
			Trp	7P	O	3.89
Tyr	268R	OH	Trp	7P	CA	3.81
Phe	284R	CG	Cys	8P	CA	3.82
			Cys	8P	CB	3.76
Phe	284R	CD2	His	4P	O	3.72
			Cys	8P	CA	3.88
			Cys	8P	CB	3.39
Phe	284R	CE1	Arg	6P	O	3.68
Phe	284R	CE2	His	4P	O	3.03
			Cys	8P	CB	3.71
			Arg	6P	O	3.86
Phe	284R	CZ	Arg	6P	N	3.86
			Arg	6P	O	3.08
Asn	285R	ND2	His	4P	CD2	3.51
Leu	288R	CD1	His	4P	CB	3.49
**Ca^{2+}**	**1F**	**CA**	**Dpn**	**5**	**O**	**2.64**
Ca^{2+}	1F	CA	Dpn	5	C	3.82
**water**	**30H**	**O**	**His**	**4P**	**N**	**3.41**
**water**	**62H**	**O**	**His**	**4P**	**NE2**	**2.50**
**water**	**63H**	**O**	**Arg**	**1P**	**NE**	**3.44**
