# Supplementary material for: Structures of active melanocortin-4 receptor–Gs-protein complexes with NDP-α-MSH and setmelanotide
Source: Cell Res. 2021 Sep 24;31(11):1176–89. doi: 10.1038/s41422-021-00569-8 (PMC8563958; doi:10.1038/s41422-021-00569-8)
Supplement: Supplementary file 28 — Supplementary table S4 [file 41422_2021_569_MOESM28_ESM.pdf]

Table S4: ***Contact distances between MC4R and the antagonist SHU9119.*** Interactions between the binding peptide and the MC4R were analyzed using CONTACT, a program of the CCP4 software suite [15]. Interactions were calculated between any atoms of the peptide and the receptor, water molecules as well as the calcium ion with a maximum distance of 3.9 Å. Potential hydrogen bonds with a maximum distance of 3.5 Å are highlighted bold. D-Nal corresponds to (2R)-2-amino-3-(naphthalene-2-YL)propanoic acid abbreviated as 4J2 in the protein database (PDB ID: 6w25). Norleucine is shown as Nle in the three letter code. At the second position of the SHU9119 peptide there is a homoserine. In the corresponding pdb file (PDB ID: 6w25), this amino acid is abbreviated as Asp B2.

Source	Number/ Chain	Atom	Target	Number/ Chain	Atom	Distance [Å]
Leu	97A	CD2	DNal	4B	CB	3.80
Glu	100A	CD	Asp	2B	O	3.77
			His	3B	CA	3.86
			DNal	4B	N	3.45
			Ca	2101A	CA	3.37
Glu	100A	OE1	His	3B	CA	3.45
			His	3B	C	3.56
			**DNal**	**4B**	**N**	**2.78**
			DNal	4B	CA	3.71
			DNal	4B	CB	3.53
			**Ca**	**2101A**	**CA**	**2.21**
Glu	100A	OE2	DNal	4B	O	3.59
			**Asp**	**2B**	**O**	**3.25**
			**DNal**	**4B**	**N**	**3.48**
			**Ca**	**2101A**	**CA**	**2.21**
Thr	101A	CA	His	3B	NE2	3.76
Thr	101A	CB	His	3B	NE2	3.71
Thr	101A	OG1	His	3B	CD2	3.30
			**His**	**3B**	**NE2**	**3.31**
Thr	101A	CG2	His	3B	NE2	3.51
Val	103A	CG1	Nle	1B	CE	3.78
Ile	104A	CG1	Nle	1B	CD	3.82
Ile	104A	CD1	Asp	2B	C	3.66
			His	3B	N	3.52
			His	3B	CG	3.87
			His	3B	ND1	3.80
			His	3B	CE1	3.87
Thr	118A	O	Nle	1B	CE	3.77
Asp	122A	CB	Nle	1B	CB	3.84
			Nle	1B	CD	3.80
			Nle	1B	CE	3.83
Asp	122A	CG	Nle	1B	CB	3.56
			Nle	1B	CD	3.85
			Ca	2101A	CA	3.27
Asp	122A	OD1	Nle	1B	CB	3.74
			Nle	1B	CD	3.47
			Asp	2B	O	3.54
			**Ca**	**2101A**	**CA**	**2.27**
Asp	122A	OD2	Nle	1B	CB	3.78

			**Arg**	**5B**	**NH1**	**3.18**
			Ca	2101A	CA	3.65
Asn	123A	CA	Arg	5B	NH1	3.79
Asn	123A	CG	Arg	5B	NH1	3.83
Asn	123A	OD1	Arg	5B	NH2	3.79
			Arg	5B	CZ	3.72
			**Arg**	**5B**	**NH1**	**2.83**
Asp	126A	O	DNal	4B	CE3	3.56
Asp	126A	CB	Arg	5B	NE	3.52
Asp	126A	CG	DNal	4B	O	3.55
			Arg	5B	NH1	3.83
			Arg	5B	NE	3.38
			Ca	2101A	CA	3.04
**Asp**	**126A**	**OD1**	**DNal**	**4B**	**O**	**3.02**
			DNal	4B	C	3.61
			DNal	4B	CD2	3.60
			DNal	4B	CB	3.50
			**Ca**	**2101A**	**CA**	**2.64**
Asp	126A	OD2	DNal	4B	O	3.54
			Arg	5B	CZ	3.26
			**Arg**	**5B**	**NH1**	**2.85**
			**Arg**	**5B**	**NE**	**2.80**
			**Ca**	**2101A**	**CA**	**2.73**
Ile	129A	CG2	DNal	4B	CD2	3.81
			DNal	4B	CZ1	3.58
			DNal	4B	CE2	3.65
			DNal	4B	CD1	3.82
			DNal	4B	CE1	3.67
			DNal	4B	CB	3.61
			DNal	4B	CG	3.82
Cys	130A	CA	DNal	4B	CE4	3.69
Cys	130A	CB	DNal	4B	CE4	3.50
Cys	130A	SG	DNal	4B	CE4	3.78
Leu	133A	CD2	DNal	4B	CZ2	3.84
			DNal	4B	CZ3	3.63
**Ile**	**185A**	**O**	**Arg**	**5B**	**NH2**	**3.36**
Ile	185A	CG2	Arg	5B	NH2	3.73
			Arg	5B	CZ	3.61
			Arg	5B	NH1	3.72
Ile	185A	CD1	Arg	5B	CD	3.66
			DNal	4B	CE3	3.82
Ser	188A	C	Trp	6B	NE1	3.54
Ser	188A	O	Trp	6B	CD1	3.69
			**Trp**	**6B**	**NE1**	**2.78**
			Trp	6B	CE2	3.72
Ser	188A	CB	Arg	5B	NH2	3.47
			Arg	5B	CD	3.69
			Trp	6B	NE1	3.77
			Trp	6B	CZ2	3.85
**Ser**	**188A**	**OG**	**Arg**	**5B**	**NH2**	**2.83**
Ile	194A	CG1	Trp	6B	NE1	3.65
Ile	194A	CD1	Trp	6B	CD1	3.78
			Trp	6B	NE1	3.60
Leu	197A	CD1	Trp	6B	CH2	3.68

			DNal	4B	CE1	3.62
Phe	261A	CE2	DNal	4B	CZ2	3.71
Phe	261A	CZ	DNal	4B	CZ1	3.65
			DNal	4B	CZ2	3.44
			DNal	4B	CE1	3.51
His	264A	CD2	Trp	6B	O	3.28
			Nh2	8B	N	3.32
His	264A	NE2	Trp	6B	C	3.73
			Lys	7B	CA	3.55
			Lys	7B	C	3.77
			**Trp**	**6B**	**O**	**2.70**
			**Nh2**	**8B**	**N**	**3.45**
Leu	265A	CD1	Trp	6B	CB	3.81
			Trp	6B	CE3	3.57
Tyr	268A	CD1	Trp	6B	C	3.75
			Trp	6B	O	3.30
			Trp	6B	CB	3.88
Tyr	268A	CE1	Trp	6B	CA	3.71
			Wat	103B	O	3.63
			Trp	6B	C	3.71
			Lys	7B	O	3.80
			Trp	6B	O	3.71
Tyr	268A	CE2	Trp	6B	CD1	3.56
Tyr	268A	CZ	Trp	6B	CD1	3.90
Tyr	268A	OH	Wat	102B	O	3.89
			**Wat**	**103B**	**O**	**3.47**
Met	281A	O	Nh2	8B	N	3.86
Met	281A	CE	Nh2	8B	N	3.65
Phe	284A	CG	Lys	7B	CB	3.90
Phe	284A	CD2	Lys	7B	CB	3.42
Phe	284A	CE1	Arg	5B	O	3.81
Phe	284A	CE2	Lys	7B	CB	3.62
			His	3B	O	3.17
Phe	284A	CZ	Arg	5B	O	3.30
Leu	288A	CD1	His	3B	CB	3.48
Leu	288A	CD2	DNal	4B	CD1	3.71
**water**	**101B**	**O**	**Asp**	**2B**	**OD1**	**2.41**
**water**	**102B**	**O**	**Trp**	**6B**	**N**	**3.17**
**water**	**103B**	**O**	**Nh2**	**8B**	**O**	**3.23**
**water**	**103B**	**O**	**Water**	**102B**	**O**	**2.85**
**Ca^{2+}**	**2101A**	**CA**	**Asp**	**2B**	**O**	**2.57**
Ca^{2+}	2101A	CA	Asp	2B	C	3.78
**Ca^{2+}**	**2101A**	**CA**	**DNal**	**4B**	**O**	**2.26**
Ca^{2+}	2101A	CA	DNal	4B	C	3.40
Ca^{2+}	2101A	CA	DNal	4B	N	3.80
