# Supplementary material for: Structures of active melanocortin-4 receptor–Gs-protein complexes with NDP-α-MSH and setmelanotide
Source: Cell Res. 2021 Sep 24;31(11):1176–89. doi: 10.1038/s41422-021-00569-8 (PMC8563958; doi:10.1038/s41422-021-00569-8)
Supplement: Supplementary file 29 — Supplementary table S5 [file 41422_2021_569_MOESM29_ESM.pdf]

Table S5: ***Distances between calcium ion and ligand and MC4R binding partners.*** Interactions between the coordinating calcium ion and its binding partners in the three available models of the MC4R binding the peptides NDP-α-MSH, setmelanotide and SHU9119 were analyzed using CONTACT, a program of the CCP4 software suite [15]. Interactions were calculated between any atoms of the peptide and the receptor, water molecules as well as the calcium ion with a maximum distance of 3.9 Å. Potential hydrogen bonds with a maximum distance of 3.5 Å are highlighted bold. Covalent interactions with Ca^{2+} with a maximum distance of 2.4 Å are highlighted bold and red. D-Nal corresponds to (2R)-2-amino-3-(naphthalene-2-YL)propanoic acid abbreviated as 4J2 in the protein database (PDB entry 6w25). At the second position of the SHU9119 peptide there is a homoserine. In the corresponding pdb file (PDB ID: 6w25), this amino acid is abbreviated as Asp B2. D-phenylalanine is abridged as three letter code Dpn.

**Target atoms**			**NDP-α-MSH** (agonist)	**Setmelanotide** (agonist)	**SHU9119** (antagonist); (PDB ID: 6w25)
**Amino acid**	**Number/ Chain**	**Atom**	**Distance [Å]**	**Distance [Å]**	**Distance [Å]**
Glu	100A/R	CD	-	-	3.37
Glu	100A/R	OE1	-	-	**2.21**
Glu	100A/R	OE2	**3.20**	**2.90/3.02**	**2.21**
Asp	122A/R	CG	-	-	**3.27**
Asp	122A/R	OD1	-	-	**2.27**
Asp	122A/R	OD2	**3.09**	**3.35**	3.65
Asp	126A/R	CG	3.50	3.34	3.04
Asp	126A/R	OD1	**3.33**	**2.87**	**2.64**
Asp	126A/R	OD2	**2.93**	**3.07**	**2.73**
Glu	$5P^{-1}$	O	**2.68**	-	-
Glu	$5P^{-1}$	C	3.88	-	-
Dpn	$7P^{1}$	O	**2.50**	-	-
Dpn	$7P^{1}$	C	3.72	-	-
Dpn	$5P^{1}$	O	-	**2.64**	-
Dpn	$5P^{1}$	C	-	3.82	-
His	$4P^{0}$	N	-	**3.41**	-
His	$4P^{0}$	NE2	-	**2.50**	-
Arg	$1P^{-3}$	NE	-	**3.44**	-
Asp	$2B^{-1}$	O	-	-	**2.57**
Asp	$2B^{-1}$	C	-	-	3.78
DNal	$4B^{1}$	O	-	-	**2.26**
DNal	$4B^{1}$	C	-	-	3.40
DNal	$4B^{1}$	N	-	-	3.80
