# Supplementary material for: Structures of active melanocortin-4 receptor–Gs-protein complexes with NDP-α-MSH and setmelanotide
Source: Cell Res. 2021 Sep 24;31(11):1176–89. doi: 10.1038/s41422-021-00569-8 (PMC8563958; doi:10.1038/s41422-021-00569-8)
Supplement: Supplementary file 30 — Supplementary table S6 [file 41422_2021_569_MOESM30_ESM.pdf]

Table S6: ***Comparison of the receptor–ligand contacts in complexes of MC4R with the agonists NDP-α-MSH and setmelanotide as well as the antagonist SHU9119.*** Interactions of all relevant amino acids of the MC4R the calcium ion (Ca^{2+}) and water molecules within the ligand binding pocket towards the binding peptides NDP-α-MSH, setmelanotide and SHU9119 were summarized based on the Tables S2-4. Amino acids were abbreviated as one letter code, capital numbers display the position within the receptor or peptide (in the pdb file), and superscripted numbering refers to the conserved *HxRW* motif of the binding peptides. D-Nal corresponds to (2R)-2-amino-3-(naphthalene-2-YL)propanoic acid abbreviated as 4J2 in the protein database (PDB ID: 6w25). D-phenylalanine, D-alanine and norleucine are abridged as three letter code Dpn, Dal and Nle, respectively.

**MC4R**	**NDP-α-MSH** (agonist); (PDB ID: 7PIV, this study)	**Setmelanotide** (agonist); (PDB ID: 7PIU, this study)	**SHU9119** (antagonist); (PDB ID: 6w25)
F51	H6^{0}	H4^{0}	-
N97 (L97 - antagonized structure)	-	-	Dnal4^{1}
E100	Nle4^{-2}, E5^{-1}	A: Dpn5^{1}<br>B: Dal3^{-1}	H3^{0}, Dnal4^{1}, D2^{-1}
T101	H6^{0}	H4^{0}	H3^{0}
V103	-	-	Nle1^{-2}
I104	Nle4^{-2}	Dal3^{-1}	Nle1^{-2}, D2^{-1}, H3^{0}
T118	-	-	Nle1^{-2}
D122	Nle4^{-2}	R1^{-3}, R6^{2}	Nle1^{-2}, D2^{-1}, R5^{2}
N123	-	R6^{2}	R5^{2}
D126	Dpn7^{1}, R8^{2}	Dpn5^{1}, Arg6^{2}	Dnal4^{1}, Arg5^{2}
I129	Dpn7^{1}	Dpn5^{1}	Dnal4^{1}
C130	-	-	Dnal4^{1}
L133*	-	Dpn5^{1}	Dnal4^{1}
I185*	R8^{2}	-	Dnal4^{1}, R5^{2}
S188	R8^{2}, W9^{3}	R6^{2}, W7^{3}	R5^{2}, W6^{3}
V193	W9^{3}	-	-
I194	W9^{3}	W7^{3}	W6^{3}
L197	W9^{3}	W7^{3}	Dnal4^{1}, W6^{3}
F261	-	Dpn5^{1}	Dnal4^{1}
H264	W9^{3}, G10^{4}	W7^{3}, C8^{4}	W6^{3}, K7^{4}, $NH_2$8^{5}
L265	-	-	W6^{3}
Y268	W9^{3}, K11^{5}, P12^{6}	W7^{3}	W6^{3}, K7^{4}
M281	-	-	$NH_2$8^{5}
F284	H6^{0}, R8^{2}, G10^{4}	H4^{0}, R6^{2}, C8^{4}	H3^{0}, R5^{2}, K7^{4}
N285	-	H4^{0}	-
L288	H6^{0}, Dpn7^{1}	H4^{0}	H3^{0}, Dnal4^{1}
**Ca^{2+}**	MC4R: E100, D122, D126; Ligand: E5^{-1}, Dpn7^{1}	MC4R: E100, D122, D126; Ligand: Dpn5^{1}	MC4R: E100, D122, D126; Ligand: D2^{-1}, Dnal4^{1}
water 30	-	Dal3^{-1}, H4^{0}, C8^{4}, Q43, F284, N285	-
water 43	Nle4^{-2}	-	-

water 63	-	$R1^{-3}$, $R6^{2}$	-
water 101	-	-	$D2^{-1}$, $H3^{0}$, $K7^{4}$,
water 102	-	-	$R5^{2}$, $W6^{3}$, Y268, water 103, water 104
water 103	-	-	$W6^{3}$, $K7^{4}$, Y268, water102, water 105
water 104	-	-	$Dnal4^{-1}$, $R5^{2}$, $W6^{3}$, $K7^{4}$, water 102, water 105
water 105	-	-	$D2^{-1}$, $K7^{4}$, water 103, water 104
