# Supplementary material for: Structures of active melanocortin-4 receptor–Gs-protein complexes with NDP-α-MSH and setmelanotide
Source: Cell Res. 2021 Sep 24;31(11):1176–89. doi: 10.1038/s41422-021-00569-8 (PMC8563958; doi:10.1038/s41422-021-00569-8)
Supplement: Supplementary file 31 — Supplementary table S7 [file 41422_2021_569_MOESM31_ESM.pdf]

Table S7: ***Determination of total and cell surface expression with Nano-Glo®HiBiT Lytic/Extracellular detection system, and maximal cAMP response E_{max} via AlphaScreen®.*** Data are given as the result of four to eight independent experiments performed in triplicates ± SEM. Wild-type MC4R (WT) stimulation as fold over MC4R basal for all assays is 20 ± 1.06 fold for α-MSH, 22 ± 1.15 fold for NDP-α-MSH and 14.53 ± 1.53 for setmelanotide, all set as 100 %. Expression data were cleaned by performing a ROUT test with Q = 1%. Statistics were done by one-way ANOVA with Kruskal-Wallis test. WT was tested against all mutants stimulated with the indicated ligand: a: $p < 0.05$; b: $p < 0.01$; c: $p < 0.001$; d: $p < 0.0001$.

Substi-tution	Total expression [fold over WT basal]	Cell surface expression [fold over WT basal]	Basal [fold over WT basal]	α-MSH E_{max} [% of WT at 1 µM]	NDP-α-MSH E_{max} [% of WT at 1 µM]	Set-melanotide E_{max} [% of WT at 1 µM]
MC4R WT	1	1	1	100	100	100
E100N	1.30 ± 0.10	2.42 ± 0.15^{d}	0.98 ± 0.08	11.35 ± 1.45^{a}	56.10 ± 4.13^{a}	103 ± 13
T101A	1.41 ± 0.07	0.89 ± 0.11	1.16 ± 0.16^{a}	189 ± 10^{d}	155 ± 12^{b}	-
D122S	1.63 ± 0.06^{c}	1.41 ± 0.09	1.16 ± 0.06	40.10 ± 3.46	129 ± 8.56	138 ± 11
N123A	0.53 ± 0.03^{d}	0.60 ± 0.05^{c}	1.05 ± 0.05	163 ± 13^{b}	119 ± 7.89	154 ± 11^{b}
D126S	1.28 ± 0.07	1.87 ± 0.14^{c}	0.90 ± 0.05^{a}	13.92 ± 1.67^{a}	91.23 ± 7.74	81.69 ± 7.77
C130A	0.99 ± 0.04	1.13 ± 0.09	1.03 ± 0.09	147 ± 9.24^{a}	114 ± 9.25	-
L133A	1.11 ± 0.10	0.78 ± 0.15	1.03 ± 0.05	130 ± 6	152 ± 8^{a}	-
L133F	0.97 ± 0.09	1.34 ± 0.11	0.85 ± 0.04^{a}	112 ± 6	137 ± 7	-
I137A	0.83 ± 0.04	0.79 ± 0.03	1.30 ± 0.08	92.78 ± 4.96	99.40 ± 4.89	-
I137F	0.81 ± 0.07	0.57 ± 0.06^{b}	1.02 ± 0.06	76.65 ± 3.93	94.21 ± 5.53	-
T150A	0.60 ± 0.06^{c}	0.58 ± 0.04^{b}	0.76 ± 0.06^{d}	26.11. ± 1.64^{d}	18.07 ± 2.17^{d}	32.41 ± 1.87^{d}
T150I	0.69 ± 0.06^{a}	0.55 ± 0.02^{b}	0.88 ± 0.06^{c}	36.46 ± 2.24^{d}	40.74 ± 2.08^{d}	-
T150D	0.41 ± 0.03^{d}	0.47 ± 0.03^{c}	0.85 ± 0.05	9.88 ± 0.91^{d}	10.85 ± 0.86^{c}	-
T150F	1.09 ± 0.05	0.88 ± 0.03	0.83 ± 0.02^{a}	42.60 ± 2.41^{d}	55.68 ± 4.64^{a}	-
T150S	1.44 ± 0.09	1.23 ± 0.05	0.77 ± 0.03^{c}	77.81 ± 5.11^{a}	86.18 ± 5.14	-
H158A	1.35 ± 0.17	1.37 ± 0.16	2.49 ± 0.19^{d}	129 ± 15	129 ± 15	148 ± 17^{b}
F184V	1.14 ± 0.07	1.82 ± 0.14^{c}	1.24 ± 0.11	149 ± 14	91.65 ± 8.29	-
S188A	1.10 ± 0.13	1.15 ± 0.06	0.94 ± 0.07	148 ± 11^{a}	142 ± 15	-
D189S	0.98 ± 0.06	1.51 ± 0.13	0.80 ± 0.06	151 ± 16	166 ± 28	-
S191A	1.00 ± 0.06	0.89 ± 0.04	1.00 ± 0.04	199 ± 18^{c}	197 ± 22^{c}	-
L197A	0.78 ± 0.06	1.12 ± 0.16	0.84 ± 0.05	152 ± 11	171 ± 24^{a}	-
M204A	0.60 ± 0.04^{c}	0.69 ± 0.04	1.81 ± 0.13^{d}	113 ± 5	103 ± 5	-
L205A	0.92 ± 0.06	0.82 ± 0.08	1.21 ± 0.06	101 ± 7	99.85 ± 5.61	-
L205F	1.14 ± 0.05	1.02 ± 0.04	2.32 ± 0.20^{d}	112 ± 6	112 ± 6	-
F254A	1.73 ± 0.15^{a}	0.94 ± 0.04	1.02 ± 0.03	88.67 ± 5.41	85.09 ± 5.23	-
F254M	1.99 ± 0.17^{b}	1.57 ± 0.10^{a}	0.99 ± 0.04	77.33 ± 8.14	97.12 ± 10.32	-
W258A	0.73 ± 0.07	0.48 ± 0.04^{c}	0.94 ± 0.07	61.40 ± 4.13^{d}	65.04 ± 5.30^{b}	-
W258F	1.30 ± 0.09	0.96 ± 0.06	2.48 ± 0.22^{d}	78.13 ± 4.39^{a}	72.66 ± 4.83	-
F261V	0.81 ± 0.04	0.77 ± 0.10	0.93 ± 0.08	63.54 ± 4.19	61.61 ± 5.96	112 ± 8
H264A	0.47 ± 0.04^{d}	1.35 ± 0.05	1.10 ± 0.07	10.21 ± 0.94^{a}	133 ± 9.10	111 ± 10
L265A	0.62 ± 0.04^{d}	0.78 ± 0.05	0.89 ± 0.08	83.68 ± 6.16	140 ± 12	-
Y268F	0.98 ± 0.07	1.06 ± 0.06	0.87 ± 0.03	140 ± 16	133 ± 14	-
H283A	0.95 ± 0.07	1.17 ± 0.08	1.40 ± 0.18	164 ± 15^{a}	152 ± 21	-
F284V	0.67 ± 0.09^{b}	0.90 ± 0.05	1.08 ± 0.03	145 ± 14	165 ± 27	-

N285S	0.86 ± 0.07	0.99 ± 0.07	1.14 ± 0.05	173 ± 16[a]	167 ± 22	-
Y287V	0.74 ± 0.02	1.75 ± 0.10[b]	0.99 ± 0.04	148 ± 12	142 ± 18	-
L288A	0.51 ± 0.04[d]	0.47 ± 0.05[d]	0.94 ± 0.04	76.41 ± 5.51	129 ± 12	-
I291A	0.49 ± 0.03[d]	0.54 ± 0.05[b]	0.94 ± 0.03	11.09 ± 0.94[d]	8.98 ± 0.69[d]	-
I291F	0.81 ± 0.08	0.54 ± 0.04[c]	0.92 ± 0.05	79.83 ± 6.47	71.34 ± 6.02	-
