# Supplementary material for: Structures of active melanocortin-4 receptor–Gs-protein complexes with NDP-α-MSH and setmelanotide
Source: Cell Res. 2021 Sep 24;31(11):1176–89. doi: 10.1038/s41422-021-00569-8 (PMC8563958; doi:10.1038/s41422-021-00569-8)
Supplement: Supplementary file 32 — Supplementary table S8 [file 41422_2021_569_MOESM32_ESM.pdf]

Table S8: ***Determination of half maximal effective concentration EC_{50} via cAMP accumulation using the AlphaScreen® assay after addition of agonists α-MSH, NDP-α-MSH and setmelanotide.*** Data are given as the result of four to eight independent experiments performed in triplicates ± SEM. Statistics were done by one-way ANOVA with Kruskal-Wallis test. Wild-type MC4R (WT) was tested against all mutants stimulated with the indicated ligand. a: $p < 0.05$; b: $p < 0.01$; c: $p < 0.001$; d: $p < 0.0001$; n.d. = not determined due to either too low E_{max} or due to severely shifted concentration-response curve which do not allow proper EC_{50} calculation.

**Substitution**	**α-MSH EC_{50} [nM]**	**NDP-α-MSH EC_{50} [nM]**	**Setmelanotide EC_{50} [nM]**
MC4R WT	17.6 ± 4.21	1.17 ± 0.26	1.24 ± 0.39
E100N	n.d.	383 ± 139^{c}	n.d
T101A	49.4 ± 6.12	0.9 ± 0.05	-
D122S	n.d.	9.74 ± 1.61^{b}	n.d.
N123A	33.3 ± 5.97	0.93 ± 0.23	6.40 ± 2.82
D126S	n.d.	166 ± 34.2^{c}	n.d
C130A	136 ± 21.9^{c}	2.20 ± 0.71	-
L133A	94.7 ± 1.58	2.07 ± 0.26	-
L133F	76.1 ± 17.0	3.79 ± 0.77	-
I137A	19.7 ± 5.97	1.15 ± 0.08	-
T150A	n.d.	n.d.	1.09 ± 0.08
T150I	86.2 ± 34.9	1.41 ± 0.35	-
T150F	91.9 ± 16.9	2.06 ± 0.58	-
T150S	36.2 ± 9.10	0.76 ± 0.16	-
H158A	7.59 ± 3.83	0.58 ± 0.14	2.4 ± 0.82
S188A	101 ± 30.2	0.97 ± 0.19	-
M204A	6.34 ± 1.17	1.06 ± 0.44	-
L205F	7.18 ± 1.84	1.38 ± 0.45	-
W258A	70.7 ± 17.7	2.53 ± 0.34	-
W258F	2.60 ± 0.53	0.59 ± 0.2	-
F261V	118 ± 14.5^{c}	2.96 ± 1.44	28.80 ± 7.70
H264A	n.d.	179 ± 32.5^{d}	n.d.
