# Supplementary material for: Structures of active melanocortin-4 receptor–Gs-protein complexes with NDP-α-MSH and setmelanotide
Source: Cell Res. 2021 Sep 24;31(11):1176–89. doi: 10.1038/s41422-021-00569-8 (PMC8563958; doi:10.1038/s41422-021-00569-8)
Supplement: Supplementary file 33 — Supplementary table S9 [file 41422_2021_569_MOESM33_ESM.pdf]

Table S9*: Determination of Gq/11 activation by determination of phospholipase C activation via NFAT reporter gene assay*. Data are given as the result of four to six independent experiments performed in triplicated ± SEM. The stimulation of wild-type MC4R (WT) as fold over WT basal is 7.56 ± 0.87 for NDP-α-MSH challenge and 10.91 ± 0.88 for stimulation with setmelanotide at a concentration of 1 µM. Statistics were done by one-way ANOVA with Kruskal-Wallis test. MC4R WT was tested against all mutants stimulated with the indicated ligand: a: $p < 0.05$; b; $p < 0.01$; c: $p < 0.001$; d: $p < 0.0001$; n.d. = not determined due to either too low E_{max} or due to severely shifted concentration-response curve which do not allow proper EC_{50} calculation.

	**Basal**	**NDP-α-MSH**		**Setmelanotide**	
**Variant**	**[fold over MC4R WT basal]**	**E_{max} at 1µM [fold of MC4R WT at 1µM]**	**EC_{50} [nM]**	**E_{max} at 1µM [fold of MC4R WT at 1µM]**	**EC_{50} [nM]**
MC4R WT	1	100	5.81 ± 0.88	100	6.29 ± 0.76
E100N	1.17 ± 0.21	22.94 ± 3.20[c]	n.d.	46.72 ± 10.23[a]	n.d.
D122S	1.07 ± 0.18	194 ± 17	85.5 ± 12.4	132 ± 37	401 ± 59.6[a]
D126S	0.97 ± 0.09	30.53 ± 4.33[b]	n.d.	25.72 ± 6.94[d]	n.d.
T150A	0.73 ± 0.08[b]	129 ± 14	11.7 ± 1.98	126.75 ± 13.19	92.1 ± 24.7
H158A	1.01 ± 0.15	51.66 ± 9.35	10.00 ± 4.47	57.45 ± 7.56[a]	20.6 ± 6.58
