# Supplementary material for: Structures of active melanocortin-4 receptor–Gs-protein complexes with NDP-α-MSH and setmelanotide
Source: Cell Res. 2021 Sep 24;31(11):1176–89. doi: 10.1038/s41422-021-00569-8 (PMC8563958; doi:10.1038/s41422-021-00569-8)
Supplement: Supplementary file 34 — Supplementary table S10 [file 41422_2021_569_MOESM34_ESM.pdf]

Table S10: ***Binding affinities and EC*** $_{50}$ ***values of diverse melanocortin ligands.*** Data were extracted from previous studies and combined with data (*) measured in this study.

**ligand**	**α-MSH [nM]**	**β-MSH [nM]**	**NDP-α-MSH [nM]**	**Setmelanotide [nM]**	**LY2112688 [nM]**
K_i or K_D	51[3]	20[16]	1[3]/1*	2.1[17]/1*	4[18]
EC_{50} (cAMP)	18*	33[19]	1*	1*	14[20]
EC_{50} (NFAT)	489[19]; 174[20]	107[19]	6*	6*	330[20]
