# Supplementary material for: Structures of active melanocortin-4 receptor–Gs-protein complexes with NDP-α-MSH and setmelanotide
Source: Cell Res. 2021 Sep 24;31(11):1176–89. doi: 10.1038/s41422-021-00569-8 (PMC8563958; doi:10.1038/s41422-021-00569-8)
Supplement: Supplementary file 35 — Supplementary Data S1 [file 41422_2021_569_MOESM35_ESM.pdf]

Supplemental Information
References for Supplemental Figures and Tables

1. McDonald IK, Thornton JM. Satisfying hydrogen bonding potential in proteins. *J Mol Biol* 1994; **238**:777-793.

2. Laskowski RA, Swindells MB. LigPlot+: multiple ligand-protein interaction diagrams for drug discovery. *J Chem Inf Model* 2011; **51**:2778-2786.

3. Yu J, Gimenez LE, Hernandez CC *et al.* Determination of the melanocortin-4 receptor structure identifies Ca(2+) as a cofactor for ligand binding. *Science* 2020; **368**:428-433.

4. Ballesteros JA, Weinstein H. Integrated methods for the construction of three-dimensional models and computational probing of structure-function relationships in G-protein coupled receptors. *Methods Neurosci* 1995; **25**:366-428.

5. Hall TA. BioEdit: a user-friendly biological sequence alignment editor and analysis program for Windows 95/98/NT. *Nucleic Acids Symposium Series* 1999; **Series 41**:95-98.

6. Hanson MA, Roth CB, Jo E *et al.* Crystal structure of a lipid G protein-coupled receptor. *Science* 2012; **335**:851-855.

7. Chrencik JE, Roth CB, Terakado M *et al.* Crystal Structure of Antagonist Bound Human Lysophosphatidic Acid Receptor 1. *Cell* 2015; **161**:1633-1643.

8. Rasmussen SG, DeVree BT, Zou Y *et al.* Crystal structure of the beta2 adrenergic receptor-Gs protein complex. *Nature* 2011; **477**:549-555.

9. Yin J, Chen KM, Clark MJ *et al.* Structure of a D2 dopamine receptor-G-protein complex in a lipid membrane. *Nature* 2020; **584**:125-129.

10. Liu K, Wu L, Yuan S *et al.* Structural basis of CXC chemokine receptor 2 activation and signalling. *Nature* 2020; **585**:135-140.

11. Okada T, Sugihara M, Bondar AN, Elstner M, Entel P, Buss V. The retinal conformation and its environment in rhodopsin in light of a new 2.2 A crystal structure. *J Mol Biol* 2004; **342**:571-583.

12. Kang Y, Kuybeda O, de Waal PW *et al.* Cryo-EM structure of human rhodopsin bound to an inhibitory G protein. *Nature* 2018; **558**:553-558.

13. Kim K, Che T, Panova O *et al.* Structure of a Hallucinogen-Activated Gq-Coupled 5-HT2A Serotonin Receptor. *Cell* 2020; **182**:1574-1588 e1519.

14. Israeli H, Degtjarik O, Fierro F *et al.* Structure reveals the activation mechanism of the MC4 receptor to initiate satiation signaling. *Science* 2021; **372**:808-814.

15. Winn MD, Ballard CC, Cowtan KD *et al.* Overview of the CCP4 suite and current developments. *Acta Crystallogr D Biol Crystallogr* 2011; **67**:235-242.

16. Yan LZ, Hsiung HM, Heiman ML *et al.* Structure-activity relationships of beta-MSH derived melanocortin-4 receptor peptide agonists. *Curr Top Med Chem* 2007; **7**:1052-1067.

17. Kumar KG, Sutton GM, Dong JZ *et al.* Analysis of the therapeutic functions of novel melanocortin receptor agonists in MC3R- and MC4R-deficient C57BL/6J mice. *Peptides* 2009; **30**:1892-1900.

18. Kievit P, Halem H, Marks DL *et al.* Chronic treatment with a melanocortin-4 receptor agonist causes weight loss, reduces insulin resistance, and improves cardiovascular function in diet-induced obese rhesus macaques. *Diabetes* 2013; **62**:490-497.

19. Paisdzior S, Dimitriou IM, Schope PC *et al.* Differential Signaling Profiles of MC4R Mutations with Three Different Ligands. *Int J Mol Sci* 2020; **21**.

20. Clement K, Biebermann H, Farooqi IS *et al.* MC4R agonism promotes durable weight loss in patients with leptin receptor deficiency. *Nat Med* 2018; **24**:551-555.
